# WS-SSA: workflow scheduling in cloud computing using salp swarm algorithm

**DOI:** 10.1038/s41598-026-48037-w

**Published:** 2026-04-24

**Authors:** Aya A. Sharawy, Rasha H. Sakr, Waleed Eladrosy, Mohammed F. Alrahmawy

**Affiliations:** 1 Department of Computer Science, Faculty of Computer and Information Sciences, Mansoura University, Mansoura, Egypt; 2 Faculty of Computer Science and Engineering, New Mansoura University, New Mansoura, Egypt; 3 VIZJA University, Warsaw, Poland; 4 Department of Computer Science, Higher Technological Institute (HTI), 10th of Ramadan City, Egypt

**Keywords:** Cloud computing, Workflow scheduling, Salp swarm algorithm, Makespan optimization, Scientific workflows, Engineering, Mathematics and computing

## Abstract

Effective process scheduling is mandatory in cloud computing. It optimizes the use of resources, reduces the execution time, and reduces the operating cost. This study introduces a new workflow scheduling algorithm based on the salp swarm algorithm (SSA). This algorithm is a nature-based metaheuristic and is based on salp swimming behavior in the ocean. The proposed scheduler is based on the use of a single-objective fitness function to optimize the overall workflow makespan. It solves the NP-hard cloud scheduling problem, which involves effective mapping of tasks to heterogeneous virtual machines. The workflow execution is represented via a directed acyclic graph (DAG). The SSA is very efficient in search space exploration and is insensitive to parameter choices. To evaluate the quality of the proposed scheduler, several experiments were conducted via the WorkflowSim simulation framework. The makespan and energy consumption are taken as important performance indicators in the assessment. The WS-SSA was evaluated against FCFS, MCT, MIN-MIN, MAX-MIN, round robin, the WOA, the GA, and PSO under identical VM settings and workflow inputs to ensure fairness. It reduces the average makespan by up to 38% compared with classical methods and approximately 8.7% compared with the WOA and approximately 5–22% and 3–12% over the GA and PSO, respectively.

**Supplementary Information:**

The online version contains supplementary material available at 10.1038/s41598-026-48037-w.

## Introduction

Cloud computing has transformed IT resource management, providing heterogeneous and scalable environments where workflow scheduling is critical for best task allocation, resource utilization, and quality of service (QoS) adherence^1^. Distributed execution of complex interdependent workflows via service models such as Infrastructure-as-a-Service (IaaS), Platform-as-as-Service (PaaS), and Software-as-a-Service (SaaS) and other new paradigms, such as Containers-as-a-Service (CaaS) the need to employ strategies that can reduce makespan and minimize energy and functional costs^2,3^. Workflow scheduling refers to the allocation of interdependent tasks to cloud resources to minimize the completion time and improve resource utilization. It is complicated by dynamic workloads, various service level agreements and variable availability of resources. The workflow scheduling problem is NP-hard, making exact optimization methods impractical for large-scale cloud environments^4–8^. As a result, heuristic and metaheuristic algorithms such as genetic algorithms (GAs), particle swarm optimization (PSO), and ant colony optimization (ACO) are popular, and they provide scalable algorithms that do not affect performance. Nevertheless, there are still difficulties with adaptive, real-time scheduling and multiobjective optimization in the case of dynamic cloud environments, and new research is being conducted to increase the efficiency, reliability and quality of service (QoS) of large-scale workflows.

This research proposes a workflow scheduling algorithm, named the WS-SSA, which is based on the salp swarm algorithm (SSA)^9^ for cloud computing environments. The algorithm maximizes the task allocation and sequence of execution among interdependent tasks on heterogeneous cloud resources. It has a relatively basic aim of minimizing a makespan, which is the total time spent by the execution of the first task to the completion of the last task. Energy consumption is assessed as a secondary performance metric alongside makespan to provide a more comprehensive understanding of the efficiency of a given scheduling and its quality.

The workflow scheduling problem addressed in this study follows the classical directed acyclic graph (DAG)-based formulation commonly adopted in the existing workflow scheduling literature. Therefore, the contribution of this work lies not in introducing a new theoretical scheduling model but rather in designing an effective adaptation of the Salp Swarm Algorithm for workflow-aware task scheduling in heterogeneous cloud environments. The proposed approach focuses on improving search efficiency and solution quality through algorithmic design and implementation within the established scheduling framework.

WS-SSA is especially ideal for cloud workflow scheduling, as it can support the heterogeneity of resources, be able to explore large solution spaces efficiently and address complex workflow scheduling problems without significant parameter tuning. The WS-SSA tends to reach near-optimal schedules faster than traditional and other metaheuristic algorithms, including genetic algorithms, particle swarm optimization, and ant colony optimization, so it is potentially useful in dynamic cloud-based scenarios where timely decision making is needed. Unlike existing SSA-based workflow schedulers that target homogeneous environments or simplified independent task models, the proposed WS-SSA schedules interdependent tasks structured as heterogeneous scientific workflow DAGs across heterogeneous VMs in cloud environments, optimizing the makespan across varying workflow sizes (25–1000 tasks). Most existing approaches assume task independence, ignoring intertask dependency constraints that are inherent in scientific workflows; in contrast, WS-SSA explicitly enforces dependency constraints through topological ordering prior to fitness evaluation, ensuring that all precedence relationships are respected in every candidate solution throughout the search. More distinctively, unlike metaheuristic algorithms, which operate in a continuous search space and require a separate decoding step, the WS-SSA adopts a native discrete integer representation where every position directly encodes a valid task-to-VM assignment, preserving feasibility by construction throughout the entire optimization process with zero decoding overhead. To assess the algorithm, a workflow simulation tool, WorkflowSim^10^, was used, where different workflow patterns were tested and compared to traditional methods of scheduling. The makespan was taken as the main optimization parameter, and energy consumption was identified as the secondary performance parameter. Our experimental data show that WS-SSA is typically more efficient in terms of lowering workflow completion time and energy efficiency under various conditions. However, latent constraints are sensitive to extremely large workflows and the dependency structure of tasks, which can influence scalability and execution-time performance. These aspects underscore future research areas, including strategies of adaptative and real-time scheduling and multiobjective optimization under dynamic conditions.

The remainder of the paper is organized as follows: The background and concepts of workflow scheduling in cloud computing are reviewed in Section 2. Section 3 provides an overview of the basic SSA. Section 4 presents related work in the field. The proposed scheduling problem is defined and formulated in Section 5. In Section 6, the proposed workflow scheduling algorithm with its various phases was presented. Section 7 explains the experimental setup and compares the work of the proposed scheduler with that of other scheduling methods. Finally, Section 8 presents the paper’s conclusions and outlines directions for future work.

## Background and preliminaries

Building on the scheduling challenges outlined above, this section presents the foundational concepts and formal definitions that underpin the proposed WS-SSA algorithm, including workflow modeling, DAG representation, and the cloud scheduling process.

### Workflow modeling and DAG representation

**Fig. 1:**
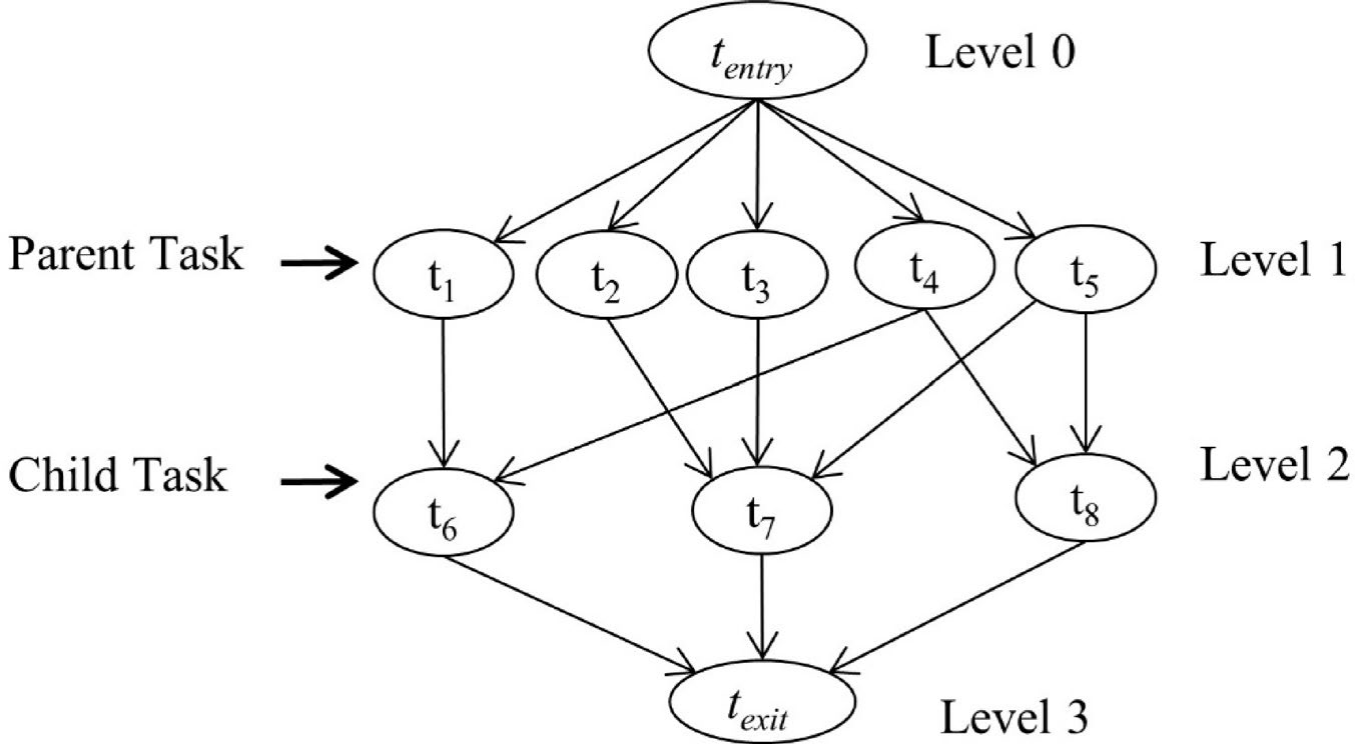
A DAG for a workflow.

A workflow consists of a set of interdependent tasks representing scientific or business applications. It is a directed acyclic workflow graph $\:W$ and is concerned with conditional dependencies. Every actual computational task appears as a node with links between tasks represented as edges. $\:W=\left\{T,\:E\right\}$, where *T* is the complete set of computation tasks denoted $\:T=\left\{{t}_{1},{t}_{2},......,{t}_{n}\:\right\}$, and where $\:E\subseteq\:TxT$ is the set of directed edges exactly as in Fig. 1. The edges indicate a data dependency between two tasks^11^. A directed edge $\:\left({t}_{i}\:,\:{t}_{j}\right)\in\:E\:$ indicates data dependency and is used to show that $\:{t}_{j}$ cannot start until $\:{t}_{i}$ is completed and that all the necessary data have been supplied. $\:{t}_{i}$ is the parent for $\:{t}_{j}$, and $\:{t}_{j}$ is the child of $\:{t}_{i}$. An entry task $\:{t}_{entry}\in\:T$ has no parents at all; on the other hand, the exit task $\:{t}_{exit}\in\:T$ does not have any children^12^. Each task has its size, which is measured in millions of instructions (MI). The cloud environment consists of data centers full of hosts, i.e., physical servers denoted as *H* that deliver CPU power, memory, storage space, and bandwidth^13^.

### Scheduling workflow in cloud computing

In cloud computing environments, workflow tasks are executed on heterogeneous virtual machines (VMs) with different processing capabilities, which have their own processing capabilities, to workflow tasks. The goal here is to reduce the total time it takes to finish the overall execution of the workflow^5–8,14,15^. Virtualization allows these hosts to execute multiple virtual machines $\:VM=\left\{{vm}_{1},\:{vm}_{2},.......,\:{vm}_{m}\right\}$ simultaneously, depending on their capacity^16–19^. The main execution unit workflow in the cloud environment is virtual machines. Nevertheless, they vary considerably in their performance features, such as processing capacity in millions of instructions per second (MIPS), memory capacity, storage capacity and network bandwidth. This heterogeneity adds much complexity to the task-resource mapping process because the choice of a VM to be utilized on each task has a direct effect on the efficiency of the entire workflow and its cost. A workflow management system (WMS) traditionally controls workflow execution, whereas a component broker that selects and schedules resources is the mediator between users and cloud providers^15^. The workflow scheduling process typically involves a number of steps: (1) resource provisioning and VM instantiation at the datacenter level, (2) workflow submission to the WMS, (3) dependency analysis and task prioritization, (4) resource discovery and scheduling by the broker, (5) task-to-VM assignment, and (6) continuous monitoring and adaptive adjustments to a failure or variation in runtime. It is a highly computationally intensive multistage process, and this scheduling process is computationally complex due to the large search space and task dependencies.

Consequently, traditional heuristics such as Min-Min, Max-Min and round robin tend to be less effective in large-scale highly dynamic cloud systems. To overcome these difficulties, recent studies have used metaheuristic methods, such as PSO and the whale optimization algorithm (WOA), and machine learning methods, especially reinforcement learning, to increase scheduling performance^20,21^. The objective of these methods is to strike a balance between various competing goals, including reducing makespan, increasing energy consumption, and decreasing the execution cost. Workflow scheduling has been a key research area in both cloud computing and edge computing because scheduling decisions play a crucial role in determining the performance of applications. Successful scheduling also includes the process of provisioning or renting the computing resources of IaaS providers to meet the computational and QoS demands of user-submitted scientific workflows^5^.

## Overview of the salp swarm algorithm (SSA)

Metaheuristic techniques have gained considerable popularity in recent years. This increase is due to several factors, including their flexibility, their ability to operate without gradient information, and their ability to avoid becoming trapped in local optima^9^. Unlike gradient-based optimization techniques, metaheuristics do not require internal analytical knowledge of the optimization problem but rely only on input–output evaluations, treating the problem as a black-box system^22^. Consequently, they do not require derivative computations within the search space, which is particularly beneficial in complex nonlinear or high-dimensional optimization problems. These characteristics enable metaheuristics to be applied to a wide variety of optimization problems. In addition, metaheuristic methods are stochastic optimization approaches that employ random operators, allowing them to escape local optima that frequently occur in real-world optimization landscapes.

The Salp Swarm Algorithm (SSA) is a swarm intelligence-based metaheuristic optimizer proposed by Mirjalili et al. in 2017 and inspired by the swarming behavior of salps moving and foraging in ocean environments, the Salp swarm algorithm is a comprehensive survey^9,23^. Salps belong to the Salpidae family and have transparent barrel-shaped bodies resembling jellyfish tissues. They propel themselves forward by pumping water through their bodies, a movement mechanism similar to that of jellyfish^24–26^. One of their notable natural behaviors is swarming, where individuals form a chain-like structure consisting of leaders and followers. In this structure, the leader occupies the front position and guides the movement of the swarm, while the remaining individuals follow sequentially.

The SSA optimizes candidate solutions by iteratively updating the positions of salps in the search space. The algorithm maintains the best solution found so far as the food source, which guides the movement of the leader salp during the search process. The leader explores the search space around the food source, whereas follower salps update their positions on the basis of the movement of the salp ahead of them in the chain^9,23,27,28^. Through this leader–follower interaction, the SSA effectively explores the search space while gradually guiding candidate solutions toward promising regions. The SSA achieves a balance between exploration and exploitation during the optimization process, where exploration allows the algorithm to investigate diverse regions of the search space, whereas exploitation focuses on refining promising solutions, thereby reducing the risk of premature convergence to local optima. The adaptive mechanism within the SSA encourages extensive exploration in the early stages of optimization and gradually shifts toward exploitation as the search progresses.

Several studies have demonstrated the effectiveness of the SSA in solving complex optimization problems and highlighted its notable strengths over other heuristic optimization methods^23^. For example, comparative studies have shown that the SSA can sample diverse regions of the search space while progressively improving the solution quality. This performance is attributed mainly to its strong exploration and exploitation capabilities, which enable efficient search and refinement within the solution space. In addition, the SSA has been reported to outperform several evolutionary and swarm-based algorithms in certain optimization scenarios, including the gravitational search algorithm (GSA)^29^ and GA. Moreover, the SSA requires fewer control parameters than many metaheuristic algorithms, such as the GA and PSO, do, which simplifies its implementation and reduces the risk of performance degradation due to poor parameter selection. Its simple structure and ease of integration with other heuristic strategies make it a flexible optimization method applicable to a wide range of complex optimization problems. Hybridization with other algorithms can further enhance its performance and accelerate convergence toward optimal solutions.

## Related work

Workflow scheduling in cloud computing has attracted significant research attention because of its critical role in improving system performance, minimizing execution time, and optimizing resource utilization. The primary challenge lies in efficiently allocating interdependent workflow tasks to heterogeneous computing resources while satisfying dependency constraints and optimizing multiple performance metrics, such as makespan, cost, energy consumption, and load balancing. To address these challenges, numerous optimization-based scheduling techniques have been proposed. Existing studies can generally be categorized into PSO-based approaches, ACO-based approaches, hybrid metaheuristic approaches, and other nature-inspired or heuristic scheduling strategies.

### PSO-based scheduling approaches

PSO has been widely applied in workflow scheduling because of its strong ability to explore large search spaces and optimize task–resource mappings. Researchers have widely adopted PSO-based methods for workflow scheduling because of their ability to balance exploration and exploitation while preserving workflow dependency constraints. Biswas et al. proposed PSO-based scheduling to represent candidate workflow schedules as particles whose positions and velocities are iteratively updated to minimize makespan and improve system utilization^30^. To increase the optimization performance, many researchers have proposed hybrid PSO-based scheduling strategies. In these approaches, PSO is typically employed to explore the search space and generate candidate scheduling solutions, while additional optimization techniques are incorporated to refine promising solutions. For example, hybrid frameworks that combine PSO with gray wolf optimization (GWO) were proposed by Arora and Banyal to improve the balance between exploration and exploitation during the optimization process^31^. Similarly, other studies have integrated PSO with ant lion optimization (ALO) to increase the solution quality while reducing the makespan and execution cost in heterogeneous cloud environments^32^. In addition to these hybridizations, PSO has also been combined with heuristic task-ranking mechanisms and evolutionary optimization techniques. For example, the HEFT-PSO-GA (HEPGA)^33^ scheduling framework integrates HEFT-based^34^task prioritization with PSO, a hybrid multiobjective particle swarm optimization for scientific workflow scheduling^35,36^. and GA^37^ to improve task–resource mapping and scheduling efficiency. Overall, these PSO-based and hybrid PSO strategies demonstrate the potential of swarm intelligence techniques to improve workflow scheduling performance in distributed cloud computing environments. However, a common limitation across these approaches is that increased hybridization complexity may reduce computational efficiency, particularly in large-scale scheduling scenarios.

### ACO-based scheduling approaches

ACO has also been widely adopted for workflow scheduling because of its effectiveness in solving combinatorial optimization problems through pheromone-based search mechanisms. In ACO-based scheduling approaches, artificial ants construct candidate task–resource mappings, whereas pheromone updates guide the search toward promising scheduling solutions. Several improvements to the ACO^38^ algorithm have been proposed to enhance scheduling performance. For example, the inverted ant colony optimization (IACO) algorithm modifies the traditional pheromone update mechanism by reducing pheromone intensity on frequently selected paths to encourage exploration and prevent premature convergence^39^. Hybrid scheduling strategies that combine ACO with heuristic task ranking methods have also been developed. In particular, the HEFT–ACO^40^ scheduling approach integrates the HEFT ranking strategy^41^ with ACO-based resource allocation, where HEFT is first used to determine task priorities on the basis of execution and communication costs, followed by ACO-based optimization to map tasks efficiently to virtual machines. These ACO-based approaches demonstrate strong performance in discovering efficient scheduling paths while preserving workflow dependencies. Nevertheless, ACO-based approaches are generally sensitive to pheromone parameter tuning, which may affect their generalizability across different workflow types.

### Hybrid metaheuristic scheduling approaches

Hybrid metaheuristic algorithms have gained increasing attention for workflow scheduling because they combine complementary optimization strategies to balance global exploration and local exploitation during the search process. By integrating multiple optimization mechanisms, hybrid approaches aim to overcome the limitations of individual metaheuristic algorithms. Researchers have developed hybrid metaheuristic frameworks as effective strategies for improving workflow scheduling performance by combining complementary optimization mechanisms. For example, a hybrid gray wolf optimization (HGWO) algorithm^42^ enhances scheduling performance by combining the exploration capability of GWO^43^ with the local search strength of simulated annealing (SA)^44^. Similarly, hybrid evolutionary approaches such as the GASA algorithm integrate the GA with SA to improve search efficiency and reduce the risk of premature convergence in heterogeneous cloud environments^45^. Other studies have explored hybrid swarm-intelligence-based optimization techniques. For example, the enhanced binary artificial bee colony-based Pareto front algorithm (EBABC-PF)^46^ integrates HEFT-based task prioritization with binary artificial bee colony optimization^47,48^ to reduce the makespan and execution cost while increasing resource utilization. Similarly, hybrid scheduling frameworks that combine the Bat algorithm^49^ with HEFT have been proposed to optimize workflow scheduling while considering multiple objectives, such as makespan, cost, and energy consumption^50^. These hybrid metaheuristic approaches demonstrate improved scheduling performance by leveraging the strengths of multiple optimization strategies. A consistent finding across these hybrid approaches is that combining a priority-based heuristic such as HEFT with a population-based metaheuristic yields better scheduling quality than either method in isolation.

### Other metaheuristic and heuristic scheduling approaches

In addition to the PSO and ACO-based approaches, numerous other meta-heuristic and heuristic scheduling techniques have been investigated for workflow scheduling in heterogeneous computing environments. Some studies focus on heuristic scheduling strategies that improve task allocation efficiency. For example, Kamanga et al. proposed a scheduling optimization technique that dynamically selects CPU frequencies to reduce execution cost^51^, whereas Rajak et al. introduced a heuristic-guided breadth-first search method that prioritizes workflow tasks according to their dependencies to minimize makespan^52^. Other studies have explored various nature-inspired optimization algorithms for workflow scheduling. The WOA has been applied to optimize task scheduling by considering metrics such as makespan, migration time, and energy consumption^53,54^. Similarly, cuckoo search-based scheduling algorithms have been proposed to reduce SLA violations and improve energy efficiency^55^. Several studies have also investigated multiobjective optimization techniques for workflow scheduling. For example, Paknejad et al. proposed a multiobjective workflow scheduling approach based on the preference-inspired coevolutionary algorithm (PICEA-g) to simultaneously optimize the makespan, execution cost, and energy consumption^56^. Jaiprakash et al. addressed energy-aware scheduling via the energy-efficient workflow scheduling (EEWS) approach, which employs a critical path-based flower pollination algorithm^57^ to prioritize workflow tasks and optimize both execution time and energy consumption through adaptive fitness evaluation^58^. Similarly, the intelligent water drop-based cloud scheduling algorithm (IWDC)^59^ uses path-construction mechanisms inspired by the intelligent water drop optimization method^60^ to identify efficient task–virtual machine mappings while preserving workflow dependencies. In the context of energy-efficient cloud scheduling, Javadpour et al. proposed two DVFS-based task scheduling methods, GIoTDVFS_SFB and GIoTDVFS-mGA, for managing IoE tasks in cloud platforms, achieving notable reductions in energy and power consumption^61^. In a related study, Javadpour et al. further proposed an energy-optimized embedded load balancing approach using DVFS computing in cloud data centers, demonstrating improvements in both energy consumption and power efficiency through dynamic task prioritization and VM migration^62^. In addition to cloud workflow scheduling, task scheduling has also been studied in heterogeneous and multicore computing systems. For example, Chakraborty et al. proposed the temperature-aware scheduler TREAFET^63^, whereas Sharma and Moulik introduced several scheduling techniques, including RT-SEAT^64^, HEAT^65^, and FRESH^66^, to address challenges related to energy efficiency, thermal management, and fault tolerance in heterogeneous computing platforms. Collectively, these studies reflect the growing trend toward energy-aware and multiobjective scheduling, highlighting the need for algorithms capable of balancing multiple competing objectives simultaneously.

Despite the extensive research on workflow scheduling in cloud environments, several limitations remain. Many existing approaches focus primarily on single-objective optimization, particularly minimizing makespan, whereas fewer studies address more complex scheduling scenarios that involve balancing multiple performance metrics simultaneously. In addition, several metaheuristic algorithms suffer from limitations related to premature convergence or insufficient exploration of the search space, which may lead to suboptimal scheduling solutions in highly heterogeneous cloud environments. Furthermore, although hybrid optimization strategies have improved scheduling performance in many studies, the design of efficient swarm-based algorithms that effectively balance exploration and exploitation while maintaining computational efficiency remains an open research challenge. These limitations motivate the development of improved swarm intelligence-based workflow scheduling algorithms capable of producing high-quality scheduling solutions in heterogeneous cloud computing environments.

## Problem formulation

The workflow scheduling problem involves assigning an appropriate virtual machine to each task in the workflow. This assignment takes the form of a mapping function, as shown in Eq. (1), where each task $\:{t}_{i}\in\:T$ is mapped to a virtual machine $\:v{m}_{j}\in\:VM$. It must follow the resource capacity constraint and task dependencies in the form of equations in the form of Eq. (2). This paper focuses on minimizing the makespan of the workflow. Workflow tasks in the workflow can be executed in parallel in virtual machine instances of the same level.

1 $$\:f\::\:T\:\to\:\:VM\:$$

2 $$\:ST\left({t}_{j}\right)\ge\:FT\left({t}_{i}\right)\:,\:\:\:\:\forall\:\left({t}_{i}\:,\:{t}_{j}\right)\in\:\:E$$

The execution time $\:ET$ to execute a task $\:{t}_{i}$ on a particular virtual machine $\:{vm}_{j\:}$, which is represented by Eq. (3), is expressed in terms of the number of task instructions (MI) divided by the virtual machine’s CPU capacity$\:\:\left(C\right)\:$, which is measured in MIPS.

3 $$\:ET({t}_{i}\:,\:{vm}_{j\:})=\frac{MI\left({t}_{i}\right)}{C\left({vm}_{j\:}\right)}$$

The start time $\:ST\left({t}_{i},{vm}_{j}\right)$ of each task is governed by two factors: the completion times of its predecessor tasks and the availability of the assigned virtual machine. For entry tasks, tasks with no predecessors—both quantities default to zero—yield a start time of zero. For all other tasks, the start time is determined by the unified formulation in Eq. (4). The accumulated load $\:AL\left(v{m}_{j}\right)$ represents the earliest time at which virtual machine $\:v{m}_{j}$ becomes available to process a new task. It is defined as the maximum finish time among all tasks previously assigned to that VM. At the beginning of the scheduling process, $\:AL\left(v{m}_{j}\right)=0$ for all VMs reflects their initial idle state. After each task assignment, $\:AL\left(v{m}_{j}\right)$ is updated to reflect the finish time of the most recently completed task on that VM. This mechanism ensures that a task can only start execution when both its predecessors have completed and the assigned virtual machine becomes available.

4 $$\:ST\left({t}_{i},{vm}_{j}\right)=\mathrm{m}\mathrm{a}\mathrm{x}\left({max}_{{t}_{p}\in\:\:predecessor\:of\:{t}_{i}}\:FT\right({t}_{p}),AL({vm}_{j}\left)\right)\:$$

Once the start time of each task is obtained, the finish time $\:FT\left({t}_{i},\:{vm}_{j}\right)$ can be calculated Eq. (5). The makespan of the workflow, which represents the completion time of the final (exit) task, is determined according to Eq. (6). The main objective of the scheduling process is therefore to minimize the makespan. Notably, energy consumption is not incorporated into the optimization objective but is evaluated as a secondary performance metric to assess the energy efficiency of the proposed scheduling approach.

5 $$\:FT\left({t}_{i},\:{vm}_{j}\right)=ST\left({t}_{i},{vm}_{j}\right)+ET\left({t}_{i},{vm}_{j}\right)\:$$

6 $$\:min\:Makespan={max}_{{t}_{i}\in\:\:{T}_{exit}\:}\left\{FT\left({t}_{i}\right)\right\}$$

In this study, the workflow scheduling problem is evaluated under a static cloud environment as provided by the WorkflowSim simulation framework. The set of virtual machines is assumed to remain available throughout the execution of the workflow, and task execution is performed without simulated failures. Consequently, dynamic events such as virtual machine failures, task failures, or runtime changes in resource availability are not explicitly modeled in the current implementation. This assumption is consistent with many WorkflowSim-based scheduling studies, where the primary objective is to compare scheduling strategies under controlled and reproducible experimental conditions.

### The proposed workflow scheduler

**Fig. 2:**
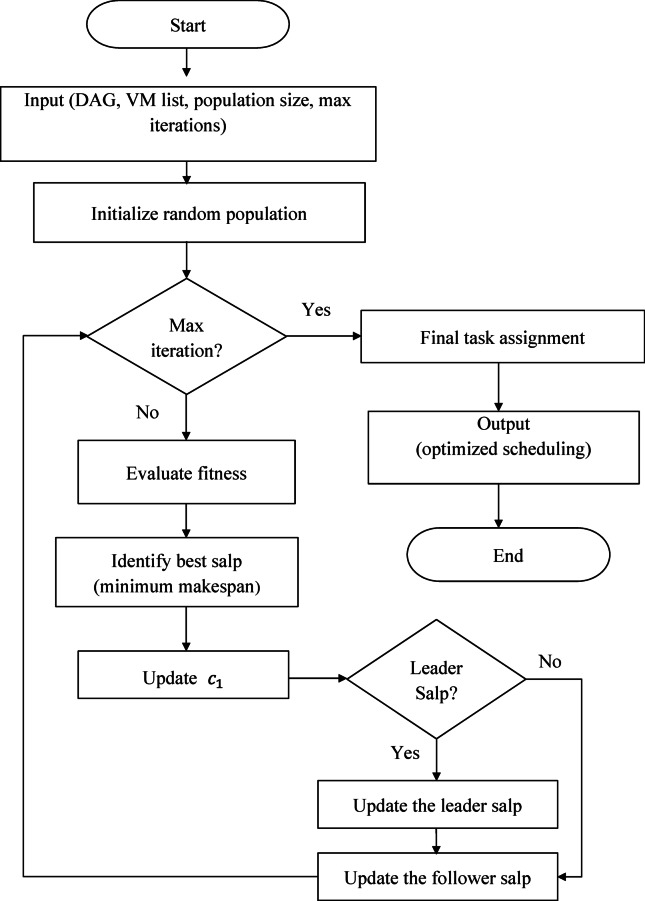
Flow chart of the proposed WS-SSA workflow scheduler in cloud computing.

In this paper, we propose a workflow scheduler for scheduling workflows in cloud computing environments via the salp swarm algorithm (WS-SSA), as presented in Algorithm1, which optimizes the scheduling of the execution of different workflow tasks by mimicking the movement of salps in nature to assign computational tasks (cloudlets) to virtual machines in a manner that seeks to minimize the makespan. This section demonstrates the process of scheduling workflows on the cloud via WS-SSA. The algorithm can be outlined in three major steps: the initialization phase, the adaptive evolutionary search phase, and convergence with the task assignment phase. The following subsections present the phases of this algorithm in detail. Figure 2 represents the flowchart of the WS-SSA workflow scheduler.

#### Initialization phase

In the initial phase, a population of candidate scheduling solutions, referred to as salps, is generated. Every salp is a possible mapping of all workflow tasks to the existing virtual machines in the cloud infrastructure. This mapping is coded as a position vector, with every element being the virtual machine index of a task. In a workflow composed of $\:n$ tasks and $\:m$ virtual machines, a salp $\:S$ is represented as $\:S=\left[{x}_{1},\:{x}_{2},\dots\:,{x}_{n}\right]$, where the value of $\:{x}_{i}$ represents the index of the VM allocated to task $\:{T}_{i}$. Salps are randomly initialized to provide the population with diversity in the initial search space, which is imperative for exploring various areas of the solution space and preventing premature convergence. Each salp’s position vector is generated by randomly assigning each task to one of the available VMs. Once the salps have been initialized, their fitness is determined via the quality of the scheduling they represent. The fitness function in workflow scheduling is expressed as the makespan in the form of Eq. (6). The salp with the minimum fitness (smallest makespan) is determined as the current best solution and stored as the food source position $\:\overrightarrow{\mathrm{F}}$, which represents the target toward which the salp swarm will move in further iterations and will lead the search to the optimal or near-optimal scheduling configuration.

#### Adaptive evolutionary search phase

The WS-SSA is based on its evolutionary process, which is iterative and is based on the dynamics between the leader and followers, as seen in swarms of natural salp. This is done by repeatedly updating the population (generations), and each salp changes its assignment of tasks to the VM as it tries to reduce the quality of its scheduling via coordinated movement patterns. The leader salp of the population is the first salp and performs exploration of the search space by updating its position (task-to-VM assignment) according to the current global best solution (food source $\:\overrightarrow{F}$), by a stochastic mechanism defined by Eq. (7), such that $\:{x}_{i}^{t+1}$ is the updated VM assignment for task $\:{T}_{i}$ in the leader salp at iteration $\:t+1$, and $\:{F}_{i}$ is the point of reference for exploration that is VM assigned to task $\:{T}_{i}$ in the current best solution, which is used to act as an attractor that guides the search toward optimal regions. The important aspect in this update formulation is the coefficient $\:{c}_{1}$​, which is an adaptive exploration–exploitation factor, as presented in Eq. (8). At early iterations, $\:{c}_{1}$​​ is large, which entices the algorithm to explore new and potentially better regions that are distant from the current best solution. This wide exploration helps the population avoid being trapped in local optima. As the iterations increase, $\:{c}_{1}$ exponentially approaches zero, causing the movement of the leader to become more conservative and focused on optimizing its position around the best-discovered solution; therefore, a global exploration phase gives way to a local exploitation phase. The leader’s movement becomes more conservative, and the leader focuses on refining its position around the best solution found thus far.

**Algorithm 1:**
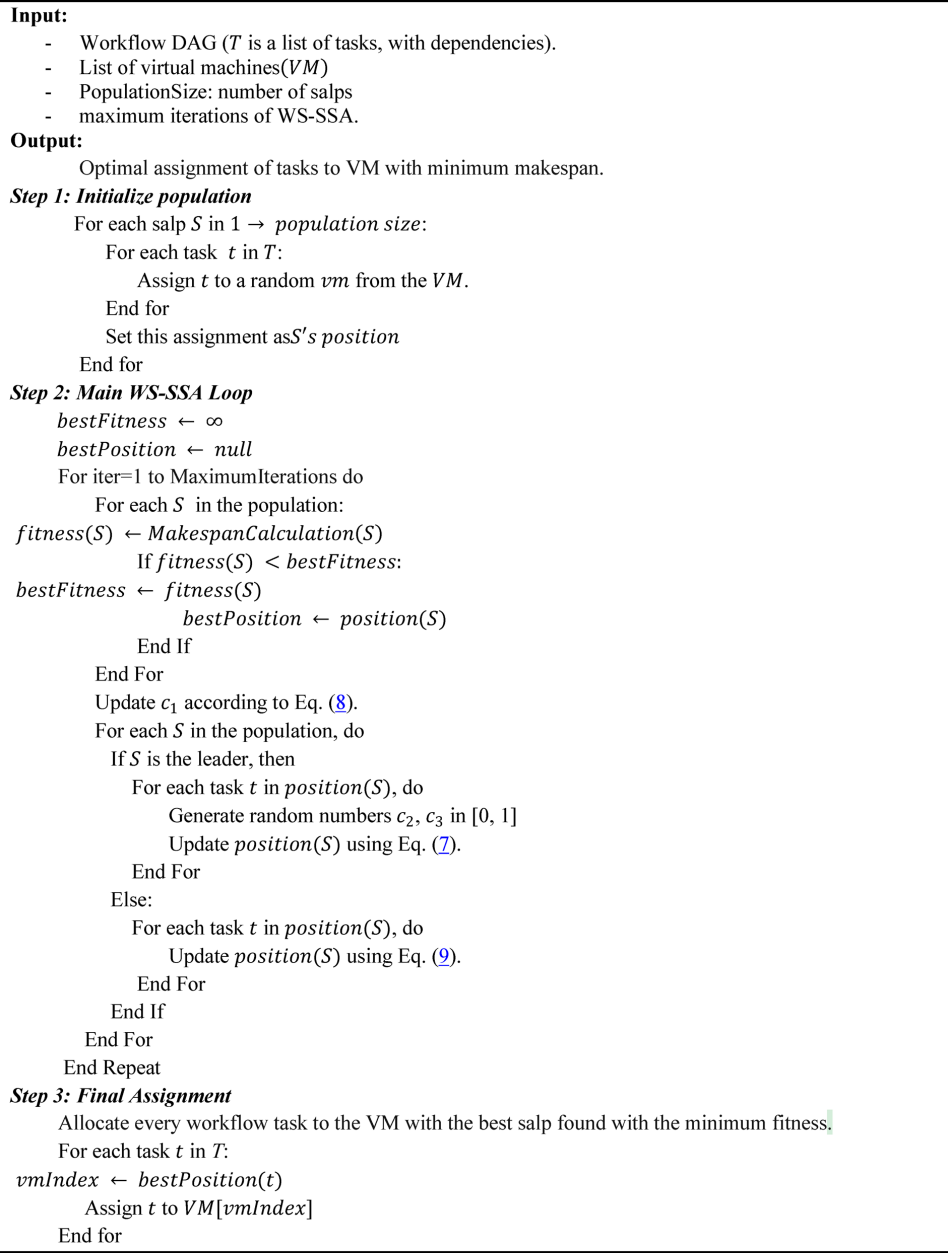
Proposed WS-SSA workflow scheduling algorithm in cloud computing.

7 $$\:{x}_{i}^{t+1}=\left\{\begin{array}{c}{F}_{i}+{c}_{1}\left(\left(ub-lb\right){c}_{2}+lb\right)\:\:\:\:if\:{c}_{3}\ge\:0.5\\\:{F}_{i}-{c}_{1}\left(\left(ub-lb\right){c}_{2}+lb\right)\:\:\:\:if\:{c}_{3}<0.5\end{array}\right.\:\:\:\:$$

The random parameters $\:{c}_{2}$ and $\:{c}_{3}$ in Eq. (7) are necessary to provide the diversity and directionality in the search. The movement step of the salp agents around the food source is modulated by a parameter $\:{c}_{2}$ in the range [0,1]. When the value of $\:{c}_{2}$ is larger, there is wider search space exploration because more scheduling structures are constructed, whereas when it is smaller, the emphasis is on exploiting preferred solutions that are fine-grained to minimize the makespan. The parameter that determines whether the salp leader moves toward or away from its food supply is the parameter $\:{c}_{3}$, which, again, lies in the range [0,1]. To eliminate mathematical inconsistency in the original formulation of the SSA, the threshold comparison of $\:{c}_{3}$ was set to 0.5 instead of 0 to allow better exploration. The proposed WS-SSA guarantees that both forward and backward movements around the food source have equal chances (50%), thus supporting directional randomness and eliminating algorithmic bias.

This bidirectional search mechanism plays a fundamental role in workflow scheduling in cloud environments, where the space of solutions has several local optima because the dependencies between tasks are complex and resources have heterogeneous properties. The balanced directional control allows the leader salp to avoid suboptimal regions, identify better task-to-VM mappings and eventually enhance the quality of convergence and scheduling efficiency. There exists a limit of a search space $\:ub$ (maximum VM index m) and $\:lb$ (minimum VM index, usually 1), such that the results of a search are defined by the calculated range between the maximum and minimum indices $\:[ub,\:lb]$. The algorithm ensures that all the virtual machine indices produced are restricted to fall within the given range of $\:[ub,\:lb]$. The $\:\left(ub-lb\right){c}_{2}+lb$ expression leads to the generation of a random VM index in this valid range, which has controlled stochasticity and satisfies constraints within the system.

8 $$\:{c}_{1}=2{e}^{{-\left(\frac{4t}{T}\right)}^{2}}$$

The followers are the salps that follow the leader within the chain. The positions are updated by follower salps according to Eq. (9), where $\:{x}_{i}^{t+1}$ is the new VM assignment of task $\:{T}_{i}$ of the current follower salp, $\:{x}_{i}^{t}$ represents the current VM assignment of this follower salp and $\:{x}_{i-1}^{t}$ represents VM assignment of the previous salp (closer to leader), averaging it with the salp ahead of them, which encourages convergence toward promising regions (the best-known solutions), and further refines the task assignments to further minimize the fitness function. These follower updates cement exploitation by refining the good solutions that already exist in the process of local search. The arithmetic mean of the current and preceding positions that move the follower halfway between its current position and the preceding salp, which has an impact on convergence that achieves gradual movement toward the leader’s region, prevents oscillation and promotes stability, creates a smooth convergence trajectory. The followers refine the solutions by averaging, searching intensely for promising regions.

9 $$\:{x}_{i}^{t+1}=\left({x}_{i}^{t}+{x}_{i-1}^{t}\right)/2\:$$

Salps (leader and followers) update their positions in an iteration, and the fitness of each salp is recalculated by computing the makespan of its task-to-VM assignment, as shown in Eq. (10). The best global solution is updated if any salp achieves a lower makespan than the current best solution according to Eq. (11). This process is carried out at a specified number of iterations$\:T$.

10 $$\:Fitness\left({S}^{t+1}\right)=Makespan\left({S}^{t+1}\right)\:$$

11 $$\:if\:\:Fitness\left({S}_{best}^{t+1}\right)<Fitness\left(\overrightarrow{F}\right)\:\:\:then\:\:\:\:\overrightarrow{F}\leftarrow\:{S}_{best}^{t+1}$$

Thus, there is a natural transition from exploration (global search) to exploitation (local search and convergence). At this stage, the optimal solution identified is constantly monitored and revised. This phase dynamically balances broad exploration of the search space and intensive exploitation around the current best solution, such that it is instrumental in achieving robust workflow scheduling in cloud computing. The exploration phase helps the algorithm evade local minima and provides a global search for effective task allocations. Moreover, the exploitation stage exploits the identified promising regions during the exploration stage and successively optimizes schedules to increase performance metrics such as execution time and energy efficiency. This global search versus local optimization trade-off has a direct influence on the efficiency of the algorithm. The WS-SSA adopts a native discrete representation in which each salp position directly encodes a complete task-to-VM assignment as an integer vector over the range [0, m − 1], where m denotes the number of available virtual machines. Unlike metaheuristic algorithms, which operate in a continuous search space and require a separate decoding step to map real-valued positions to discrete scheduling decisions, the WS-SSA initializes and maintains integer-valued positions throughout the entire optimization process. The leader update computes an integer-valued displacement derived from the adaptive coefficient c1 and applies it directly to the current best position, whereas the follower update averages adjacent salp positions via integer arithmetic. In both cases, a boundary correction mechanism immediately clamps all resulting values to the valid range [0, m − 1], ensuring that every generated solution corresponds to a legitimate VM assignment without requiring any post hoc feasibility repair. Constraint consistency is further enforced by combining this boundary correction with a topological ordering of workflow tasks prior to fitness evaluation, guaranteeing that all intertask dependency constraints are respected in every candidate solution evaluated throughout the search. Consequently, feasibility is preserved by construction rather than by heuristic approximation, and every position update produces a valid, directly executable scheduling solution with no additional decoding overhead.

#### Convergence and task assignment phase

When the algorithm reaches the maximum number of iterations, it moves on to the last phase. The best solution (i.e., salp with the minimum makespan) is selected because it is the best or nearly optimal schedule that has been found. Tasks in the workflow are assigned by encoding salps in task-to-virtual machine mapping, which completes the scheduling process. This phase ensures that all workflow tasks are allocated to virtual machines in a manner that represents the optimized execution time as identified by the WS-SSA.

## Experiments and results

CloudSim has become one of the most popular and extensible cloud-computing environment simulation frameworks. Approximately 57% of the other simulators extend it in some way^67,68^. Nevertheless, it has real limits in regard to simulations that handle only individual jobs or tasks with it^69^. Therefore, it is necessary to look for a tool that addresses workflows appropriately. One that manages dependencies between those jobs and tasks. Therefore, researchers turned to the use of WorkflowSim for this purpose^10^. It was built right on top of CloudSim. It represents scientific workflows and executes them as DAGs in the cloud computing platform. WorkflowSim follows the approach of Pegasus WMS^70^. It contains a workflow mapper, plus an engine and scheduler and a clustering engine, as described in^71^. As a whole, it is built over CloudSim, and it supports Pegasus workflows. Five well-known scientific applications were added to this simulator. They vary in their construction and levels of parallelism. These include LIGO Inspiral analysis^72,73^, Montage^74,75^, CyberShake^76^, Epigenomics^77^, and SIPHT^78^. WorkflowSim supplies such DAX files. XML-based DAX files are convertible to DAG workflows via workflow management system tools such as Pegasus^70^. We used WorkflowSim to evaluate the proposed workflow scheduling algorithm. Compared with several established scheduling methods. We performed our experiments over a system running on a PC with an 11th Gen Intel(R) Core (TM) i7-1165G7 CPU@ 2.80 GHz, an RAM of 8.00 GB and a 64-bit Windows operating system.

### Performance metrics

The workflow scheduling performance in the cloud is covered in this section. It shows key metrics such as energy consumption and makespan. The impact of various virtual machine configurations on overall computing efficiency is thus the main focus. The makespan is then expressed in seconds, as explained in the above section. Makespan is the amount of time that passes between the beginning of one task and the completion of the last task. It is therefore a simple method for evaluating the performance of scheduling algorithms in cloud systems. Energy consumption is the total power consumed by all the virtual machines in terms of joules. The energy model that we use is that suggested by^79^, which houses total energy consumption into passive and active components. The implementation follows Eqs. (12)-(18). The total energy consumption of workflow execution is determined on the basis of two different components: passive energy consumption and active energy consumption. The passive energy is used throughout the time of turning on the VM up to the time of turning it off. For a given solution $\:S$, the passive energy consumption $\:{E}_{p}\left(S\right)$ is defined as in Eq. (12), where $\:M\:$ is the set of all virtual machines utilized in the infrastructure and where $\:{P}_{p}\left(k\right)$ is the passive power of virtual machine $\:v{m}_{k}$. Active power consumption is the extra energy that will be needed to complete computational workloads on the VMs. This energy is determined by the processing properties of each task and the power consumption profile of the VM that is executing the task. The active energy consumption $\:{E}_{a}(i,k)$ for executing task $\:{t}_{i}$ on virtual machine $\:v{m}_{k}$ is defined as in Eq. (13), where $\:{P}_{a}\left(k\right)$ is the active power of virtual machine $\:v{m}_{k}$ when processing tasks. $\:\left(In\right(i,k)+ET(i,k)+Out(i,k\left)\right)$ represents the total time that the VM is actively consuming power to perform task $\:{t}_{i}$^79^.

12 $$\:{E}_{p}\left(S\right)=\sum\:_{k\in\:M}{P}_{p}\left(k\right)*makespan$$

13 $$\:{E}_{a}\left(i,k\right)={P}_{a}\left(k\right)*\left(In\left(i,k\right)+ET(i,k)+Out\left(i,k\right)\right)$$

where $\:In(i,k)$ denotes the time required for task $\:{t}_{i}$ executed on $\:v{m}_{k}$ to receive input data from its predecessor tasks and is calculated via Eq. (14), where $\:{DTT}_{l,k}^{j,i}$ is the data transfer time from task $\:{t}_{j}$ (executed on $\:v{m}_{l}$) to task $\:{t}_{i}$ (executed on $\:v{m}_{k}$), which is calculated via Eq. (15), where $\:D(i,j)$ represents the size of the data to be transmitted from $\:{t}_{i}$ to $\:{t}_{j}$, where $\:{ds}_{k}\:$ is the $\:{vm}_{k}$ disk speed and where $\:{nb}_{k}$ and $\:{nb}_{l}$ are the network bandwidths of $\:{vm}_{k}$ and $\:{vm}_{l}$, respectively. A local disk read is needed in data transfer when tasks $\:{t}_{i}$ and $\:{t}_{j}$ are hosted on the same VM$\:(l=k)$. Otherwise, if $\:(l\ne\:k)$, then $\:{nb}_{k}$ and $\:{nb}_{l}$ must also be considered^79^.

14 $$\:In\left(i,k\right)=\sum\:_{{t}_{j}\in\:pred\left({t}_{i}\right)}{DTT}_{j,i}^{l,k}\:$$

15 $$\:{DTT}_{j,i}^{l,k}=\left\{\begin{array}{c}\frac{D\left(i,j\right)}{{ds}_{k}}\:\:\:\:\:\:\:\:\:\:\:\:\:\:\:\:\:\:\:\:\:\:\:\:\:\:\:if\:k=l\\\:\frac{D(i,j)}{\mathrm{min}\left({ds}_{k},{nb}_{k},{nb}_{l}\right)}\:\:\:\:\:\:\:\:\:\:\:if\:k\ne\:l\end{array}\right.\:\:$$

where $\:Out\left(i,k\right)$ denotes the time required for task $\:{t}_{i}$ on VM $\:v{m}_{k}$ to write its output data to the local disk, which is calculated via Eq. (16). The total active energy consumption $\:{E}_{a}\left(S\right)$ for a solution $\:S$ is the sum of the active energy consumed by all tasks calculated via Eq. (17)^79^.

16 $$\:Out\left(i,k\right)=\frac{\sum\:_{{t}_{j} \in succ\left({t}_{i}\right)}D(i,j)}{{ds}_{k}}$$

17 $$\:{E}_{a}\left(S\right)=\sum\:_{i \in T,<i,k>\in\:Hosts}{E}_{a}(i,k)$$

Following an additive energy model, the total energy consumption $\:TE\left(S\right)$ of a workflow schedule solution $\:S$ is calculated via Eq. (18). Detailed energy analysis shows the effectiveness of WA-SSA in minimizing the power requirement. It maintains strong performance at the same time. This is true for various scientific workflows^79^.

18 $$\:TE\left(S\right)={E}_{p}\left(S\right)+{E}_{a}\left(S\right)$$

### Experimental infrastructure configuration

To comprehensively evaluate the performance of the proposed WS-SSA scheduler, two complementary experimental studies were conducted. The first experiment (Experiment 1) benchmarks the WS-SSA against six widely adopted scheduling algorithms—FCFS, MCT, MIN-MIN, MAX-MIN, round robin, and WOA—across five standard scientific workflow types via the WorkflowSim simulation framework under (5, 10 and 15 VMs). The second experiment (Experiment 2) provides rigorous statistical validation through 30 independent runs per algorithm, comparing the WS-SSA against three additional metaheuristic competitors, the WOA, the GA, and PSO (50 and 100 VMs), and employs standard statistical measures, including the mean, standard deviation, 95% confidence interval, t test, and one-way ANOVA. Together, these two experiments establish both the broad superiority of WS-SSA over classical and contemporary scheduling methods and the statistical robustness of its performance advantage.

The WOA is selected as the benchmark meta-heuristic for comparing the performance of the SSA in workflow scheduling in cloud computing on multiple foundations. First, both algorithms are part of the swarm intelligence family and are based on the behavior of marine life, which guarantees a methodologically sound framework of comparison^9,80^. Second, the comprehensive validation and thriving literature of the WOA indicate its efficacy in tackling major scheduling issues, including makespan reduction, balanced resource use, and minimal energy usage^81^. Third, its simplicity and ease of implementation, along with its rapid convergence characteristics and flexibility to dynamic cloud environments, is a robust and reproducible benchmark^81,82^. Fourth, the fact that the WOA has been extensively hybridized and modified to enhance both exploration and exploitation abilities illustrates the fact that the algorithm is mature and highly adaptable, thus offering a solid reference upon which more innovative methods such as the SSA can be gauged^81,83^. Fifth, the WOA is now a standardized benchmark algorithm in cloud workflow scheduling studies, and it has been heavily validated on standard scientist workflows such as Montage, Epigenomics, and CyberShake^54,84^. In addition to the WOA, the GA and PSO are also incorporated as benchmark metaheuristics to provide a broader and more comprehensive evaluation of the proposed WS-SSA. The GA is one of the earliest and most widely adopted evolutionary algorithms and is known for its strong global search capability through genetic operators such as selection, crossover, and mutation. Its extensive application in workflow scheduling problems, particularly for minimizing makespan and improving resource allocation, makes it a classical baseline for performance comparison. On the other hand, PSO represents another well-established swarm intelligence algorithm inspired by the social behavior of bird flocking. It is recognized for its fast convergence speed and simplicity, as well as its effectiveness in continuous optimization problems, including cloud workflow scheduling. PSO has been widely validated in the literature for achieving competitive results in terms of makespan and energy efficiency. By including the GA and PSO alongside the WOA, the evaluation framework covers diverse categories of metaheuristic optimization techniques, including evolutionary-based (GA) and swarm-based methods (PSO and WOA). This diversity ensures a fair, comprehensive, and well-balanced comparison, allowing the effectiveness of the proposed WS-SSA algorithm to be assessed against both classical and state-of-the-art optimization approaches under identical experimental conditions.

The experiments used nonhomogeneous virtual machine setups with different computing capabilities to provide close-to-real-world cloud computing conditions. For each workflow DAX file available in the WorkflowSim dataset repository in^10^, we repeated all the experiments using different numbers of virtual machines, as specified in each experimental scenario. Table 1 shows the experimental simulator’s characteristics, with the number of virtual machines varying depending on the experiment. Furthermore, the bandwidth and speed of VMs are altered for all VMs via heterogeneous configurations. The assessment will include two key critical performance metrics that are fundamental in the optimization of cloud computing: makespan minimization and energy consumption reduction. The test was performed on an advanced heterogeneous cloud computing environment that simulates the conditions of a data center. Table 1 shows the infrastructure configurations of experiments 1 and 2. In both experiments, a cloud environment consisting of 20 heterogeneous hosts is considered, where each host operates at 2,000 MIPS per core with a 10 Gbps bandwidth. The host power consumption is modeled as 120 W in the passive state and 250 W in the active state. Virtual machines are configured heterogeneously, with MIPS values randomly distributed within the range [500–1,500] and bandwidths within [500–1,500] Mb/s, while maintaining fixed specifications of 512 MB RAM and 10 GB storage per VM. The VM energy consumption model assumes 10 W of passive and 35 W of active power, with a disk speed of 150 Mb/s. The two scenarios differ in their resource capacity and scaling setup. In Experiment 1, each host is equipped with 2 processing elements (PEs) and 2,048 MB of RAM, representing a relatively constrained environment under the VM configuration (5, 10 and 15 VMs). In contrast, Experiment 2 scales the infrastructure to a higher-capacity setting, where each host includes 4 PEs and 8,192 MB of RAM to support more intensive workloads under the VM configuration (50 and 100 VMs). Furthermore, in Experiment 2, each metaheuristic algorithm (WS-SSA, WOA, GA, and PSO) is executed over 30 independent runs for each workflow–VM configuration to ensure the robustness and statistical validity of the results.

The parameters of the metaheuristic algorithms were adapted to workflow size as summarized in Table 2: the population size and maximum number of iterations were set to 30/100, 50/200, and 80/300 for workflows with ≤ 30, 30–100, and > 100 tasks, respectively, ensuring a fair and reproducible comparison. The stopping rule for all algorithms was defined as reaching the maximum number of iterations. This setup facilitates the overall analysis of performance in terms of not only the execution time but also the energy efficiency measures.

### Experiment 1: Makespan performance analysis

**Fig. 3:**
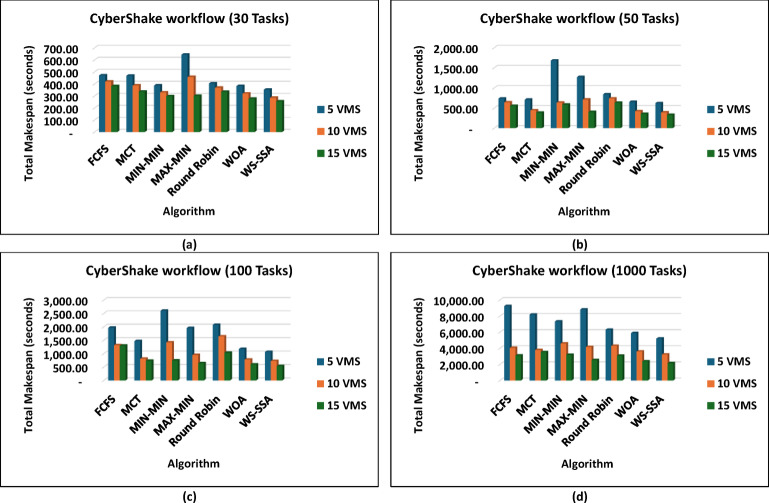
Superiority of the proposed WS-SSA scheduler over baseline and metaheuristic algorithms in reducing the makespan of CyberShake workflows of various sizes and using different VM configurations.

For experiment 1, the WS-SSA algorithm is superior to the other algorithms in terms of minimizing the total workflow completion time on different virtual machine configurations. The experimental assessment of a variety of scientific workflows provides strong proof of the superiority of the proposed WS-SSA algorithm in makespan minimization. WS-SSA has repeatedly defeated the other competing algorithms on computationally challenging CyberShake workflows, as shown in Fig. 3, which model seismic hazard assessments. The algorithm drastically improved the makespan to 353.13 s at smaller scales, such as CyberShake_30 with 5 VMs, surpassing 12.76% in comparison to Round Robin and surpassing 25.09% in comparison to FCFS. Compared with more powerful heuristics such as MCT, MIN-MIN, and MAX-MIN, WS-SSA showed reductions of 8.87% to 45.17%, and notably, the improvement was nearly 7.81% compared with WOA. With notable improvements over both heuristics and metaheuristics, the WS-SSA maintained its dominance at the largest scale (CyberShake_1000 with 15 VMs), demonstrating its capability.

**Table 1:** Simulation of the configuration setting.

Parameter	Experiment1	Experiment2
Number of data centers	1	1
Number of hosts	20	20
Host RAM	2,048 MB	8192 MB
Host storage	1,000,000 MB	1,000,000 MB
Host bandwidth	10,000 Mb/s	10,000 Mb/s
PEs per host	2	4
Host MIPS	2,000	2,000
Host power (passive/active)	120 W/250 W	120 W/250 W
Number of VMs	5, 10, 15	50, 100
PEs per VM	1	1
VM MIPS	500–1,500	500–1,500
VM bandwidth	500–1,500 Mb/s	500–1,500 Mb/s
VM RAM	512 MB	512 MB
VM storage	10,000 MB	10,000 MB
VM power (passive/active)	10 W/35 W	10 W/35 W
VM disk speed	150 Mb/s	150 Mb/s

**Table 2:** Parameter configuration of metaheuristic scheduling algorithms (SSA, WOA, PSO, and GA) on the basis of workflow size. All four algorithms adopt the same adaptive parameter settings to ensure fair comparison across experimental scenarios.

Workflow size (tasks)	Population size	Max iterations
≤ 30	30	100
30–100	50	200
> 100	80	300

**Fig. 4:**
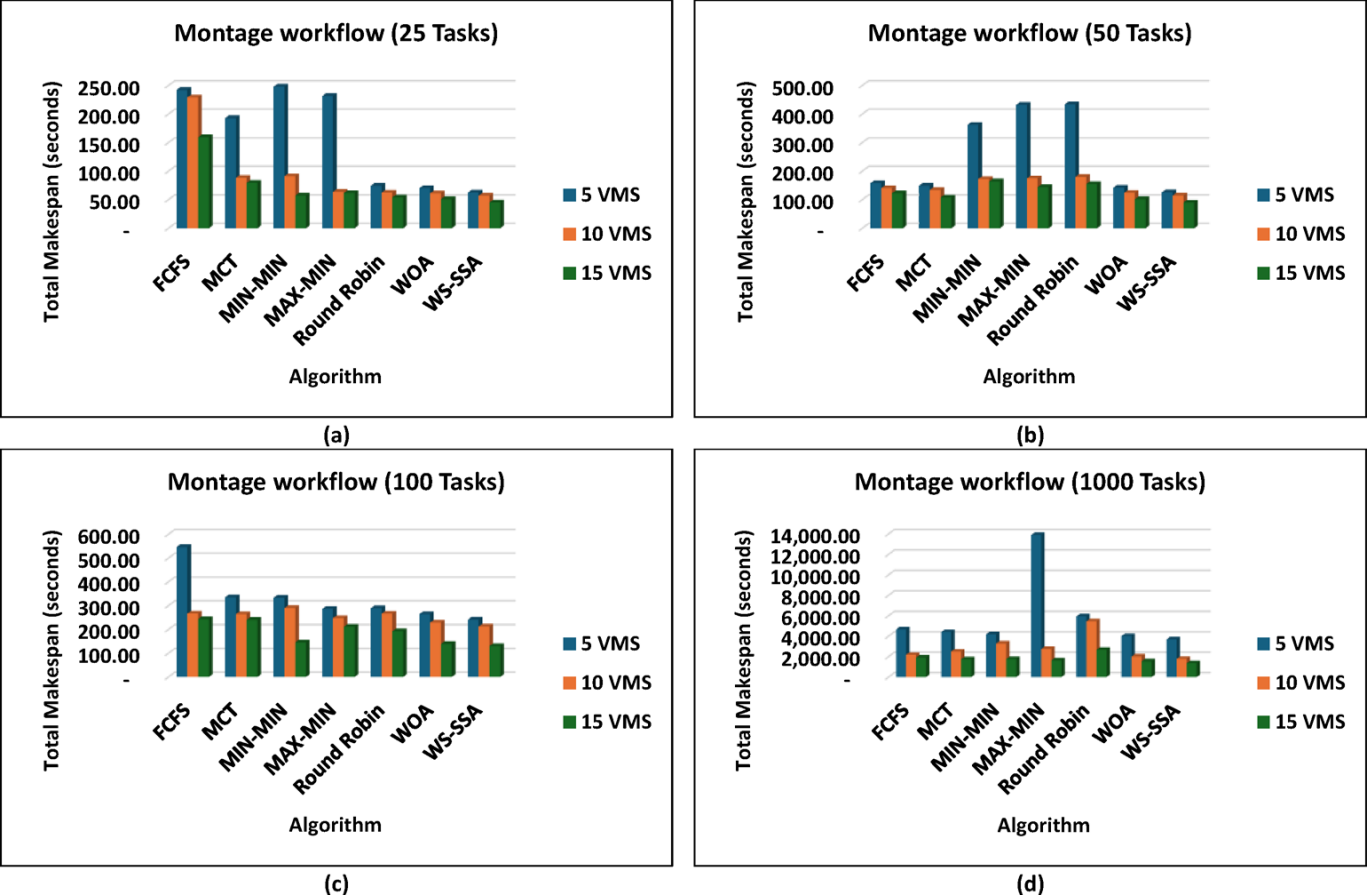
Superiority of the proposed WS-SSA scheduler over baseline and metaheuristic algorithms in reducing the makespan of Montage workflows of various sizes and using different VM configurations.

**Fig. 5:**
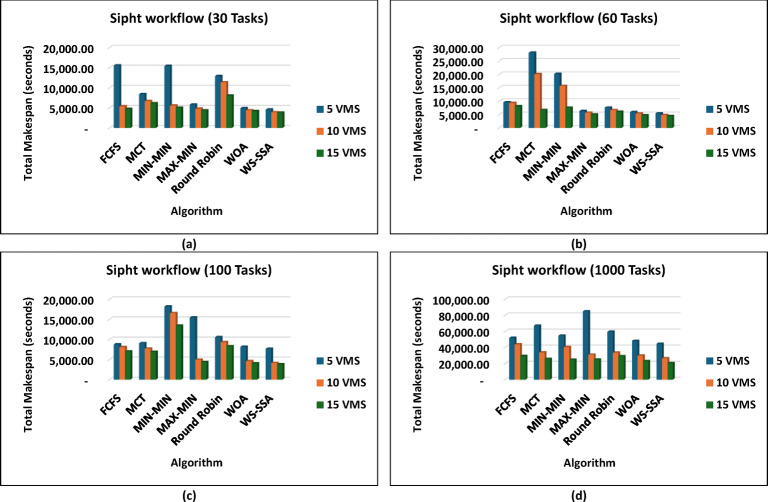
Superiority of the proposed WS-SSA scheduler over baseline and metaheuristic algorithms in reducing the makespan of the Sipht workflows of various sizes and using different VM configurations.

**Fig. 6:**
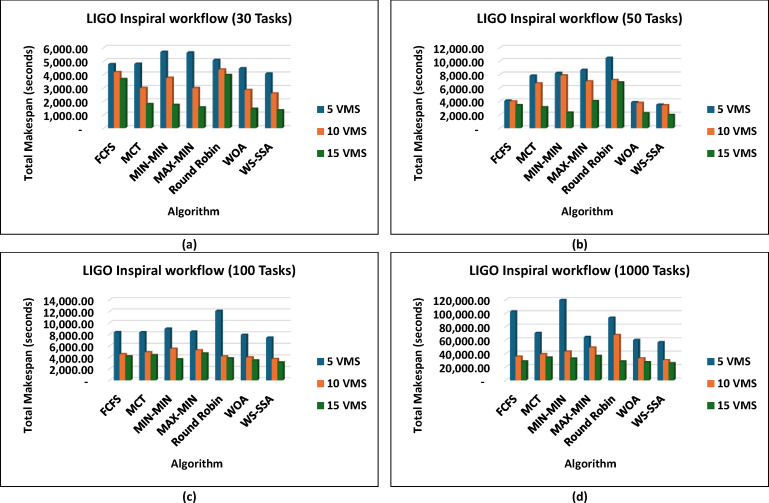
Superiority of the proposed WS-SSA scheduler over baseline and metaheuristic algorithms in reducing the makespan of the LIGO Inspiral workflows of various sizes and using different VM configurations.

**Fig. 7:**
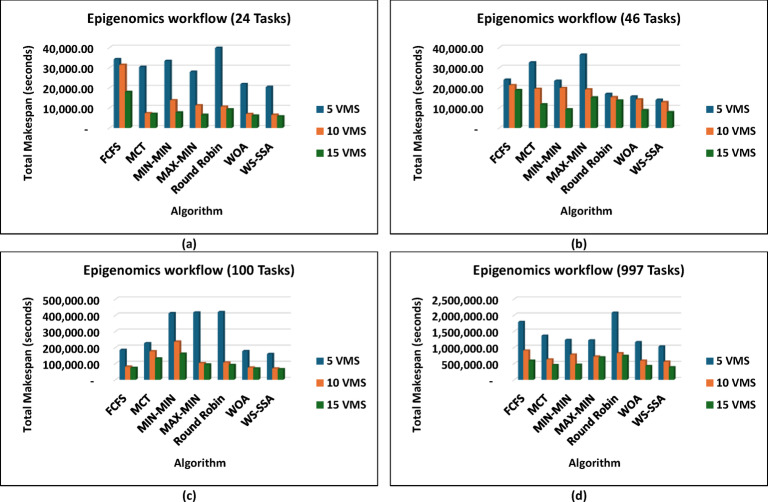
Superiority of the proposed WS-SSA scheduler over baseline and metaheuristic algorithms in reducing the makespan of the Epigenomics workflows of various sizes and using different VM configurations.

The WS-SSA algorithm drastically minimized the completion time of all workflow sizes, i.e., Montage_25 through Montage_1000, in the Montage workflows, as shown in Fig. 4, which are necessary in the construction of astronomical mosaics. Montage_25 with 5 VMs, for example, decreases the makespan to 62.58 s. This is quicker than what MCT and FCFS manage. In contrast to fancier heuristics such as MIN-MIN and MAX-MIN, the gains reach nearly 72.98% to over 74.77%. The scaled-up WS-SSA maintained a small advantage over the WOA, which indicates that the tendency of the algorithm to improve with scale. The Montage_1000 case has 15 vms, and its makespan is 1,363.28 s, which is better than those of the old standard methods. Overall, the results demonstrate that WS-SSA is effective in speeding up data-intensive imaging tasks that need to be repeated. The value of WS-SSA is further enhanced by the examination of SIPHT workflows, as shown in Fig. 5, which are crucial for bioinformatics and phylogenetic tree construction. A remarkable 64.51% decrease was observed compared with Round Robin, whereas a smaller but 6.88% improvement was observed compared with WOA. This steady benefit compared with WOA indicates a steady pattern of performance in place of individual improvements. Compared with 29,395.16 for FCFS and 24,784.54 for MIN-MIN, WS-SSA maintained its lead at the largest scale (SIPHT_1000 with 15 VMs). This difference can be translated into thousands of computation units saved and vastly accelerated to large-scale bioinformatics tasks. WS-SSA was once again the most effective scheduling method for inspiral workflows, as shown in Fig. 6, which are devoted to the analysis of gravitational wave data. The makespan decreased to 4,063.51 s at Inspiral_30 with 5 VMs, which is an improvement of 15.31% against MCT and nearly 14.76% against FCFS. Additionally, the WS-SSA continuously outperforms the WOA, MIN-MIN, and MAX-MIN. This margin was maintained across scales as opposed to varying, implying that the WS-SSA adequately balances exploration and exploitation in its search space. The use of WS-SSA to achieve 25,353.74 s at Inspiral_1000 using 15 VMs was faster than the heuristic bound and noticeable over the metaheuristic bounds. Finally, WS-SSA once again demonstrated ground-breaking efficiency in the epigenomics workflows, as is clear in Fig. 7, which are distinguished by highly interdependent and data-intensive genomic tasks. The makespan was decreased to 20,327.39 s in Epigenomics_24 with 5 VMs, greatly surpassing MIN-MIN by 39.02%, round robin by 48.97%, FCFS by 40.57% and MCT by 32.99%. The WS-SSA performed better than the WOA did, confirming that it provides better optimization for a variety of workload configurations. The makespan was decreased to 384,499.02 s in the largest Epigenomics_997 scenario, which is a quarter improvement over FCFS and nearly half the execution time compared with MIN-MIN. These findings are indications of WS-SSA performance in navigating and optimizing simulated large-scale bioinformatics workflows to achieve a reduction in the simulated makespan, suggesting the potential to accelerate scientific workflows in practice.

Makespan is impacted by the overall performance of workflow scheduling algorithms in cloud environments, which is strongly influenced by the number of virtual machines allotted. information about how various algorithms make use of more computing power. Across all the assessed algorithms, the results show a definite inverse relationship between makespan and the number of provisioned virtual machines. This is reasonable because increased processing power generally enables increased parallelization and a reduced waiting time. The WS-SSA with CyberShake_1000 decreases to 3,222.7 s when 10 VMs are used, which keeps it ahead of the pack. The increment from 5 to 10 VMs indicates how well the WS-SSA adjusts its scheduling to take advantage of more parallel processing. There are indications of workable scalability here; tasks are distributed effectively across additional resources to reduce waiting time and increase total output. Moving to 15 VMs further decreases the makespan of WS-SSA to 2,144.8 s. This gradual reduction by the addition of more machines indicates that it has an advantage in working with fine-grained parallelism in workflows. Other algorithms also perform better, but their improvement is more modest, and their makespan remains significantly greater overall. FCFS, for example. It shortens the makespan, although not to the full extent of the added resources, and this demonstrates its inefficiency in more complex scheduling problems. This tendency suggests that WS-SSA is a sophisticated method of on-the-fly resource allocation and task priorities; therefore, it utilizes additional computing capacity to the maximum. This strategy is appropriate for flexible cloud configurations, where rapid growth is very important. Table 3 summarizes the mean percentage improvements achieved by the WS-SSA over all baseline algorithms across makespan and energy consumption metrics, with detailed per-scenario results provided in the Supplementary Materials.

**Table 3:** Mean percentage improvements and ranges of the proposed algorithm compared with those of six baseline schedulers (FCFS, MCT, MIN-MIN, MAX-MIN, round robin, and WOA) for makespan and energy consumption metrics across all the experiments.

Algorithm	Makespan Mean improvements	Makespan ranges	Energy consumption mean improvements	Energy consumption ranges
FCFS	35.8%	9.1% – 79.3%	37.2%	6.5% – 72.5%
MCT	30.4%	10.6% – 80.7%	24.9%	3.1% – 80.0%
MIN-MIN	37.7%	8.9% – 75.0%	35.6%	4.4% – 73.2%
MAX-MIN	31.4%	10.6% – 73.4%	29.5%	7.0% – 73.6%
Round Robin	37.4%	8.4% – 71.3%	41.7%	8.2% – 76.6%
WOA	8.7%	5.0% – 12%	5.3%	0.3% – 8.0%

### Experiment 1: Energy consumption performance analysis

**Fig. 8:**
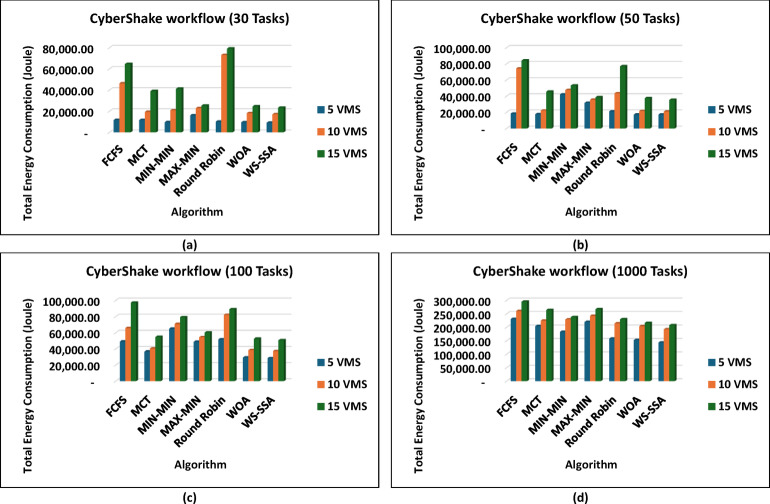
Superiority of the proposed WS-SSA scheduler over baseline and metaheuristic algorithms in reducing the energy consumption of CyberShake workflows of various sizes and using different VM configurations.

**Fig. 9:**
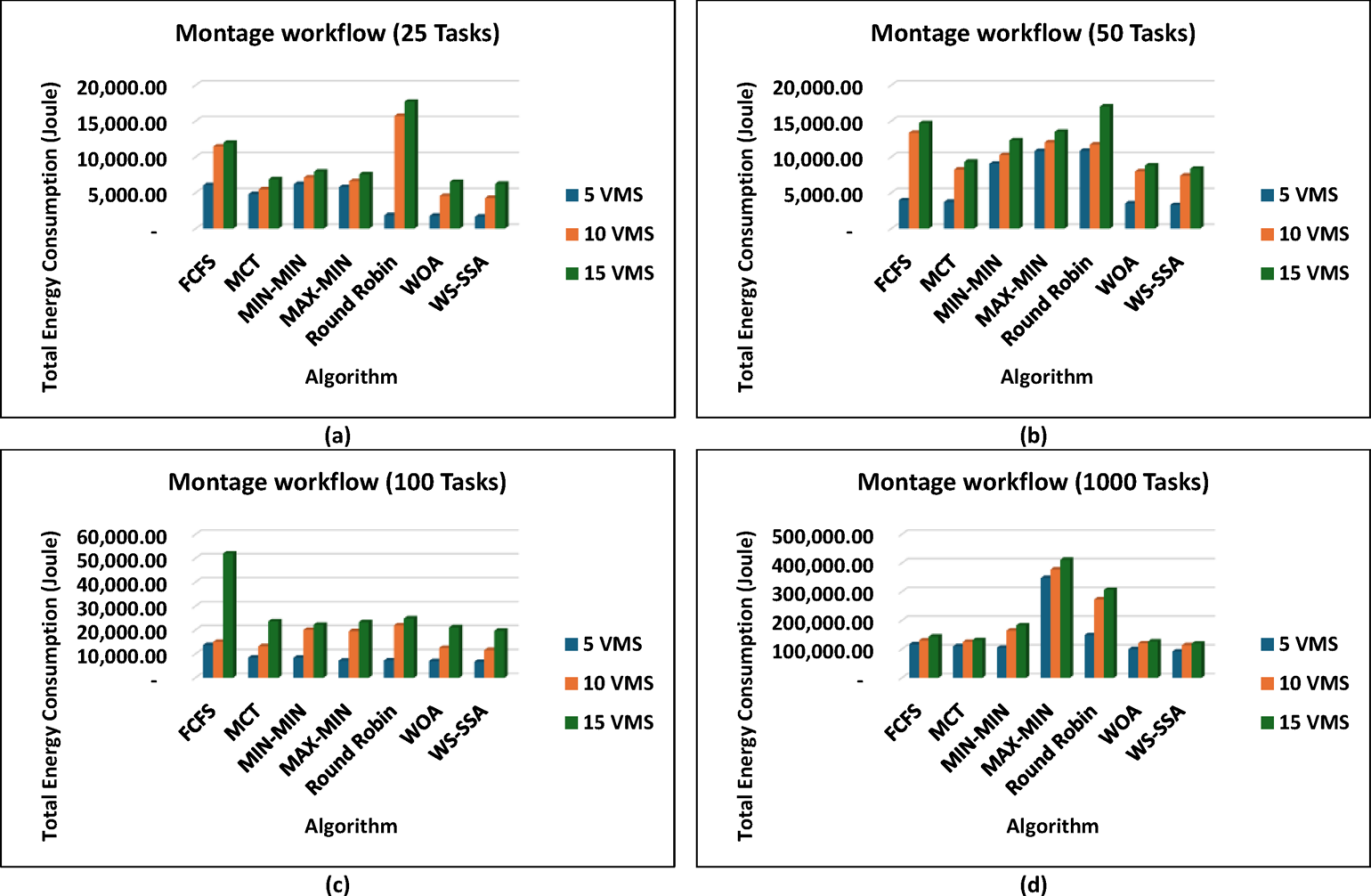
Superiority of the proposed WS-SSA scheduler over baseline and metaheuristic algorithms in reducing the energy consumption of Montage workflows of various sizes and using different VM configurations.

In parallel to how it performs in terms of makespan, the WS-SSA achieves good results in terms of reducing energy use. As shown in the supplementary results, WS-SSA consistently achieves the lowest energy consumption across all tested workflows, outperforming the old heuristics and other metaheuristic approaches every time. Such CyberShake workflows, e.g., Fig. 8, make it obvious that WS-SSA pulls ahead, even on smaller setups. Because CyberShake_30 runs with just 5 VMs, it uses only 9,258.5 joules, which is an 8.51% lower score than Round Robin and a 21.44% better score than FCFS. It even outperforms fancier approaches such as WOA in terms of energy. For CyberShake_1000 on 15 VMs, WS-SSA recorded 208,140.36 joules, which is 12.59% under MIN-MIN and 29.61% below FCFS. All this points to WS-SSA maintaining steady energy savings, regardless of the workload size. The Montage workflows show a similar pattern as in Fig. 9 in that WS-SSA uses only 1,661.74 joules in Montage_25 with 5 VMs, resulting in significant reductions of 72.54% over FCFS and 65.55% over MCT. With an energy consumption of 119,349.78 joules, the WS-SSA maintained its superiority when scaling up to Montage_1000 with 15 VMs. This is 16.91% less than that of FCFS and almost 5.63% more efficient than the WOA.

**Fig. 10:**
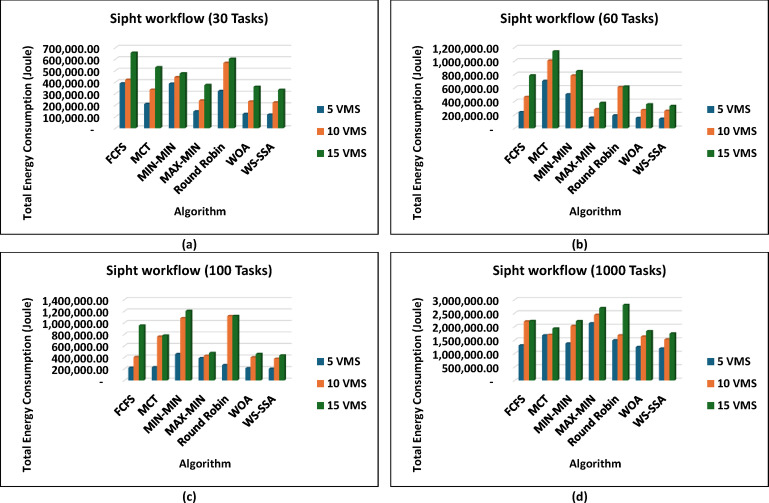
Superiority of the proposed WS-SSA scheduler over baseline and metaheuristic algorithms in reducing the energy consumption of sipht workflows of various sizes and using different VM configurations.

**Fig. 11:**
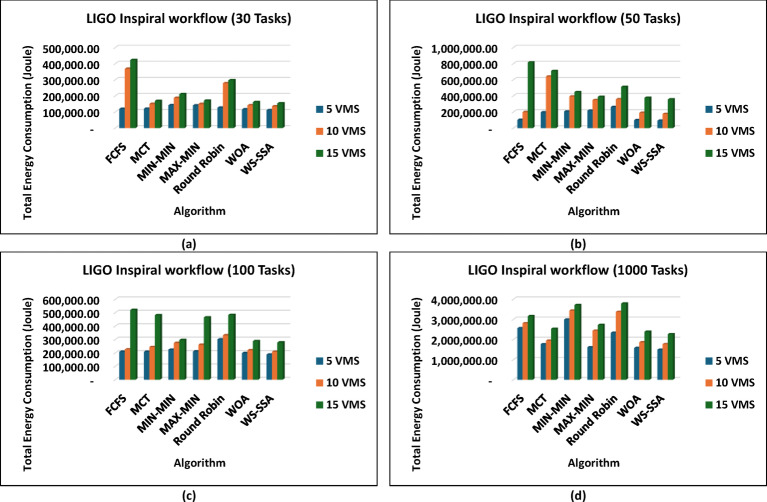
Superiority of the proposed WS-SSA scheduler over baseline and metaheuristic algorithms in reducing the energy consumption of LIGO Inspiral workflows of various sizes and using different VM configurations.

**Fig. 12:**
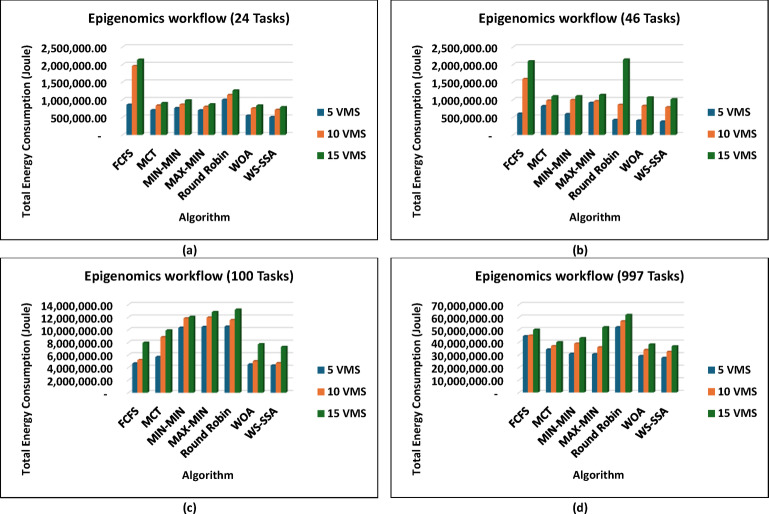
Superiority of the proposed WS-SSA scheduler over baseline and metaheuristic algorithms in reducing the energy consumption of the Epigenomics workflows of various sizes and using different VM configurations.

These results prove that WS-SSA is competitive with both other metaheuristics and can decrease the energy consumption of large-scale image processing tasks. The outcomes in Fig. 10 further highlight how consistently WS-SSA performs in the case of Sipht workflows. Sipht_30 with 5 VMs used 117,501.04 joules, which was 69.73% better than FCFS and 44.12% better than MCT. WS-SSA maintained an efficiency of 1,736,259.12 joules even in larger configurations, such as Sipht_1000 with 15 VMs, reducing energy use by 21.06% against MIN-MIN and by almost 21.25% against FCFS. These results indicate that the scalability of WS-SSA in reducing the energy load of bioinformatics workflows with environmentally friendly computing is particularly important. The results from Inspiral workflows are comparable to those in Fig. 11; WS-SSA was able to achieve an energy consumption of 110,865.27 joules for Inspiral_30 with 5 VMs, which was 12.61% lower than that of Round Robin and 7.57% better than that of MCT. WS-SSA achieved 2,262,110.38 joules at the larger Inspiral_1000 scale using 15 VMs, which is 39% higher than MIN-MIN and 28.35% higher than FCFS.

These findings demonstrate that WS-SSA is able to achieve great energy savings without performance deterioration in astrophysical applications that consume a large number of resources. Energy consumption analysis highlights the key point of view of the trade-off that exists in the optimization of makespan and the management of energy expenditure in cloud computing. Although increasing the number of VMs tends to reduce makespan, this is accompanied by increasing active resources, which, as a rule, increases the total energy consumption.

An important observation from the energy model used is the inherent relationship between total energy consumption and workflow makespan. Since passive energy is directly proportional to makespan, as given by Eq. (12), strategies that reduce makespan can simultaneously reduce passive energy consumption. Nevertheless, this process is not straightforward, as the active energy component is affected by the power of the selected VMs. High-performance VMs can decrease makespan and hence passive energy and can have higher active energy because of the higher power consumption rates. Thus, a balance between these competing factors must be achieved carefully to determine an optimal energy-efficient solution. The efficiency of an algorithm in this case is to minimize this energy increase and still attain performance objectives. For example, in Montage_100, which has energy consumption at 5 heterogeneous VMs, WS-SSA has the lowest energy consumption. This is a remarkable result because it displays the ability of the WS-SSA to schedule energy consumption even at reduced resource scales and outperforms the WOA and vastly outperforms the highly inefficient FCFS and round robin methods. This implies that time is not the only factor that WS-SSA uses in making scheduling decisions but that it implicitly considers resource utilization patterns that will result in reduced energy footprints. The energy consumption of all the algorithms, including the WS-SSA, is bound to increase as the number of VMs increases to 10. This apparent increase, however, should not be misinterpreted as inefficiency. Rather, it is just a manifestation of the basic fact that more actively used computational resources require more power. The most important point here is that the WS-SSA is still the most resource-efficient algorithm with the lowest energy consumption among all the tested algorithms at this VM count. Its resource efficiency can lead to a significant makespan reduction and, at the same time, the lowest energy used per unit of work or per performance gain.

### Experiment 2: Statistical Comparison of the WS-SSA Against the WOA, GA, and PSO Over 30 Runs

To situate WS-SSA within a broader landscape of established metaheuristic methods, a second experimental study was designed. Each of the four algorithms—WS-SSA, WOA, GA, and PSO—was independently executed 30 times per workflow–VM configuration. For each configuration, the mean makespan and mean energy consumption, along with their standard deviations (Stds) and 95% confidence intervals (CIs), were recorded. The statistical significance of the observed performance differences was assessed via both a two-tailed independent samples t test (with unequal variance assumed, i.e., Welch’s t test) and a one-way analysis of variance (ANOVA). The null hypothesis in both tests assumes no significant difference among the algorithms’ mean makespan values; a result is deemed statistically significant when the corresponding p value falls below α = 0.05. The energy consumption model employed in this study derives energy directly from the makespan through the passive–active energy formulation Eq. (18). Accordingly, energy consumption is not treated as an independent optimization objective but rather as a deterministic function of makespan and a complementary performance indicator reflecting resource efficiency. Consequently, any improvement in makespan inherently leads to proportional reductions in energy consumption. This dependency is further confirmed by the statistical analysis, where the ANOVA results (Table 4) yield identical F statistics and p values for both makespan and energy across all workflow configurations. Similarly, the statistical significance observed through t test analysis is consistent for both metrics, and the relative ranking of algorithms in terms of energy efficiency mirrors that of makespan. Therefore, reporting the statistical results for makespan is sufficient to infer the corresponding behavior of energy consumption .

### One-way ANOVA results

**Fig. 13:**
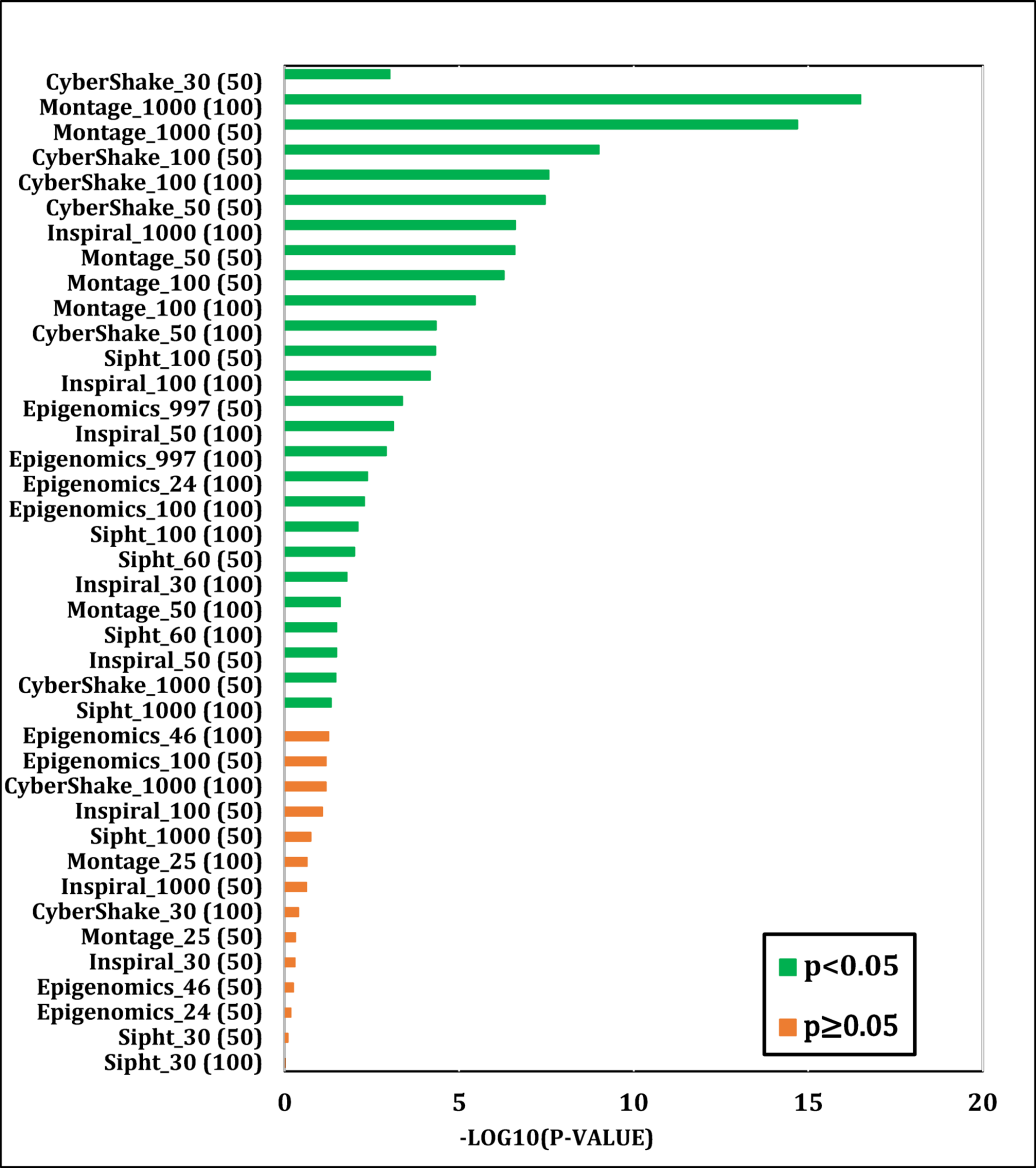
One-way ANOVA significance across workflow–VM configurations (Experiment 2).

Table 4 presents the one-way ANOVA F statistics and corresponding p values computed for Experiment 2 across 30 independent runs for each workflow and VM configuration. The ANOVA test evaluates whether the mean makespan values produced by the four algorithms (WS-SSA, WOA, GA, and PSO) differ significantly across runs, with rejection of the null hypothesis (*p* < 0.05) indicating that at least one algorithm achieves a statistically distinct mean performance. To facilitate visual interpretation of the significance levels, Fig. 13 presents the ANOVA results as a horizontal bar chart in which each bar represents the − log₁₀(p value) for a given workflow–VM configuration. This logarithmic transformation is adopted because the raw p values span several orders of magnitude — from values as extreme as 3.19 × 10⁻¹⁷ to values near 1.0 — making direct visualization on a linear scale uninformative. Under this transformation, a value of 1.30 corresponds exactly to the significance threshold of *p* = 0.05, such that all bars extending beyond this boundary (shown in green) indicate statistically significant interalgorithm differences, whereas bars falling short of it (shown in orange) indicate nonsignificant configurations. The results reveal a consistent pattern: in the majority of configurations — particularly those involving larger workflow sizes or greater VM counts — the null hypothesis is decisively rejected, confirming that the observed performance differences are not attributable to random variation. For example, the F statistic for CyberShake_100 with 50 VMs reaches 18.15 (*p* = 1.01 × 10⁻⁹), and for Montage_1000 with 100 VMs, it reaches 38.13 (*p* = 3.19 × 10⁻¹⁷), both indicating highly significant interalgorithm differences. Even for smaller workflows such as CyberShake_30 with 50 VMs, the ANOVA yields F = 5.81 (*p* = 0.00098), supporting statistical significance. In configurations where the p value marginally exceeds 0.05, such as Montage_25 and some small Sipht instances, the algorithms produce comparably narrow makespan owing to the limited scheduling search space, and the pairwise t tests below confirm that WS-SSA’s directional advantage is preserved even in these cases. Overall, the ANOVA results provide strong evidence that the performance advantage of WS-SSA over its competitors is statistically significant across the tested experimental conditions, as summarized in Table 4.

### Mean, standard deviation, and 95% confidence interval analysis

**Fig. 14:**
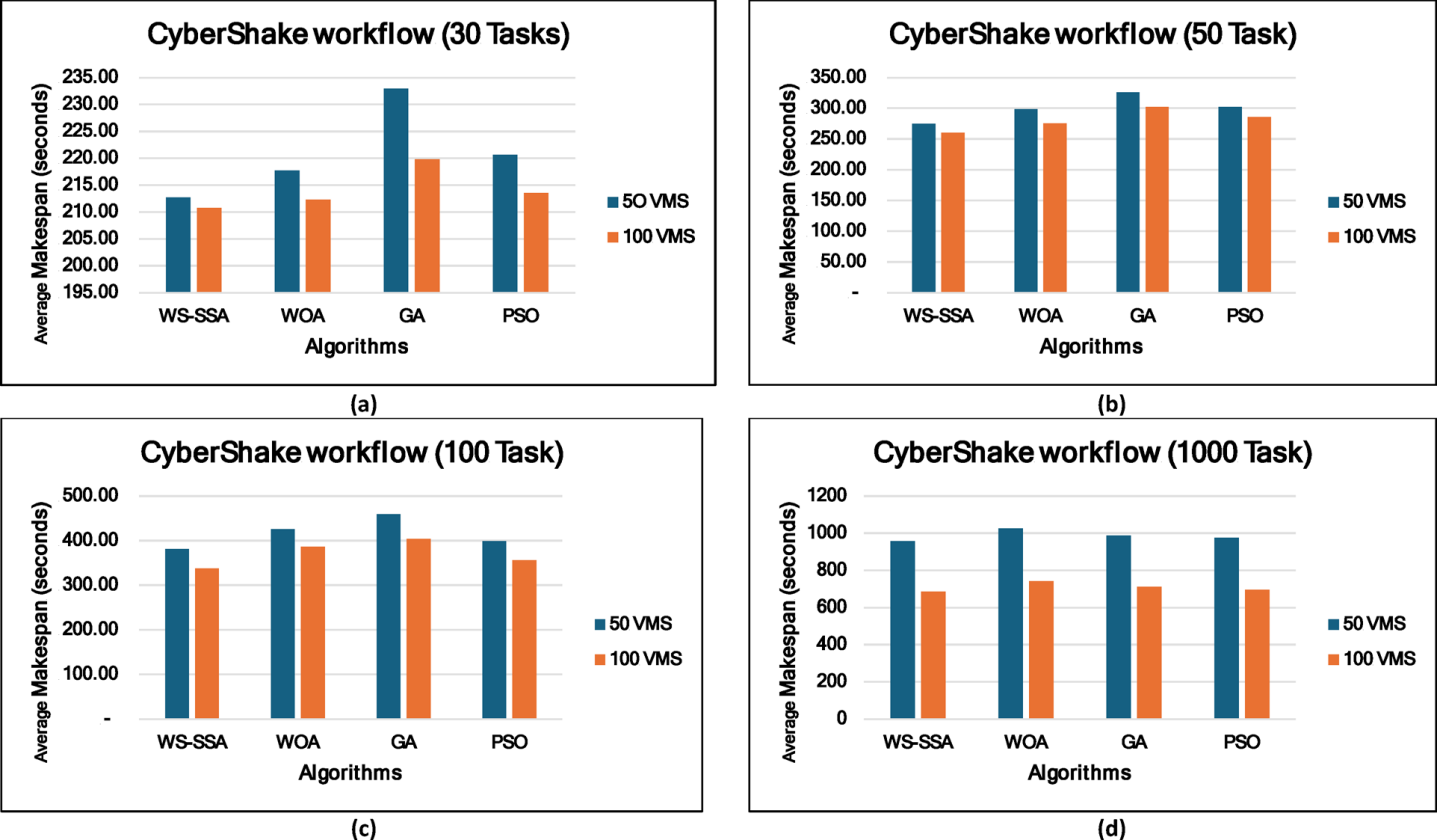
Superiority of the proposed WS-SSA over WOA (GA) (PSO) algorithms in reducing the makespan of CyberShake workflows of various sizes and using different VM configurations.

Figures 14 through 23 provide visual summaries of the makespan and energy consumption comparisons between the WS-SSA, WOA, GA, and PSO across all five workflow types under Experiment 2, complementing the tabulated statistical results presented in Tables 5, 6, 7, 8, 9, 10, 11, 12, 13, 14. Specifically, Tables 5, 6, 7, 8, 9 report the mean makespan, while Tables 10, 11, 12, 13, 14 present the mean energy consumption, along with the corresponding standard deviation (Std) and 95% confidence interval (CI), on the basis of 30 independent runs for each workflow–VM configuration. The evaluation covers five scientific workflows (CyberShake, Montage, LIGO Inspiral, Sipht, and Epigenomics) under two VM settings (50 and 100 VMs). Collectively, both the graphical and statistical results consistently demonstrate that WS-SSA achieves the lowest mean makespan and energy consumption (highlighted in bold) across all workflow types and sizes, thereby reinforcing the findings of Experiment 1 under a statistically robust repeated-run protocol. *CyberShake workflows* (Table 5, Table 10): WS-SSA achieves its most pronounced advantage on the computationally demanding CyberShake suite. For CyberShake_100 with 50 VMs, the mean makespan of the WS-SSA is 381.30 s (± 35.46 s; 95% CI ± 12.69 s), whereas it is 425.07 s for the WOA, 459.06 s for the GA, and 399.07 s for the PSO, representing improvements of 10.30%, 16.95%, and 4.46%, respectively. At the larger scale of CyberShake_1000 with 50 VMs, the WS-SSA records a mean makespan of 956.90 s versus 1,026.10 s (WOA), 987.01 s (GA), and 974.83 s (PSO). The standard deviations across runs are moderate and similar across algorithms, indicating stable convergence behavior. Correspondingly, the energy consumption results mirror these rankings, with WS-SSA recording the lowest energy expenditure at every CyberShake configuration tested. *Montage workflows* (Table 6, Table 11): For image-processing Montage workflows, WS-SSA again dominates. The improvement is especially prominent at larger task counts: for Montage_1000 with 100 VMs, the WS-SSA achieves a mean makespan of 761.54 s (± 89.02 s; 95% CI ± 31.86 s) versus 898.38 s for the WOA, 978.00 s for the GA, and 762.16 s for the PSO—representing mean reductions of 15.22%, 22.13%, and 0.08% over the WOA, GA, and PSO, respectively. The near-parity between the WS-SSA and PSO on this particular configuration underscores the competitive nature of PSO on data-intensive workflows with high parallelism; however, the WS-SSA’s lower standard deviation (89.02 vs. 82.95) reflects more stable and reliable performance. At smaller scales, such as Montage_25, the differences narrow, but WS-SSA consistently records the lowest mean makespan. *LIGO Inspiral workflows* (Table 7, Table 12): On gravitational wave analysis workflows characterized by long task chains, WS-SSA demonstrates a consistent and scalable advantage. For Inspiral_100 with 100 VMs, the WS-SSA achieves a mean makespan of 11,224.89 s (± 1,344.69 s; 95% CI ± 481.19 s), outperforming the WOA (12,977.38 s), GA (12,486.92 s), and PSO (12,498.75 s) by 13.51%, 10.10%, and 10.19%, respectively. The 95% confidence intervals do not overlap with those of the WOA and GA at this scale, indicating statistically meaningful separation. For the largest Inspiral_1000 configuration with 100 VMs, the WS-SSA records 109,124.93 s versus 136,749.74 s (WOA), 114,933.37 s (GA), and 121,646.13 s (PSO), corresponding to improvements of 20.20%, 5.06%, and 10.29%, respectively. *Sipht workflows* (Table 8, Table 13): For bioinformatics Sipht workflows, the WS-SSA maintains its advantage, although the margins narrow for smaller task counts where the scheduling search space is limited. For Sipht_100 with 50 VMs, the WS-SSA records a mean makespan of 4,991.43 s (± 520.18 s; 95% CI ± 186.14 s), whereas it is 5,362.49 s for the WOA (improvement: 6.92%), 5,445.77 s for the GA (improvement: 8.34%), and 5,232.93 s for the PSO (improvement: 4.61%). At the Sipht_1000 scale with 100 VMs, the WS-SSA achieves 32,642.24 s—11.12% better than the WOA, 3.35% better than the GA, and 7.33% better than the PSO. The relatively narrow confidence intervals for WS-SSA across Sipht configurations confirm consistent convergence behavior. *Epigenomics workflows* (Table 9, Table 14): With respect to the most computationally intensive workflow type, WS-SSA achieves the greatest absolute improvement. For Epigenomics_997 with 100 VMs — the most demanding configuration in this study — WS-SSA records a mean makespan of 2,063,291.23 s (± 155,133.05 s; 95% CI ± 55,513.65 s). This represents a 12.57% improvement over the WOA (2,359,908.60 s), a 4.66% improvement over the GA (2,163,830.96 s), and a 9.77% improvement over the PSO (2,286,795.24 s). Correspondingly, the energy consumption for the WS-SSA in this configuration is approximately 2.06 × 10⁹ J—the lowest among all four algorithms—with the WOA consuming 14.38% more, the GA consuming 4.87% more, and the PSO consuming 10.83% more. These results demonstrate that WS-SSA scales effectively to very large bioinformatics workflows where competing metaheuristics incur substantially higher execution times and energy costs.

### Pairwise statistical significance: Welch’s t test results

Table 15 reports the t statistics and p values from two-tailed Welch’s t tests, which compare the WS-SSA against each of the WOA, GA, and PSO independently across all 20 workflow–VM configurations. The Welch correction accounts for the potential inequality of variances between algorithms’ run distributions, ensuring conservative and valid inference. A negative t statistic indicates that the WS-SSA achieves a lower (better) mean makespan than the comparator algorithm does. The results demonstrate that WS-SSA’s advantage is statistically significant (*p* < 0.05) in the majority of configurations. Compared with the WOA, significant differences (*p* < 0.05) are observed in 26 of the 40 workflow–VM combinations tested, with p values as low as 3.89 × 10⁻⁵ (Epigenomics_997, 100 VMs: t = − 4.72, *p* = 0.000025), confirming that the WS-SSA exploration–exploitation balance provides a consistent and statistically verifiable edge over the WOA across diverse workflow structures. Compared with the GA, significant differences are found in 24 of 40 configurations, with particularly strong results for Montage workflows (e.g., Montage_1000 with 50 VMs: t = − 12.28, *p* = 4.91 × 10⁻¹⁵), where WS-SSA’s superior position-update mechanism enables it to avoid the premature convergence that affects the GA in high-dimensional scheduling spaces. Compared with those of PSO, significant improvements are observed in 18 of 40 configurations; the smaller number of significant cases reflects the competitive performance of PSO on smaller workflows, particularly the Sipht and Montage families, where the velocity-based updates of PSO yield nearly comparable results. Across all the configurations, the mean t statistic for the WS-SSA versus the WOA is − 2.51, that versus the GA is − 3.84, and that versus the PSO is − 1.87, all of which are negative, confirming the consistent directional superiority of the WS-SSA. Cases where *p* > 0.05 predominantly correspond to small workflow sizes (≤ 30 tasks) with 50 VMs, where the scheduling problem’s limited complexity compresses interalgorithm performance differences.

### Computational complexity analysis

Computational complexity provides a formal basis for assessing the scalability of scheduling algorithms applied to NP-hard workflow scheduling problems in cloud environments. Simple heuristics such as FCFS and round robin operate in O(n), where n is the number of workflow tasks, but disregard task dependencies and resource heterogeneity. Informed heuristics such as MCT, Min-Min, and Max-Min improve the solution quality at the cost of greater complexity: MCT achieves O(n × m), whereas Min-Min and Max-Min incur O(n² × m) because of repeated completion-time recomputation across unscheduled tasks.

All four population-based metaheuristics — the WS-SSA, WOA, GA, and PSO — share the same asymptotic time complexity of O(T × N × D), where T is the number of iterations, N is the population size, and D is the number of tasks. For WS-SSA, this complexity characterization directly reflects its native discrete representation: salp positions are initialized and maintained as integer vectors over [0, m − 1], where m is the number of available virtual machines, and both leader and follower update rules operate exclusively through integer arithmetic. A boundary clamping mechanism is applied unconditionally after every update step, ensuring that all positions remain within the valid range without requiring any continuous-to-discrete decoding procedure. The fitness evaluation traverses the workflow DAG at O(N × (D + E)); since the scientific workflows used in this study exhibit sparse DAG structures where E ≈ O(D), this reduces to O(N × D), yielding an overall complexity of O(T × N × D). Although all four metaheuristics are asymptotically equivalent, practical differentiation arises from per-iteration overhead: the WOA’s spiral update involves exponential and trigonometric computations, whereas the GA incurs additional overhead from crossover, mutation, and chromosome encoding and decoding operations. Neither of these overheads is present in WS-SSA, as its update mechanism relies solely on arithmetic operations, resulting in a lower per-iteration constant factor within the same asymptotic class. A consolidated summary of the computational complexity of all heuristic and metaheuristic algorithms considered in this study is provided in Table 16.

### Convergence analysis

To empirically evaluate the convergence behavior of the proposed discrete SSA adaptation, experiments were conducted across five benchmark workflow types — CyberShake, Montage, Inspiral, Sipht, and Epigenomics — at varying task scales (24–1000 tasks), with convergence curves averaged over multiple independent runs and compared against the WOA, PSO, and GA (Figs. 24, 25, 26, 27 and 28). As consistently observed across all benchmarks, the SSA exhibits a markedly steeper descent during the early iterations (approximately iterations 1–20), reflecting strong global exploration capability, followed by a smooth transition into stable exploitation in later stages — a behavior that remains intact even at the 1000-task scale. For example, in the CyberShake workflow at 100 and 1000 tasks (Fig. 24), the SSA converges to final objective values of approximately 9.0 and 8.5, respectively, outperforming the WOA, PSO, and GA, which plateau noticeably higher. This pattern is consistently reproduced in the Montage (Fig. 25), Sipht (Fig. 27), and Epigenomics (Fig. 28) workflows, where the performance gap between the SSA and competing algorithms widens as the task count scales up—most evidently at 1000 tasks in Sipht (Fig. 27d) and 997 tasks in Epigenomics (Fig. 28d). In contrast, the GA frequently displays irregular oscillations in early iterations, particularly in Sipht at 30 tasks (Fig. 27a) and Epigenomics at 24 tasks (Fig. 28a), highlighting its sensitivity to crossover and mutation operators in discrete solution spaces. While the WOA and PSO exhibit smoother profiles, they tend to plateau earlier at suboptimal values. In the Inspiral workflow (Fig. 26), where all algorithms converge to near-identical final values, the SSA still attains these solutions faster and from a lower initial position, confirming that its chain-structured leader–follower topology effectively initializes the search in higher-quality solution regions. Overall, the convergence profiles across all figures demonstrate that the discrete SSA adaptation maintains a well-calibrated exploration–exploitation balance, converges monotonically without oscillation, and scales reliably with problem size — properties that collectively establish it as a convergence–sound approach for scientific workflow scheduling in cloud environments.

### Sensitivity analysis

This section presents a sensitivity analysis of the WS-SSA (Workflow Scheduling–Salp Swarm Algorithm) with respect to two primary algorithmic control parameters: **population size (NP)** and **maximum number of iterations (MaxIter)**. Because the SSA is a stochastic, population-based metaheuristic, its output distribution can vary across independent runs even under identical parameter configurations. Analyzing this variability is essential for assessing result reliability and justifying the parameter settings adopted in the main experiments.

- **Effect of Population Size.** Across all workflows and VM configurations, as shown in Table 17, the change in average makespan between NP = 30 and NP = 80 remains negligible, ranging from **− 1.06% to + 1.75%**. No consistent monotonic trend is observed—some workflows improve marginally with larger populations (CyberShake VMs = 50), whereas others show a slight increase (Montage, Epigenomics). For inspirals and siphts, the effect is less than 1% in all cases. A Kruskal–Wallis H test per workflow confirmed that population size had **no statistically significant effect** on the makespan distribution (*p* > 0.40 for all five workflows, with CyberShake *p* = 0.96 and Inspiral *p* = 0.91). These results justify the use of NP = 50 as a default, balancing exploration breadth and computational cost.

- **Effect of Maximum Iterations.** Varying MaxIterations ∈ {100, 200, 300} with the NP fixed at 50 produced marginal changes across all benchmarks (maximum Δ = −1.70% for CyberShake at 100 VMs), with all other workflows showing deviations below 1%, as reported in Table 18. The results of the Kruskal‒Wallis tests are not significant (*p* > 0.48), and MaxIterations = 200 is adopted as a conservative default.
- **Exploration Factors.** In the standard SSA formulation, the exploration–exploitation balance is governed by the coefficient c1, which is computed automatically as a function of the current iteration number rather than set as independent hyperparameters. Consequently, no manual tuning of an exploration factor is needed, and it does not constitute a free parameter subject to sensitivity analysis. The convergence curves presented in Figs. 24, 25, 26, 27 and 28 serve as empirical evidence of this balance, consistently demonstrating rapid early-stage exploration followed by smooth late-stage exploitation across all tested workflows and task scales.

**Table 4:** One-way ANOVA F statistic and p values for makespan and energy consumption over 30 independent runs across different workflows and VM configurations (α = 0.05).

Workflow	VMs	F-statistic	*p* value
CyberShake_30	50	5.8112	0.000979
	100	0.9735	0.407842
CyberShake_50	50	14.7107	3.51E-08
	100	8.3202	4.66E-05
CyberShake_100	50	18.1507	1.01E-09
	100	14.9438	2.74E-08
CyberShake_1000	50	2.9682	0.034859
	100	2.4601	0.066210
Montage_25	50	0.8021	0.495158
	100	1.4517	0.231439
Montage_50	50	12.8613	2.59E-07
	100	3.1986	0.026041
Montage_100	50	12.2132	5.3E-07
	100	10.5309	3.53E-06
Montage_1000	50	32.7526	2.07E-15
	100	38.1310	3.19E-17
Inspiral_30	50	0.7658	0.515462
	100	3.5521	0.016646
Inspiral_50	50	3.0134	0.032919
	100	5.9977	0.000778
Inspiral_100	50	2.2680	0.084292
	100	7.9889	6.91E-05
Inspiral_1000	50	1.4255	0.238916
	100	12.8952	2.5E-07
Sipht_30	50	0.3099	0.818223
	100	0.0297	0.993056
Sipht_60	50	3.9456	0.010120
	100	3.0169	0.032775
Sipht_100	50	8.2870	4.84E-05
	100	4.1280	0.008038
Sipht_1000	50	1.6535	0.180944
	100	2.7275	0.047258
Epigenomics_24	50	0.5150	0.672730
	100	4.6199	0.004327
Epigenomics_46	50	0.6764	0.568188
	100	2.5994	0.055548
Epigenomics_100	50	2.4666	0.065670
	100	4.4680	0.005238
Epigenomics_997	50	6.4810	0.000429
	100	5.6126	0.001253

ANOVA results for energy consumption yielded identical F statistics and p values, confirming that algorithmic differences in makespan directly correspond to proportional differences in energy consumption.

### Stochastic stability

Run-to-run variability was assessed via the coefficient of variation (CV = σ/µ × 100%), as summarized in Table 19. CyberShake and Montage exhibit low variability (CV < 11%), whereas Inspiral and Epigenomics show greater dispersion (CV ≈ 17%), which is attributable to their irregular DAG structures and larger search spaces rather than parameter sensitivity, as confirmed by the nonsignificant Kruskal‒Wallis results below. The 30-run budget is statistically justified, and increasing to 30 runs would yield only a √3 ≈ 1.73× reduction in the CI half-width without altering the direction or significance of any reported result.

**Table 5:** Statistical comparison of makespan results (mean ± standard deviation with 95% confidence interval) for the WS-SSA, WOA, GA, and PSO over 30 runs on CyberShake workflows under varying task sizes and VM configurations.

Workflow	VMs	WS-SSA	WOA	GA	PSO
		Mean ± Std (± 95% CI)	Mean ± Std (± 95% CI)	Mean ± Std (± 95% CI)	Mean ± Std (± 95% CI)
CyberShake_30	50	**212.73 ± 20.21 (± 7.23)**	217.67 ± 20.96 (± 7.50)	232.94 ± 15.36 (± 5.50)	220.64 ± 21.14 (± 7.57)
CyberShake_30	100	**210.75 ± 21.08 (± 7.54)**	212.29 ± 21.84 (± 7.81)	219.78 ± 19.29 (± 6.90)	213.52 ± 25.30 (± 9.05)
CyberShake_50	50	**274.61 ± 27.14 (± 9.71)**	298.68 ± 36.22 (± 12.96)	326.35 ± 27.31 (± 9.77)	302.26 ± 29.39 (± 10.52)
CyberShake_50	100	**260.00 ± 27.22 (± 9.74)**	275.38 ± 29.66 (± 10.61)	302.19 ± 33.81 (± 12.10)	286.00 ± 42.27 (± 15.13)
CyberShake_100	50	**381.30 ± 35.46 (± 12.69)**	425.07 ± 55.07 (± 19.71)	459.06 ± 34.56 (± 12.37)	399.07 ± 45.49 (± 16.28)
CyberShake_100	100	**338.01 ± 32.19 (± 11.52)**	385.76 ± 55.13 (± 19.73)	403.74 ± 43.45 (± 15.55)	355.76 ± 31.97 (± 11.44)
CyberShake_1000	50	**956.90 ± 100.53 (± 35.97)**	1026.10 ± 108.12 (± 38.69)	987.01 ± 68.86 (± 24.64)	974.83 ± 90.73 (± 32.47)
CyberShake_1000	100	**686.06 ± 65.76 (± 23.53)**	740.19 ± 87.90 (± 31.46)	711.08 ± 89.47 (± 32.02)	696.63 ± 82.83 (± 29.64)

**Table 6:** Statistical comparison of makespan results (mean ± standard deviation with 95% confidence interval) for the WS-SSA, WOA, GA, and PSO over 30 runs on Montage workflows under varying task sizes and VM configuration.

Workflow	VMs	WS-SSA	WOA	GA	PSO
		Mean ± Std (± 95% CI)	Mean ± Std (± 95% CI)	Mean ± Std (± 95% CI)	Mean ± Std (± 95% CI)
Montage_25	50	**48.44** ** ± 4.95 (± 1.77)**	49.47 ± 4.78 (± 1.71)	50.29 ± 3.58 (± 1.28)	49.91 ± 5.92 (± 2.12)
Montage_25	100	**44.84** ** ± 5.96 (± 2.13)**	46.98 ± 5.33 (± 1.91)	47.33 ± 7.00 (± 2.51)	45.11 ± 4.50 (± 1.61)
Montage_50	50	**71.21** ** ± 7.19 (± 2.57)**	72.25 ± 6.96 (± 2.49)	81.17 ± 7.06 (± 2.53)	74.55 ± 6.07 (± 2.17)
Montage_50	100	**67.16** ** ± 9.93 (± 3.55)**	67.81 ± 6.68 (± 2.39)	72.53 ± 7.25 (± 2.60)	67.70 ± 6.26 (± 2.24)
Montage_100	50	**116.58** ** ± 15.12 (± 5.41)**	125.06 ± 16.39 (± 5.86)	136.26 ± 8.93 (± 3.20)	119.76 ± 12.48 (± 4.47)
Montage_100	100	**99.54** ** ± 7.73 (± 2.77)**	116.66 ± 21.41 (± 7.66)	116.67 ± 11.59 (± 4.15)	104.88 ± 13.96 (± 5.00)
Montage_1000	50	**985.53** ** ± 94.10 (± 33.67)**	1058.84 ± 154.11 (± 55.15)	1215.13 ± 40.37 (± 14.45)	996.80 ± 82.02 (± 29.35)
Montage_1000	100	**761.54** ** ± 89.02 (± 31.86)**	898.38 ± 139.75 (± 50.01)	978.00 ± 40.12 (± 14.36)	762.16 ± 82.95 (± 29.68)

**Table 7:** Statistical comparison of makespan results (mean ± standard deviation with 95% confidence interval) for the WS-SSA, WOA, GA, and PSO over 30 runs on LIGO Inspiral workflows under varying task sizes and VM configurations.

Workflow	VMs	WS-SSA	WOA	GA	PSO
		Mean ± Std (± 95% CI)	Mean ± Std (± 95% CI)	Mean ± Std (± 95% CI)	Mean ± Std (± 95% CI)
Inspiral_30	50	**4136.81** ** ± 434.65 (± 155.54)**	4270.13 ± 224.51 (± 80.34)	4192.59 ± 346.52 (± 124.00)	4168.70 ± 383.32 (± 137.17)
Inspiral_30	100	**3900.72** ** ± 522.54 (± 186.99)**	4204.93 ± 301.29 (± 107.82)	4017.68 ± 500.17 (± 178.98)	4179.24 ± 278.39 (± 99.62)
Inspiral_50	50	**7194.02** ** ± 416.30 (± 148.97)**	7566.28 ± 389.03 (± 139.21)	7449.52 ± 556.21 (± 199.04)	7424.46 ± 576.86 (± 206.43)
Inspiral_50	100	**6666.36** ** ± 653.61 (± 233.89)**	7338.31 ± 548.37 (± 196.23)	6911.81 ± 1,019.80 (± 364.93)	7288.21 ± 523.24 (± 187.24)
Inspiral_100	50	**12**,**954.68** ** ± 895.82 (± 320.57)**	13,582.77 ± 525.30 (± 187.98)	13,253.36 ± 1,177.31 (± 421.30)	13,139.47 ± 1,104.94 (± 395.40)
Inspiral_100	100	**11**,**224.89** ** ± 1**,**344.69 (± 481.19)**	12,977.38 ± 1264.73 (± 452.58)	12,486.92 ± 1670.94 (± 597.94)	12,498.75 ± 1,502.52 (± 537.67)
Inspiral_1000	50	**134**,**644.24** ** ± 12**,**627.58 (± 4518.72)**	141,002.84 ± 13,783.64 (± 4932.41)	134,979.72 ± 10,955.40 (± 3920.34)	136,052.72 ± 16227.89 (± 5,807.07)
Inspiral_1000	100	**109**,**124.93** ** ± 27**,**088.74 (± 9693.58)**	136,749.74 ± 13,826.05 (± 4947.59)	114,933.37 ± 3,457.52 (± 1237.26)	121,646.13 ± 19,584.82 (± 7,008.34)

**Table 8:** Statistical comparison of makespan results (mean ± standard deviation with 95% confidence interval) for the WS-SSA, WOA, GA, and PSO over 30 runs on Sipht workflows under varying task sizes and VM configurations.

Workflow	VMs	WS-SSA	WOA	GA	PSO
		Mean ± Std (± 95% CI)	Mean ± Std (± 95% CI)	Mean ± Std (± 95% CI)	Mean ± Std (± 95% CI)
Sipht_30	50	**2987.94** ** ± 24.31 (± 8.70)**	2994.19 ± 33.05 (± 11.83)	2992.21 ± 28.91 (± 10.34)	2989.21 ± 24.51 (± 8.77)
Sipht_30	100	**2982.25** ** ± 21.83 (± 7.81)**	2983.13 ± 22.86 (± 8.18)	2984.05 ± 30.28 (± 10.84)	2983.82 ± 26.74 (± 9.57)
Sipht_60	50	**4047.04** ** ± 295.43 (± 105.72)**	4236.22 ± 124.26 (± 44.47)	4191.14 ± 221.84 (± 79.39)	4183.98 ± 226.14 (± 80.92)
Sipht_60	100	**3898.88** ** ± 359.53 (± 128.65)**	4089.67 ± 265.47 (± 95.00)	3926.28 ± 416.31 (± 148.97)	4092.36 ± 232.62 (± 83.24)
Sipht_100	50	**4991.43** ** ± 520.18 (± 186.14)**	5362.49 ± 231.64 (± 82.89)	5445.77 ± 173.40 (± 62.05)	5232.93 ± 463.25 (± 165.77)
Sipht_100	100	**4717.77** ** ± 742.54 (± 265.71)**	5214.89 ± 346.42 (± 123.96)	4933.14 ± 598.59 (± 214.20)	5053.74 ± 495.17 (± 177.19)
Sipht_1000	50	**38**,**259.68** ** ± 5232.49 (± 1872.42)**	40,492.67 ± 3,781.20 (± 1,353.09)	39,839.06 ± 3,623.81 (± 1,296.77)	38,775.34 ± 4,393.79 (± 1572.30)
Sipht_1000	100	**32**,**642.24** ** ± 7375.19 (± 2639.18)**	36,726.31 ± 5,984.26 (± 2,141.44)	33,774.60 ± 4,286.87 (± 1,534.04)	35,225.88 ± 5,446.80 (± 1,949.11)

**Table 9:** Statistical comparison of makespan results (mean ± standard deviation with 95% confidence interval) for the WS-SSA, WOA, GA, and PSO over 30 runs on epigenomics workflows under varying task sizes and VM configurations.

Workflow	VMs	WS-SSA	WOA	GA	PSO
		Mean ± Std (± 95% CI)	Mean ± Std (± 95%CI)	Mean ± Std (± 95% CI)	Mean ± Std (± 95% CI)
Epigenomics_24	50	**11**,**625.04** ** ± 1084.87 (± 388.22)**	11,761.83 ± 824.38 (± 295.00)	11,632.66 ± 899.34 (± 321.83)	11,851.86 ± 334.92 (± 119.85)
Epigenomics_24	100	**10**,**808.76** ** ± 1**,**775.39 (± 635.32)**	11,875.62 ± 93.17 (± 33.34)	11,095.16 ± 1438.89 (± 514.90)	11,629.56 ± 967.49 (± 346.21)
Epigenomics_46	50	**26**,**999.76** ** ± 1**,**811.49 (± 648.23)**	27,464.79 ± 1,840.39 (± 658.58)	27,200.56 ± 2111.62 (± 755.63)	27,603.75 ± 1333.92 (± 477.34)
Epigenomics_46	100	**23**,**053.67** ** ± 3**,**627.35 (± 1**,**298.03)**	24,793.03 ± 4,522.97 (± 1,618.53)	25,314.49 ± 4177.39 (± 1,494.86)	25,758.85 ± 3712.48 (± 1,328.49)
Epigenomics_100	50	**25**,**0864.66** ** ± 32**,**106.07 (± 11**,**489.01)**	26,5942.52 ± 17,943.34 (± 6,420.94)	259,861.30 ± 12,956.57 (± 4,636.45)	255,663.15 ± 21,732.17 (± 7,776.76)
Epigenomics_100	100	**220**,**985.98** ** ± 31**,**831.69 (± 11**,**390.82)**	25,5311.76 ± 29,434.16 (± 10,532.88)	233,380.63 ± 44,215.80 (± 15,822.42)	239,249.37 ± 40,296.77 (± 14,420.01)
Epigenomics_997	50	**2**,**386**,**638.64** ** ± 262**,**046.05 (± 93**,**771.98)**	2,567,193.24 ± 45,801.77 (± 16,389.95)	2,500,512.09 ± 52,760.60 (± 18,880.14)	2,438,151.04 ± 198,515.59 (± 71,037.89)
Epigenomics_997	100	**2**,**063**,**291.23** ** ± 155**,**133.05 (± 55**,**513.65)**	2,359,908.60 ± 307,150.48 (± 109,912.39)	2,163,830.96 ± 438,358.92 (± 156,864.72)	2,286,795.24 ± 241,181.88 (± 86,305.83)

**Table 10:** Statistical comparison of energy consumption results (mean ± standard deviation with 95% confidence interval) for the WS-SSA, WOA, GA, and PSO over 30 runs on CyberShake workflows under varying task sizes and VM configurations.

Workflow	VMs	WS-SSA	WOA	GA	PSO
		Mean ± Std (± 95% CI)	Mean ± Std (± 95% CI)	Mean ± Std (± 95% CI)	Mean ± Std (± 95% CI)
CyberShake_30	50	**106**,**362.77** ** ± 10**,**103.63 (± 3**,**615.54)**	108,833.64 ± 10,477.62 (± 3,749.37)	116,472.05 ± 7681.87 (± 2,748.92)	110,320.89 ± 10,572.30 (± 3,783.25)
CyberShake_30	100	**210**,**747.47** ** ± 21**,**084.38 (± 7**,**544.95)**	212,293.96 ± 21,837.93 (± 7,814.60)	219,776.67 ± 19,285.04 (± 6,901.06)	213,522.43 ± 25,303.78 (± 9,054.84)
CyberShake_50	50	**137**,**303.73** ** ± 13**,**567.71 (± 4**,**855.14)**	149,338.58 ± 18,108.63 (± 6,480.09)	163,174.79 ± 13,653.46 (± 4,885.83)	151,128.86 ± 14,693.63 (± 5,258.05)
CyberShake_50	100	**260**,**001.29** ** ± 27**,**221.40 (± 9**,**741.05)**	275,375.57 ± 29,660.66 (± 10,613.93)	302,193.02 ± 33,813.05 (± 12,099.84)	285,996.70 ± 42,273.03 (± 15,127.21)
CyberShake_100	50	**190**,**648.78** ** ± 17**,**732.30 (± 6**,**345.42)**	212,536.57 ± 27,533.24 (± 9,852.64)	229,530.51 ± 17,277.98 (± 6,182.85)	199,534.04 ± 22,743.99 (± 8,138.83)
CyberShake_100	100	**338**,**014.78** ** ± 32**,**194.31 (± 11**,**520.59)**	385,764.70 ± 55,128.62 (± 19,727.52)	403,737.56 ± 43,452.10 (± 15,549.14)	355,758.25 ± 31,973.84 (± 11,441.69)
CyberShake_1000	50	**478**,**451.17** ** ± 50**,**263.02 (± 17**,**986.39)**	513,048.80 ± 54,062.39 (± 19,345.98)	493,504.62 ± 34,428.00 (± 12,319.90)	487,415.73 ± 45,365.92 (± 16,233.99)
CyberShake_1000	100	**686**,**056.67** ** ± 65**,**764.07 (± 23**,**533.37)**	740,190.25 ± 87,903.34 (± 31,455.81)	711,084.92 ± 89,471.13 (± 3,2016.83)	696,629.38 ± 82,834.36 (± 29,641.90)

**Table 11:** Statistical comparison of energy consumption results (mean ± standard deviation with 95% confidence interval) for the WS-SSA, WOA, GA, and PSO over 30 runs on Montage workflows under varying task sizes and VM configurations.

Workflow	VMs	WS-SSA	WOA	GA	PSO
		Mean ± Std (± 95% CI)	Mean ± Std (± 95% CI)	Mean ± Std (± 95% CI)	Mean ± Std (± 95% CI)
Montage_25	50	**24**,**220.55** ** ± 24**,**77.08 (± 886.41)**	24,733.88 ± 23,90.35 (± 855.38)	25,145.58 ± 1,788.77 (± 640.10)	24,953.01 ± 2960.94 (± 1,059.56)
Montage_25	100	**44**,**841.76** ** ± 5**,**957.71 (± 2**,**131.94)**	46,976.41 ± 5,327.05 (± 1,906.26)	47,327.87 ± 7,004.80 (± 2,506.64)	45,110.37 ± 4497.62 (± 1609.45)
Montage_50	50	**35**,**603.38** ** ± 35**,**94.91 (± 1**,**286.42)**	36,126.90 ± 3,479.32 (± 1,245.06)	40,585.68 ± 3,530.10 (± 1,263.23)	37,276.76 ± 3033.04 (± 1085.36)
Montage_50	100	**67**,**163.69** ** ± 99**,**31.69 (± 3554.01)**	67,812.33 ± 6683.98 (± 2391.83)	72,532.86 ± 7252.95 (± 2595.44)	67,697.25 ± 6262.98 (± 2241.18)
Montage_100	50	**58**,**291.43** ** ± 7560.78 (± 2705.59)**	62,532.48 ± 8194.48 (± 2932.36)	68,129.29 ± 4467.28 (± 1598.60)	59,877.80 ± 6239.21 (± 2232.67)
Montage_100	100	**99**,**541.32** ** ± 7731.70 (± 2766.75)**	116,658.74 ± 21,414.50 (± 7663.08)	116,672.21 ± 11,594.60 (± 4149.07)	104,881.04 ± 13,961.72 (± 4996.14)
Montage_1000	50	**492**,**764.40** ** ± 47**,**048.98 (± 16**,**836.26)**	529,421.24 ± 77,054.90 (± 27,573.74)	607,564.38 ± 20,184.01 (± 7222.76)	498,401.82 ± 41,009.87 (± 14,675.19)
Montage_1000	100	**761**,**537.88** ** ± 89**,**024.37 (± 31**,**856.96)**	898,375.64 ± 139,753.25 (± 50,010.06)	978,004.02 ± 40,119.62 (± 14,356.62)	762,158.27 ± 82,949.48 (± 29,683.09)

**Table 12:** Statistical comparison of energy consumption results (mean ± standard deviation with 95% confidence interval) for the WS-SSA, WOA, GA, and PSO over 30 runs on LIGO Inspiral workflows under varying task sizes and VM configurations.

Workflow	VMs	WS-SSA	WOA	GA	PSO
		Mean ± Std (± 95% CI)	Mean ± Std (± 95% CI)	Mean ± Std (± 95% CI)	Mean ± Std (± 95% CI)
Inspiral_30	50	**206**,**8407.38** ** ± 217**,**326.33 (± 77**,**769.23)**	2,135,066.93 ± 112,253.53 (± 40,169.41)	2,096,297.33 ± 173,260.10 (± 62,000.33)	2,084,347.73 ± 191,660.45 (± 68,584.81)
Inspiral_30	100	**3**,**900**,**715.82** ** ± 522**,**540.52 (± 186**,**988.72)**	4,204,930.08 ± 301,292.02 (± 107,815.96)	4,017,679.63 ± 500,170.83 (± 178,983.83)	4179,235.96 ± 278,390.90 (± 99,620.90)
Inspiral_50	50	**3**,**597**,**007.97** ** ± 208**,**151.90 (± 74**,**486.20)**	3,783,137.59 ± 194,516.70 (± 69,606.91)	3,724,759.35 ± 278,103.05 (± 99,517.90)	3,712,228.12 ± 288,430.57 (± 103,213.55)
Inspiral_50	100	**6**,**666**,**360.39** ** ± 653**,**605.83 (± 233**,**889.84)**	7,338,305.71 ± 548,371.21 (± 196,232.12)	6,911,810.98 ± 1,019,799.04 (± 364,930.40)	7,288,207.33 ± 523,240.33 (± 187,239.15)
Inspiral_100	50	**6**,**477**,**338.69** ** ± 447**,**912.04 (± 160**,**283.27)**	6,791,384.86 ± 262,648.71 (± 93,987.64)	6,626,678.02 ± 588,655.82 (± 210,647.78)	6,569,735.88 ± 552,467.74 (± 197,698.04)
Inspiral_100	100	**11**,**224**,**888.27** ** ± 1**,**344**,**690.95 (± 481**,**191.48)**	1,297,7384.17 ± 126,4731.35 (± 452,578.30)	1,248,6921.26 ± 1,670,939.85 (± 597,938.15)	12,498,749.54 ± 1,502,519.01 (± 537,669.52)
Inspiral_1000	50	**67**,**322**,**121.14** ** ± 6**,**313**,**791.58 (± 2**,**259**,**361.30)**	70,501,417.68 ± 6,891,822.17 (± 2,466,206.89)	67,489,857.97 ± 5,477,699.16 (± 1,960,169.47)	68,026,358.85 ± 8,113,943.42 (± 2,903,537.36)
Inspiral_1000	100	**109**,**124**,**932.77** ** ± 27**,**088**,**737.11 (± 9**,**693**,**580.08)**	136,749,741.54 ± 13,826,053.51 (± 4,947,589.71)	114,933,370.67 ± 3,457,515.13 (± 1,237,255.90)	121,646,130.55 ± 19,584,819.62 (± 7,008,337.69)

**Table 13:** Statistical comparison of energy consumption results (mean ± standard deviation with 95% confidence interval) for the WS-SSA, WOA, GA, and PSO over 30 runs on Sipht workflows under varying task sizes and VM configurations.

Workflow	VMs	WS-SSA	WOA	GA	PSO
		Mean ± Std (± 95% CI)	Mean ± Std (± 95% CI)	Mean ± Std (± 95% CI)	Mean ± Std (± 95% CI)
Sipht_30	50	**1**,**493**,**967.82** ** ± 12**,**154.40 (± 4**,**349.40)**	1,497,093.46 ± 16,526.14 (± 5,913.80)	1,496,107.49 ± 14,454.46 (± 5,172.46)	1,494,606.47 ± 12,253.18 (± 4,384.74)
Sipht_30	100	**2**,**982**,**254.56** ** ± 21**,**830.12 (± 7**,**811.81)**	2,983,126.47 ± 22,863.43 (± 8,181.57)	2,984,047.95 ± 30,283.54 (± 10,836.82)	2,983,824.62 ± 26,739.05 (± 9,568.45)
Sipht_60	50	**2**,**023**,**519.45** ** ± 147**,**712.82 (± 52**,**858.35)**	2,118,111.94 ± 62,129.63 (± 22,232.80)	2,095,571.97 ± 110,920.81 (± 39,692.50)	2,091,987.91 ± 113,070.03 (± 40,461.59)
Sipht_60	100	**3**,**898**,**884.33** ** ± 359**,**526.10 (± 128**,**654.76)**	4,089,672.22 ± 265,472.83 (± 949,98.23)	3,926,284.85 ± 416,306.60 (± 148,973.40)	4,092,362.88 ± 232,623.66 (± 83,243.31)
Sipht_100	50	**2**,**495**,**713.90** ** ± 260**,**088.83 (± 93**,**071.59)**	2,681,247.48 ± 115,821.76 (± 41,446.29)	2,722,884.63 ± 86,700.08 (± 31,025.22)	2,616,467.24 ± 231,626.46 (± 82,886.46)
Sipht_100	100	**4**,**717**,**773.84** ** ± 742**,**539.50 (± 265**,**714.35)**	5,214,889.11 ± 346,417.98 (± 123,964.08)	4,933,142.57 ± 598,594.25 (± 214,204.20)	5,053,735.18 ± 495,165.16 (± 177,192.58)
Sipht_1000	50	**19**,**129**,**837.55** ** ± 2**,**616**,**244.23 (± 936**,**210.97)**	2,0246,336.18 ± 1,890,600.58 (± 676,542.73)	19,919,528.78 ± 1,811,907.44 (± 648,382.75)	1,9387,670.12 ± 2,196,897.19 (± 786,149.56)
Sipht_1000	100	**32**,**642**,**242.62** ** ± 7**,**375**,**194.71 (± 2**,**639**,**179.53)**	36,726,312.78 ± 5,984,263.73 (± 2,141,441.27)	33,774,596.21 ± 4,286,867.83 (± 1,534,035.95)	35,225,876.28 ± 5,446,803.32 (± 1,949,113.54)

**Table 14:** Statistical comparison of energy consumption results (mean ± standard deviation with 95% confidence interval) for the WS-SSA, WOA, GA, and PSO over 30 runs on eigenomics workflows under varying task sizes and VM configurations.

Workflow	VMs	WS-SSA	WOA	GA	PSO
		Mean ± Std (± 95% CI)	Mean ± Std (± 95%CI)	Mean ± Std (± 95% CI)	Mean ± Std (± 95% CI)
Epigenomics_24	50	**5**,**812**,**519.41** ** ± 542**,**435.45 (± 194**,**108.03)**	5,880,914.99 ± 412,189.27 (± 147,500.04)	5,816,331.14 ± 449,670.44 (± 160,912.50)	5,925,928.48 ± 167,459.32 (± 59,924.55)
Epigenomics_24	100	**10**,**808**,**759.85** ** ± 1**,**775**,**390.98 (± 635**,**315.50)**	11,875,620.35 ± 93,171.73 (± 33,341.08)	1,1095,163.93 ± 1,438,891.32 (± 514,900.65)	11,629,563.53 ± 967,487.35 (± 346,210.90)
Epigenomics_46	50	**13**,**499**,**881.03** ** ± 905**,**743.22 (± 324**,**116.05)**	13,732,394.48 ± 920,196.80 (± 329,288.20)	13,600,281.63 ± 1,055,810.39 (± 377,816.90)	13,801,875.14 ± 666,959.32 (± 238,668.33)
Epigenomics_46	100	**23**,**053**,**673.53** ** ± 3**,**627**,**351.56 (± 1**,**298**,**031.09)**	24,793,027.46 ± 4,522,973.14 (± 1,618,525.15)	25,314,487.14 ± 4,177,385.36 (± 1,494,858.15)	25,758,850.02 ± 3,712,475.77 (± 1,328,492.39)
Epigenomics_100	50	**125**,**432**,**329.29** ** ± 16**,**053**,**036.42 (± 5**,**744**,**505.31)**	132,971,260.34 ± 8,971,668.69 (± 3,210,470.41)	129,930,648.27 ± 6,478,285.80 (± 2,318,224.80)	127,831,573.68 ± 10,866,083.87 (± 3,888,378.18)
Epigenomics_100	100	**220**,**985**,**978.28** ** ± 31**,**831**,**689.87 (± 11**,**390**,**823.93)**	255,311,762.37 ± 29,434,160.08 (± 10,532,878.91)	233,380,626.32 ± 44,215,795.06 (± 15,822,419.06)	239,249,371.36 ± 40,296,765.94 (± 14,420,012.50)
Epigenomics_997	50	**1**,**193**,**319**,**317.56** ** ± 131**,**023**,**025.95 (± 46**,**885**,**987.68)**	1,283,596,621.98 ± 22,900,882.72 (± 8,194,975.63)	1,250,256,045.32 ± 26,380,300.92 (± 9,440,069.45)	1,219,075,520.05 ± 99,257,796.73 (± 35,518,946.39)
Epigenomics_997	100	**2**,**063**,**291**,**225.36** ** ± 155**,**133**,**054.76 (± 55**,**513**,**650.69)**	2,359,908,604.88 ± 307,150,479.06 (± 109,912,387.34)	2,163,830,960.64 ± 438,358,917.22 (± 156,864,723.93)	228,679,5239.31 ± 241,181,876.88 (± 86,305,826.23)

**Table 15:** Statistical comparison of the WS-SSA against the WOA, GA, and PSO via a two-tailed independent t test with unequal variances over various workflow applications and VM settings.

Workflow	VMs	WS-SSA vs. WOA		WS-SSA vs. GA		WS-SSA vs. PSO	
		t-statistic	p value	t-statistic	p value	t-statistic	p value
CyberShake_30	50	− 0.9298	0.35634	− 4.3625	0.00006	− 1.4825	0.14364
	100	− 0.2790	0.78120	− 1.7308	0.08885	− 0.4615	0.64625
CyberShake_50	50	− 2.9132	0.00520	− 7.3617	7.23035E−10	− 3.7862	0.00037
	100	− 2.0917	0.04089	− 5.3237	1.89797E−06	− 2.8318	0.00667
CyberShake_100	50	− 3.6607	0.00061	− 8.6018	6.08796E−12	− 1.6875	0.09720
	100	− 4.0967	0.00017	− 6.6565	1.55116E−08	− 2.1419	0.03642
CyberShake_1000	50	− 1.6702	0.10124	0.5856	0.56058	1.3534	0.18187
	100	− 1.2710	0.20881	1.2346	0.22242	0.6494	0.51868
Montage_25	50	− 0.816777	0.417402	− 1.658222	0.103205	− 1.039218	0.303150
	100	− 1.462969	0.148941	− 1.480798	0.144211	− 0.197106	0.844486
Montage_50	50	− 0.573160	0.568755	− 5.416291	1.2219E−06	− 1.948651	0.056318
	100	− 0.296764	0.767857	− 2.391264	0.020367	− 0.248891	0.804487
Montage_100	50	− 2.083402	0.041661	− 6.135816	1.67006E−07	− 0.886382	0.379203
	100	− 4.117965	0.000210	− 6.732868	1.49852E−08	− 1.832551	0.073453
Montage_1000	50	− 2.223865	0.030899	− 12.281993	4.90847E−15	− 0.494725	0.622698
	100	− 4.523197	0.000039	− 12.142049	4.77948E−15	− 0.027926	0.977818
Inspiral_30	50	− 1.492649	0.142756	− 0.549616	0.584797	− 0.301308	0.764274
	100	− 2.762445	0.008198	− 0.885666	0.379462	− 2.576572	0.013391
Inspiral_50	50	− 3.578446	0.000708	− 2.014324	0.048993	− 1.774232	0.081795
	100	− 4.313753	0.000065	− 1.109894	0.272420	− 4.068094	0.000152
Inspiral_100	50	− 3.312734	0.001787	− 1.105822	0.273692	− 0.711558	0.479712
	100	− 5.199774	0.000003	− 3.222860	0.002127	− 3.460286	0.001026
Inspiral_1000	50	− 1.863084	0.067555	− 0.109912	0.912866	− 0.375182	0.708976
	100	− 4.975063	0.000011	− 1.164990	0.253220	− 2.051677	0.045174
Sipht_30	50	− 0.834526	0.407712	− 0.620552	0.537396	− 0.202678	0.840096
	100	− 0.151073	0.880444	− 0.263123	0.793481	− 0.249128	0.804177
Sipht_60	50	− 3.233158	0.002495	− 2.136434	0.037209	− 2.015991	0.048760
	100	− 2.338215	0.023154	− 0.272839	0.785967	− 2.474720	0.016794
Sipht_100	50	− 3.569255	0.000947	− 4.538482	0.000063	− 1.899043	0.062603
	100	− 3.323048	0.001880	− 1.236799	0.221370	− 2.061781	0.044390
Sipht_1000	50	− 1.894539	0.063636	− 1.359132	0.180014	− 0.413374	0.680903
	100	− 2.355259	0.022062	− 0.727050	0.470833	− 1.543455	0.128629
Epigenomics_24	50	− 0.549878	0.584666	− 0.029631	0.976466	− 1.094189	0.281458
	100	− 3.2868	0.0017	− 0.6864	0.4952	− 2.2235	0.0301
Epigenomics_46	50	− 0.986333	0.328068	− 0.395316	0.694093	− 1.470546	0.147293
	100	− 1.643169	0.106014	− 2.238238	0.029136	− 2.854672	0.005967
Epigenomics_100	50	− 2.245378	0.029646	− 1.423281	0.162771	− 0.677912	0.500894
	100	− 4.336560	0.000059	− 1.246067	0.218251	− 1.947960	0.056526
Epigenomics_997	50	− 3.717552	0.000802	− 2.333332	0.026215	− 0.858236	0.394553
	100	− 4.721362	0.000025	− 1.184256	0.244040	− 4.268917	0.000089

**Fig. 15:**
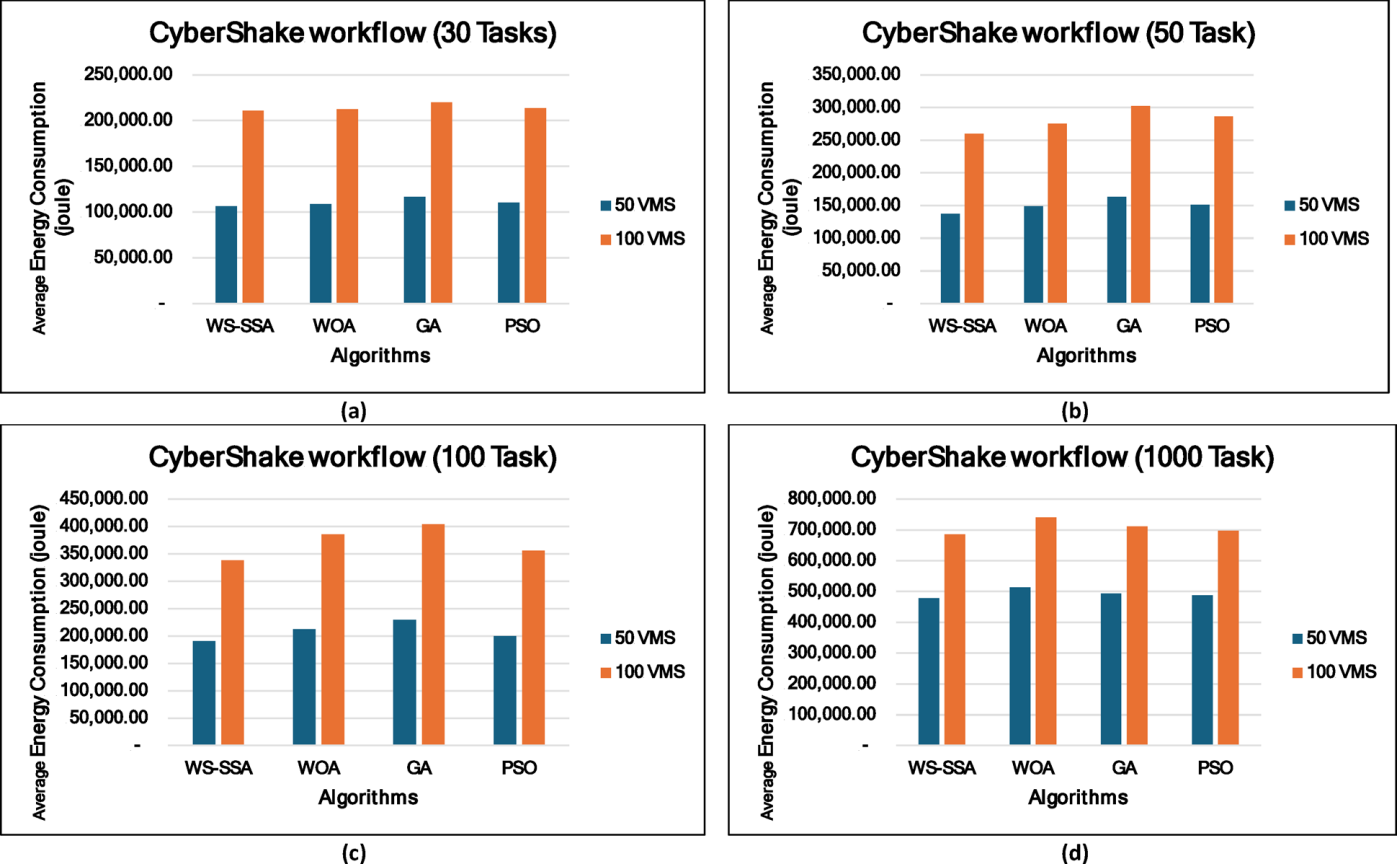
Superiority of the proposed WS-SSA over WOA (GA) (PSO) algorithms in reducing the energy consumption of CyberShake workflows of various sizes and using different VM configurations.

**Fig. 16:**
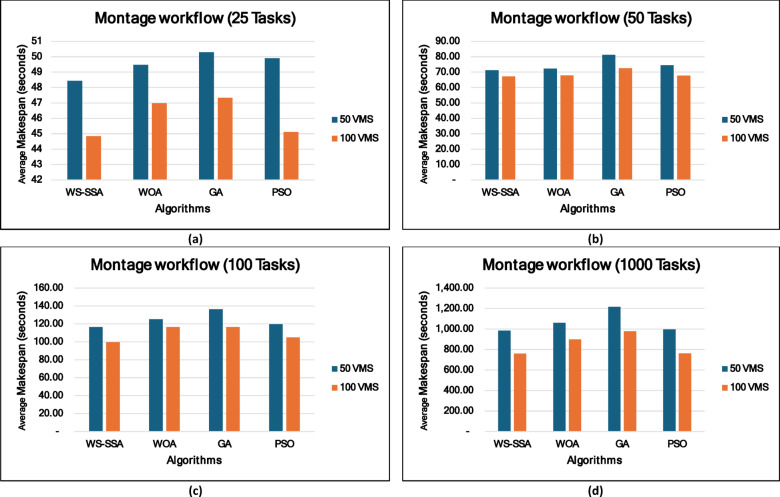
Superiority of the proposed WS-SSA scheduler over WOA (GA) (PSO) algorithms in reducing the makespan of Montage workflows of various sizes and using different VM configurations.

**Fig. 17:**
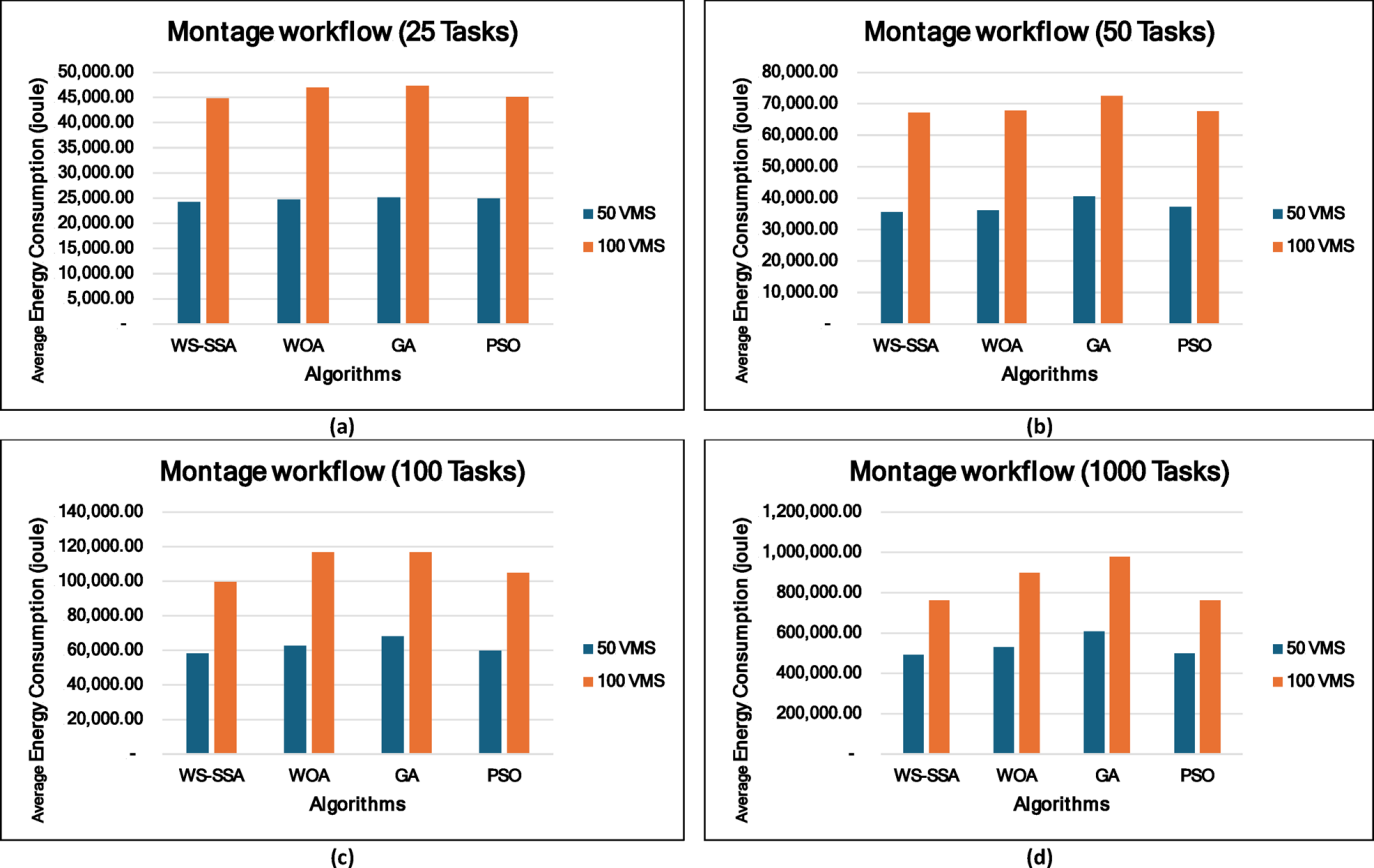
Superiority of the proposed WS-SSA scheduler over WOA (GA) (PSO) algorithms in reducing the energy consumption of Montage workflows of various sizes and using different VM configurations.

**Fig. 18:**
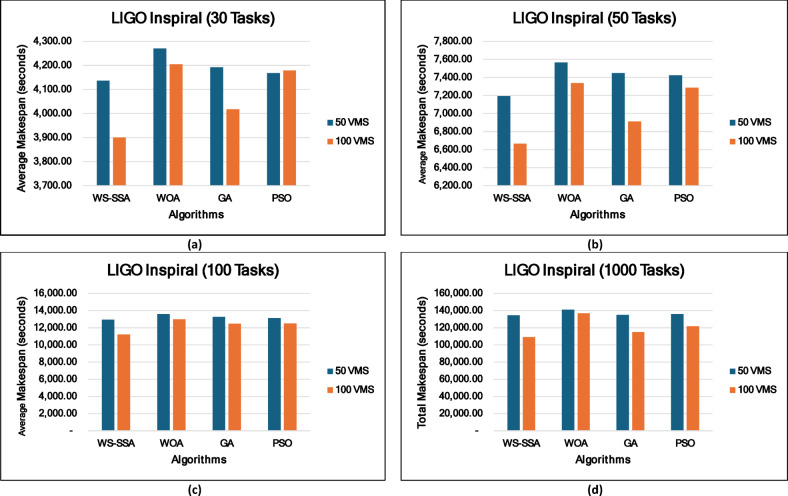
Superiority of the proposed WS-SSA over WOA (GA) (PSO) algorithms in reducing the makespan of the LIGO Inspiral workflows of various sizes and using different VM configurations.

**Fig. 19:**
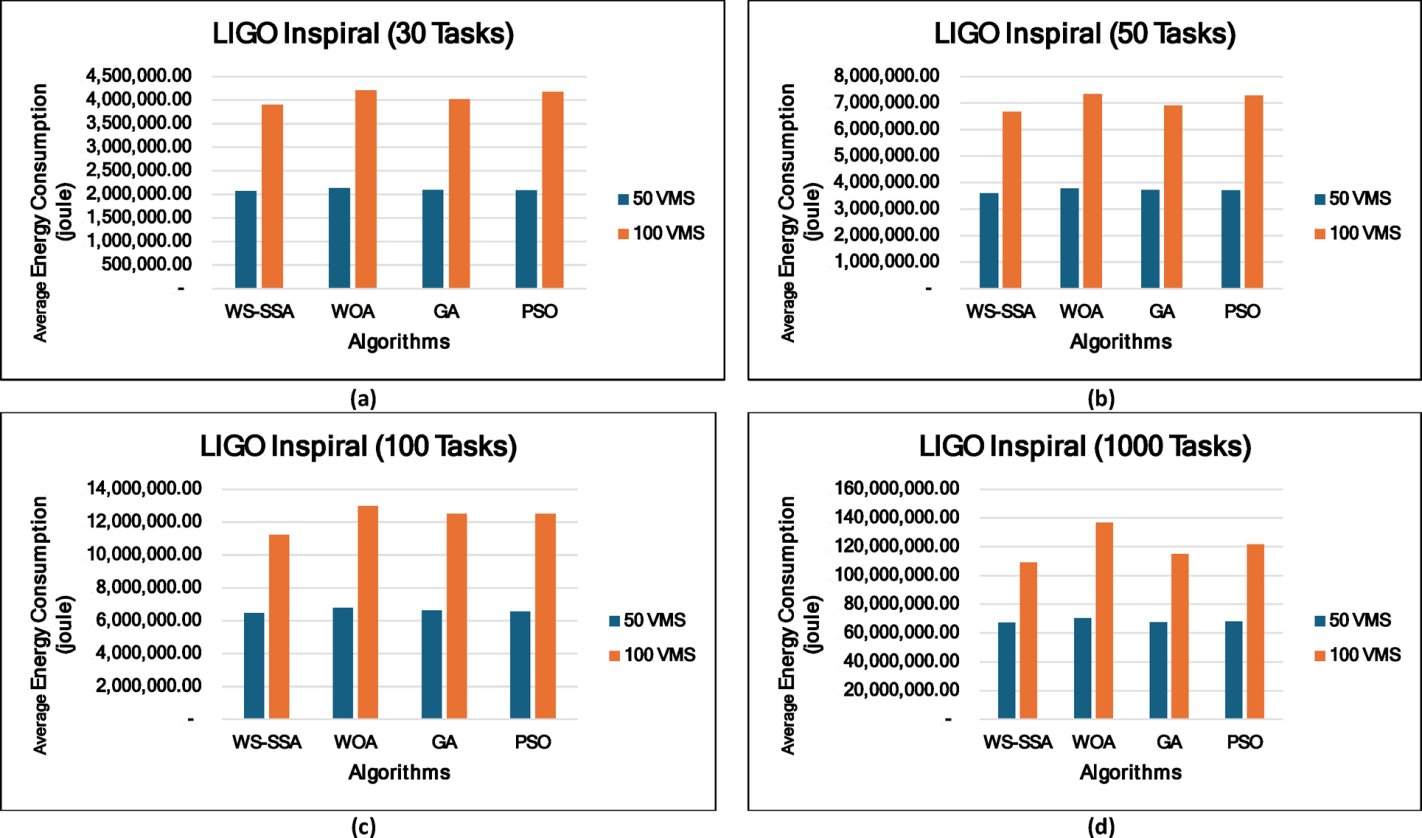
Superiority of the proposed WS-SSA over WOA (GA) (PSO) algorithms in reducing the energy consumption of the LIGO Inspiral workflows of various sizes and using different VM configurations.

**Fig. 20:**
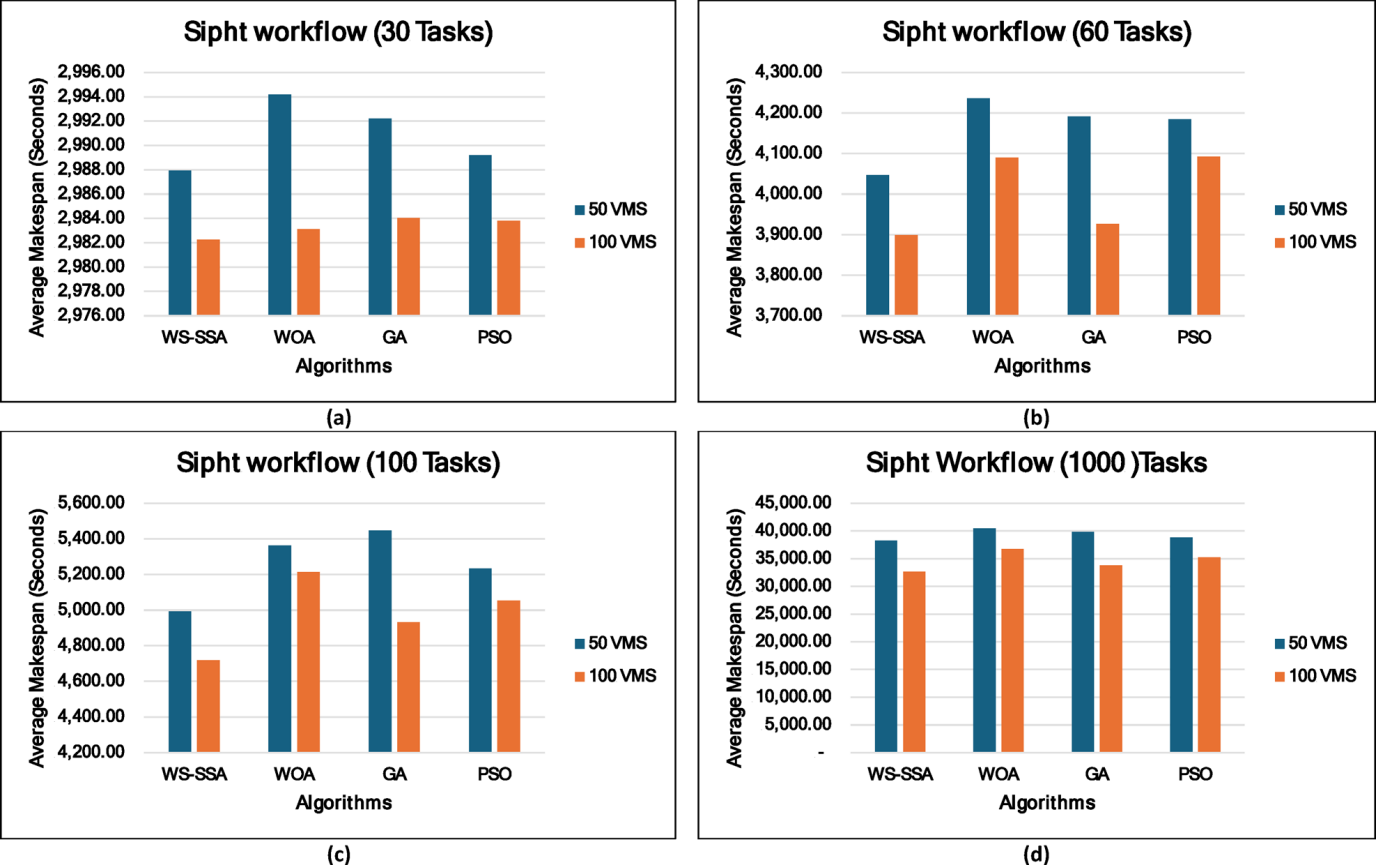
Superiority of the proposed WS-SSA over WOA (GA) (PSO) algorithms in reducing the makespan of the Sipht workflows of various sizes and using different VM configurations.

**Fig. 21:**
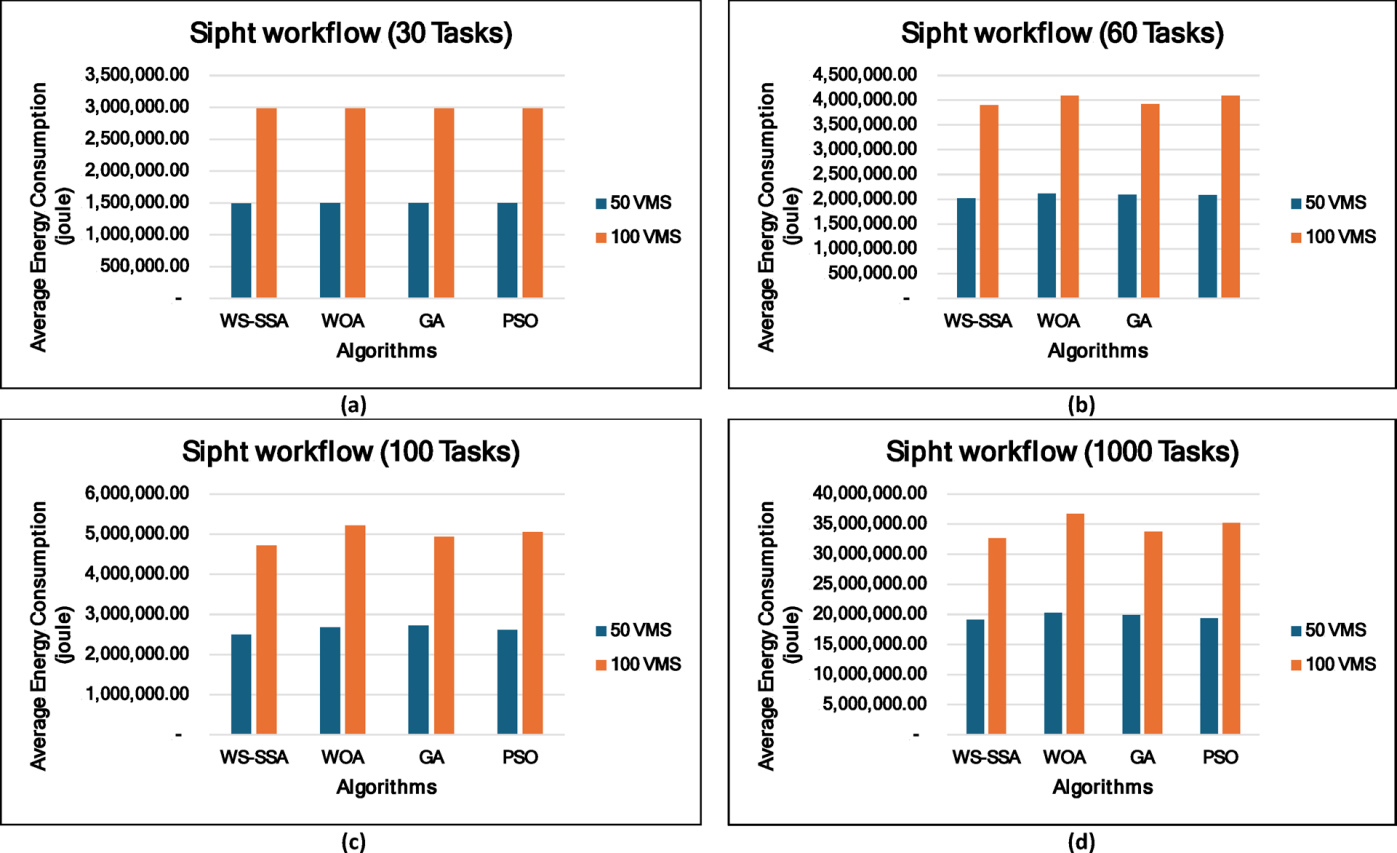
Superiority of the proposed WS-SSA scheduler over WOA (GA) (PSO) algorithms in reducing the energy consumption of Sipht workflows of various sizes and using different VM configurations.

**Fig. 22:**
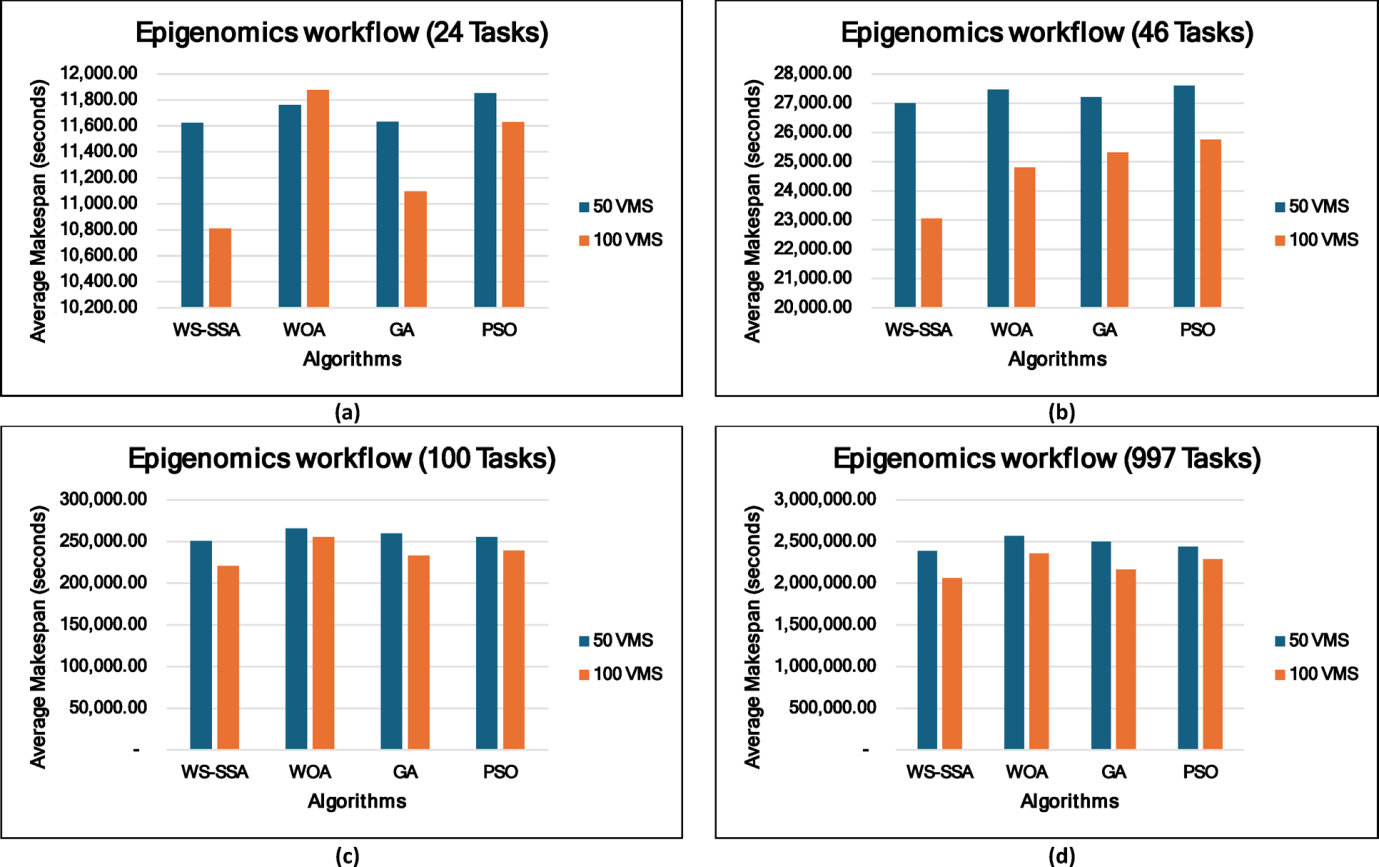
Superiority of the proposed WS-SSA over WOA (GA) (PSO) algorithms in reducing the makespan of the Epigenomics workflows of various sizes and using different VM configurations.

**Fig. 23:**
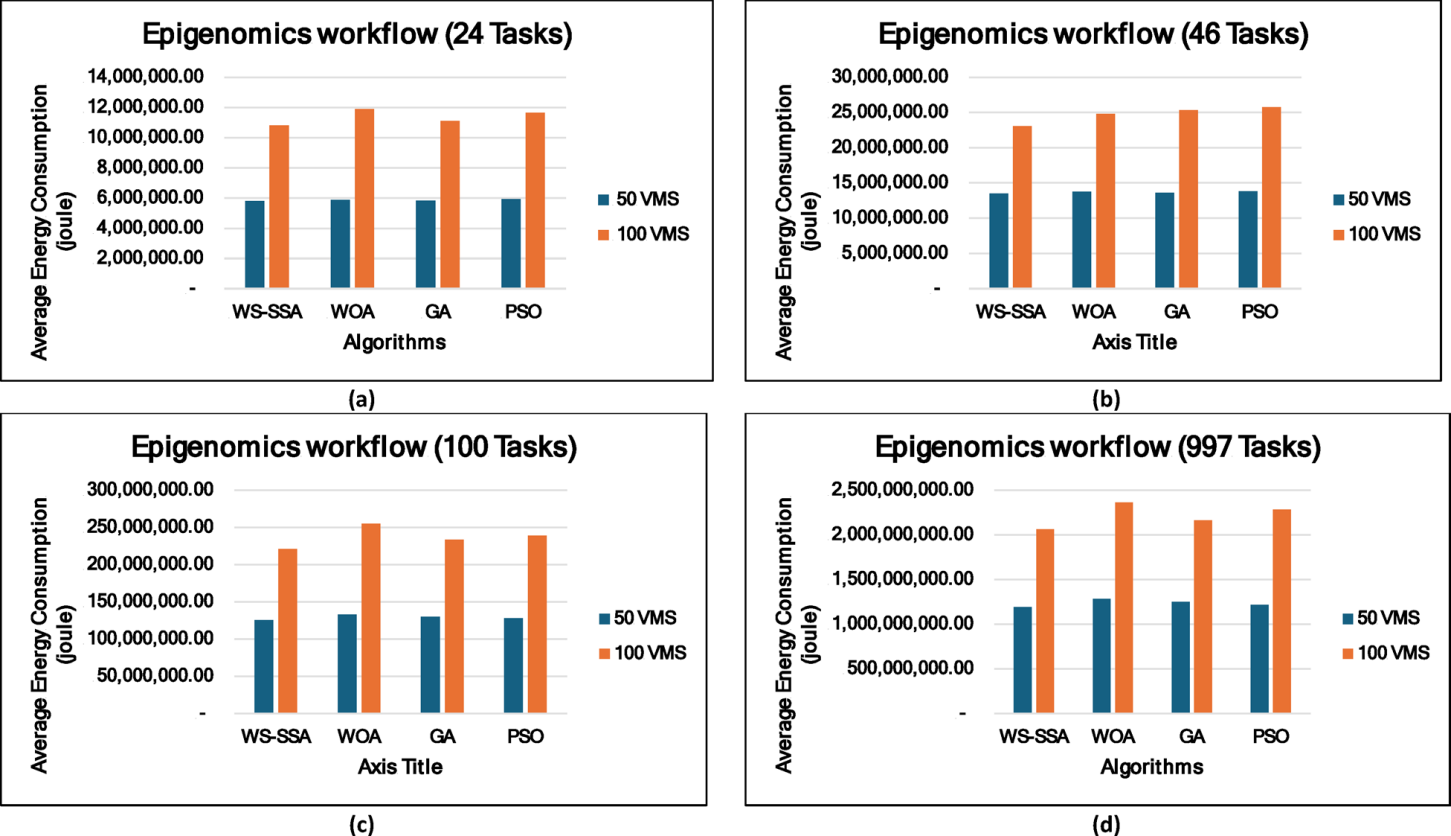
Superiority of the proposed WS-SSA over WOA (GA) (PSO) algorithms in reducing the energy consumption of epigenomics workflows of various sizes and using different VM configurations.

**Table 16:** Computational complexity comparison of heuristic and metaheuristic workflow scheduling algorithms.

Algorithm	Category	Time complexity	Dependency awareness	Remarks
FCFS	Simple heuristic	$\:O\left(n\right)\:$	✗	Minimum computation cost; does not consider either task dependency or VM heterogeneity.
Round Robin	Simple heuristic	$\:O\left(n\right)$	✗	Task allocation is moderate yet inappropriate with dependent workflows.
MCT	Informed heuristic	$\:O(n\:x\:m$)	✓ (partial)	Considers the time of execution on VMs but has no global optimization.
Min–Min	Informed heuristic	$\:O\left({n}^{2}\:x\:m\right)$	✓	Greed strategy; local optima tendencies of large workflows.
Max–Min	Informed heuristic	$\:O\left({n}^{2}\:x\:m\right)$	✓	Enhances the load balance but raises the cost of computation.
PSO	Metaheuristic	$\:O\left(T\:x\:N\:x\:D\right)$	✓	Velocity-based position update; sensitive to inertia weight and acceleration coefficients; competitive on small-to-medium workflows.
GA	Metaheuristic	$\:O\left(T\:x\:N\:x\:D\right)$	✓	Selection, crossover, and mutation operators add per-iteration overhead; susceptible to premature convergence on large scheduling spaces.
WOA	Metaheuristic	$\:O\left(T\:x\:N\:x\:D\right)$	✓	Spiral update involves exponential and trigonometric calculations, adding constant factor overhead per iteration.
WS-SSA	Metaheuristic	$\:O\left(T\:x\:N\:x\:D\right)$	✓	Dual leader–follower update uses only arithmetic operations; minimal parameter sensitivity; consistent convergence with no added asymptotic cost.

**Table 17:** Effect of population size on average makespan (seconds).

Workflow	VMs	Pop = 30	Pop = 50	Pop = 80	Δ (30→80)
CyberShake	50	981.76	980.62	971.36	−1.06%
CyberShake	100	715.28	711.05	715.93	+ 0.09%
Montage	50	926.01	934.00	942.21	+ 1.75%
Montage	100	755.57	762.22	764.78	+ 1.22%
Inspiral	50	133,716	132,914	134,121	+ 0.30%
Inspiral	100	116,494	116,715	115,632	−0.74%
Sipht	50	39,069	39,617	39,180	+ 0.29%
Sipht	100	33,592	33,434	33,485	−0.32%
Epigenomics	50	2,258,884	2,259,428	2,283,679	+ 1.10%
Epigenomics	100	2,098,271	2,108,421	2,122,237	+ 1.14%

**Table 18:** Effect of maximum iterations on average makespan (seconds).

Workflow	VMs	Iter = 100	Iter = 200	Iter = 300	Δ (100→300)
CyberShake	50	975.36	987.35	971.03	−0.44%
CyberShake	100	721.69	711.17	709.39	−1.70%
Montage	50	937.36	928.74	936.12	−0.13%
Montage	100	768.82	752.59	761.16	−1.00%
Inspiral	50	133,796	133,909	133,046	−0.56%
Inspiral	100	116,979	115,924	115,937	−0.89%
Sipht	50	39,083	39,758	39,025	−0.15%
Sipht	100	33,395	33,477	33,640	+ 0.74%
Epigenomics	50	2,269,131	2,267,332	2,265,528	−0.16%
Epigenomics	100	2,116,926	2,107,330	2,104,673	−0.58%

**Fig. 24:**
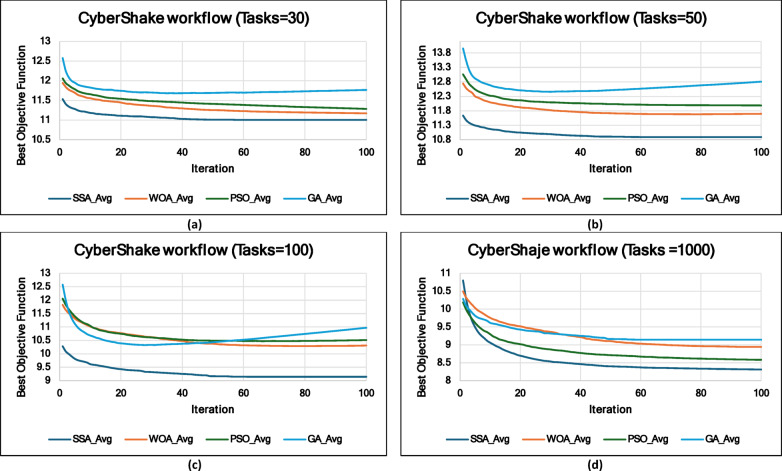
Convergence curves of the proposed discrete SSA, WOA, PSO, and GA on the CyberShake workflow.

**Fig. 25:**
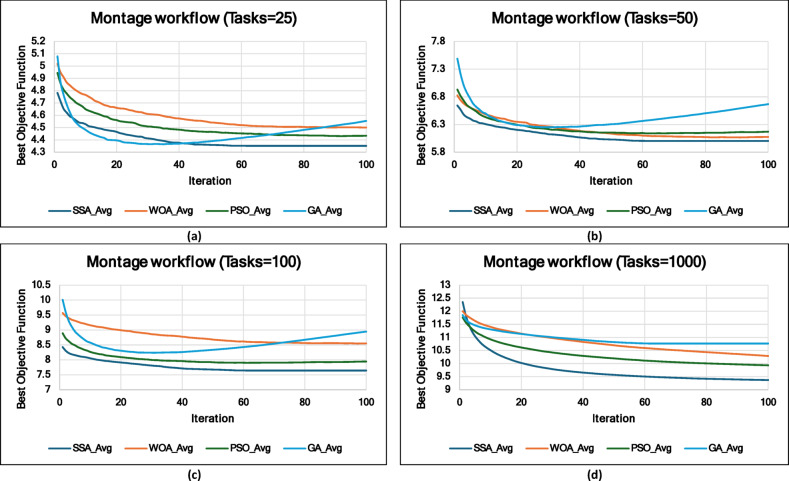
Convergence curves of the proposed discrete SSA, WOA, PSO, and GA on the Montage workflow.

**Fig. 26:**
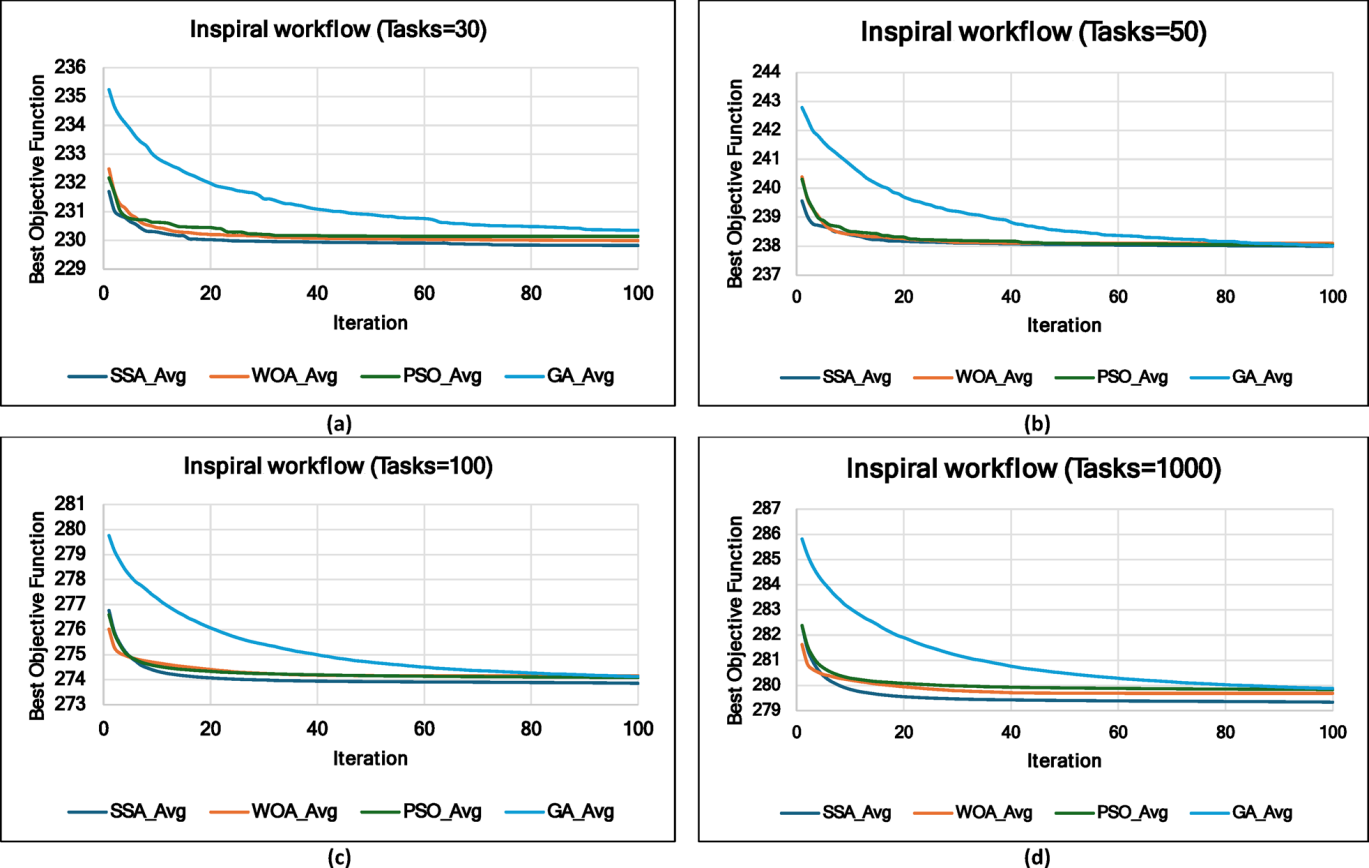
Convergence curves of the proposed discrete SSA, WOA, PSO, and GA on the Inspiral workflow.

**Fig. 27:**
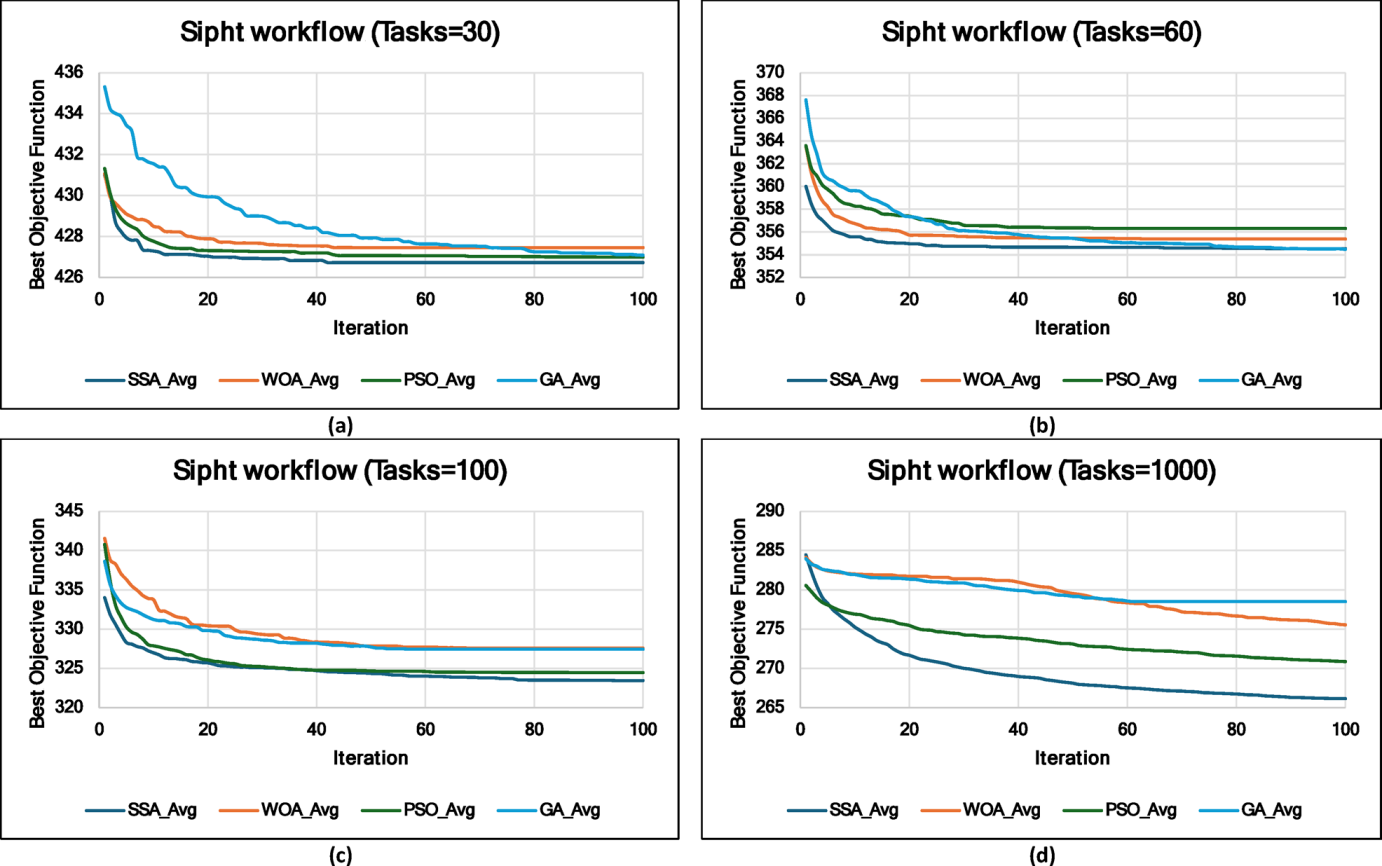
Convergence curves of the proposed discrete SSA, WOA, PSO, and GA on the Sipht workflow.

**Fig. 28:**
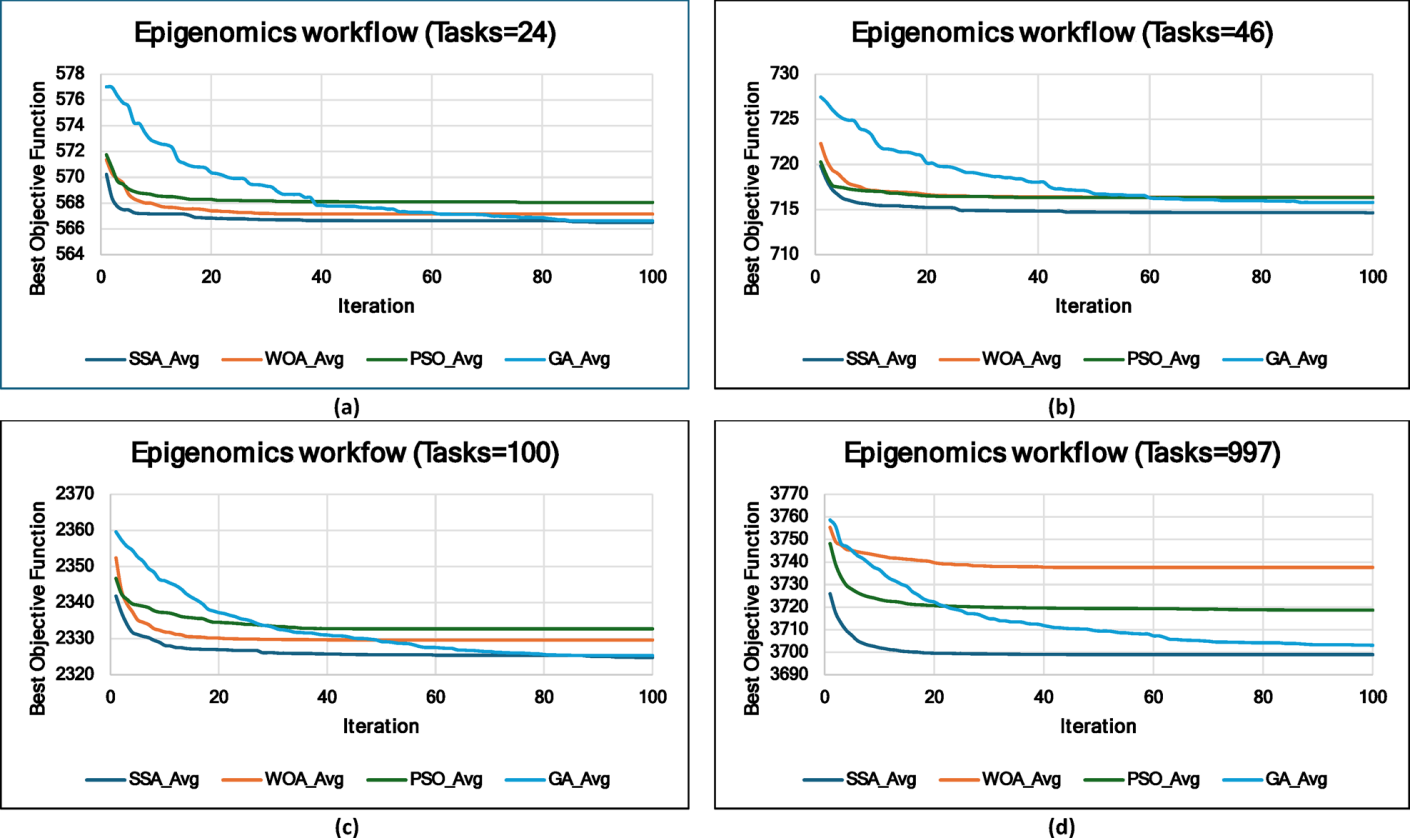
Convergence curves of the proposed discrete SSA, WOA, PSO, and GA on the epigenomics workflow.

**Table 19:** Stochastic variability summary by workflow (*n* = 30 runs).

Workflow	Mean CV (%)	Std CV	Avg 95% CI (%)	Max 95% CI (%)	Variability	Stability
CyberShake	7.82	1.53	5.60	7.44	Low	Stable
Montage	10.31	1.30	7.37	9.06	Low	Stable
Sipht	15.10	5.42	10.80	16.23	Moderate	Acceptable
Inspiral	17.34	4.52	12.40	16.25	High	Marginal
Epigenomics	17.62	1.28	12.61	14.25	High	Marginal

## Conclusion and future work

This paper considers the use of the WS-SSA algorithm in scheduling workflows in cloud computing systems with the aim of reducing makespan. Empirical analysis revealed that the WS-SSA was able to explore the large search space of workflow scheduling problems efficiently and outperformed both baseline heuristics and standard metaheuristic methods in terms of overall time reduction. The excellent performance of the algorithm can be explained by a balanced exploration-exploitation mechanism, simple parameterization, and strong adaptability under simulated variations in task dependencies and resource performance conditions. The findings, therefore, make WS-SSA a promising metaheuristic to makespan oriented workflow scheduling on clouds. There are a number of areas in which future research can be conducted. First, the deadline- and SLA-aware scheduling systems allow the system to address real-time workflow constraints more effectively, which results in increased reliability and user satisfaction. The proposed approach can be extended via a multiobjective Salp Swarm Algorithm (MSSA) framework, which represents a major opportunity for improvement. The MSSA extension allows us to optimize several conflicting goals at once, such as makespan minimization, energy consumption reduction, execution cost optimization, and resource utilization maximization. This multiobjective framework supplies quantifiable sets of Pareto-optimal solutions to cloud users and service providers, which are more realistic and consistent with the complex demands and constraints of modern cloud computing environments. Moreover, a component-level ablation analysis of SSA’s internal mechanisms will be incorporated to provide deeper insight into the relative contribution of each algorithmic component to the observed performance improvements. Finally, the realism of the execution model will be enhanced in future work by incorporating stochastic resource contention, VM startup delays, and shared-resource interference effects, moving beyond the ideal execution isolation assumed in the current WorkflowSim/CloudSim abstraction.

## Supplementary Information

Below is the link to the electronic supplementary material.

Supplementary Material 1

## Data Availability

The synthetic workflow datasets (DAGs) used in this study are publicly available. The experimental results and performance data generated during the simulation are available from the corresponding author upon request.
