# Supplementary material for: WS-SSA: workflow scheduling in cloud computing using salp swarm algorithm
Source: Sci Rep. 2026 Apr 24;16:13402. doi: 10.1038/s41598-026-48037-w (PMC13109423; doi:10.1038/s41598-026-48037-w)
Supplement: Supplementary file 1 — Supplementary Material 1 [file 41598_2026_48037_MOESM1_ESM.docx]

| **Workflow** | **VM** | **FCFS** | **MCT** | **MIN-MIN** | **MAX-MIN** | **Round Robin** | **WOA** | **WS-SSA** |
| --- | --- | --- | --- | --- | --- | --- | --- | --- |
| **CyberShake_30** | **5** | 471.43 | 469.19 | 387.5 | 644.1 | 404.8 | 383.05 | 353.13 |
|  | **10** | 419.97 | 387.72 | 329.82 | 458.03 | 368.72 | 319.7 | 284.54 |
|  | **15** | 382.58 | 336.18 | 297.44 | 300.52 | 335.1 | 276.97 | 254.63 |
| **CyberShake_50** | **5** | 737.01 | 708.13 | 1,681.78 | 1,270.19 | 841.43 | 654.23 | 621.26 |
|  | **10** | 641.67 | 441.91 | 634.72 | 710.95 | 739.28 | 420.36 | 391.23 |
|  | **15** | 555.09 | 384.18 | 584.92 | 400.91 | 631.60 | 355.55 | 329.40 |
| **CyberShake_100** | **5** | 1,964.70 | 1,467.19 | 2,600.13 | 1,953.51 | 2,070.55 | 1,174.64 | 1,064.18 |
|  | **10** | 1,314.59 | 809.52 | 1,417.76 | 948.14 | 1,642.93 | 779.84 | 723.98 |
|  | **15** | 1,296.21 | 731.34 | 743.20 | 640.15 | 1,033.11 | 596.27 | 533.40 |
| **CyberShake_1000** | **5** | 9,240.01 | 8,192.21 | 7,331.39 | 8,813.27 | 6,312.47 | 5,885.52 | 5,204.42 |
|  | **10** | 4,048.09 | 3,780.04 | 4,589.67 | 4,157.45 | 4,296.36 | 3,599.10 | 3,222.70 |
|  | **15** | 3,093.57 | 3,524.95 | 3,174.79 | 2,540.88 | 3,069.83 | 2,374.35 | 2,144.80 |
| **Sipht_30** | **5** | 15,526.68 | 8,410.88 | 15,432.60 | 5,809.67 | 12,898.13 | 4,915.34 | 4,577.00 |
|  | **10** | 5,383.64 | 6,680.11 | 5,556.20 | 4,777.66 | 11,339.01 | 4,396.00 | 3,886.53 |
|  | **15** | 4,715.04 | 6,109.56 | 5,034.67 | 4,365.18 | 8,033.82 | 4,173.48 | 3,793.11 |
| **Sipht_60** | **5** | 9,562.94 | 28,100.43 | 20,162.10 | 6,304.69 | 7,571.79 | 5,910.92 | 5,416.82 |
|  | **10** | 9,296.68 | 20,092.11 | 15,658.03 | 5,652.11 | 6,651.97 | 5,377.50 | 4,790.28 |
|  | **15** | 8,066.47 | 6,667.40 | 7,560.94 | 5,000.45 | 6,009.82 | 4,721.79 | 4,457.13 |
| **Sipht_100** | **5** | 8,739.06 | 9,053.40 | 18,184.37 | 15,437.09 | 10,562.61 | 8,157.07 | 7,673.77 |
|  | **10** | 8,059.83 | 7,701.04 | 16,531.58 | 4,932.54 | 9,321.10 | 4,587.61 | 4,136.98 |
|  | **15** | 7,003.29 | 6,920.55 | 13,410.25 | 4,370.40 | 8,297.21 | 4,025.03 | 3,808.52 |
| **Sipht_1000** | **5** | 52,031.99 | 66,925.90 | 54,719.81 | 84,946.98 | 59,606.16 | 48,209.67 | 44,593.68 |
|  | **10** | 43,766.10 | 33,809.84 | 40,409.05 | 31,097.23 | 33,482.30 | 29,913.40 | 26,472.11 |
|  | **15** | 29,395.16 | 25,667.98 | 24,784.54 | 24,937.51 | 29,045.86 | 22,900.27 | 20,536.80 |
| **Montage_25** | **5** | 242.07 | 192.92 | 247.99 | 231.64 | 74.28 | 70.15 | 62.58 |
|  | **10** | 229.18 | 88.1 | 91.14 | 63.82 | 62.26 | 61.21 | 57.04 |
|  | **15** | 159.41 | 79.41 | 57.09 | 61.49 | 53.92 | 50.72 | 45.06 |
| **Montage_50** | **5** | 157.83 | 148.72 | 362.01 | 432.02 | 433.77 | 141.8 | 126.01 |
|  | **10** | 140.07 | 134.08 | 172.28 | 174.42 | 179.88 | 123.81 | 114.31 |
|  | **15** | 122.74 | 107.17 | 163.97 | 143.96 | 154.69 | 101.51 | 89.63 |
| **Montage_100** | **5** | 545.66 | 335.14 | 333.23 | 286.2 | 289.27 | 264.38 | 240.67 |
|  | **10** | 266.71 | 264.22 | 290.11 | 247.36 | 266.11 | 228.64 | 212.28 |
|  | **15** | 243.02 | 239.75 | 144.95 | 210.97 | 192.55 | 138.54 | 130.15 |
| **Montage_1000** | **5** | 4,660.75 | 4,408.34 | 4,191.68 | 13,907.52 | 5,943.59 | 4,020.49 | 3,693.05 |
|  | **10** | 2,182.75 | 2,503.41 | 3,290.74 | 2,760.92 | 5,461.55 | 2,012.34 | 1,781.32 |
|  | **15** | 1,915.25 | 1,752.64 | 1,772.64 | 1,636.28 | 2,671.94 | 1,538.69 | 1,363.28 |
| **Inspiral_30** | **5** | 4,767.20 | 4,798.04 | 5,681.95 | 5,632.72 | 5,074.38 | 4,454.73 | 4,063.51 |
|  | **10** | 4,173.02 | 2,989.48 | 3,745.81 | 2,981.67 | 4,365.51 | 2,839.02 | 2,575.12 |
|  | **15** | 3,650.97 | 1,782.05 | 1,722.68 | 1,538.93 | 3,956.47 | 1,431.01 | 1,318.57 |
| **Inspiral_50** | **5** | 4,070.09 | 7,811.78 | 8,209.63 | 8,659.53 | 10,465.02 | 3,849.61 | 3,500.25 |
|  | **10** | 3,963.67 | 6,655.63 | 7,844.58 | 6,968.11 | 7,159.53 | 3,741.02 | 3,395.72 |
|  | **15** | 3,400.48 | 3,105.18 | 2,287.70 | 4,006.13 | 6,806.15 | 2,197.08 | 1,953.30 |
| **Inspiral_100** | **5** | 8,357.98 | 8,342.92 | 8,961.93 | 8,436.84 | 12,052.74 | 7,891.52 | 7,411.11 |
|  | **10** | 4,520.31 | 4,870.70 | 5,486.06 | 5,203.66 | 4,148.38 | 3,966.05 | 3,698.05 |
|  | **15** | 4,151.53 | 4,346.11 | 3,629.93 | 4,644.46 | 3,807.63 | 3,441.93 | 3,083.99 |
| **Inspiral_1000** | **5** | 102,430.54 | 70,487.78 | 119,548.16 | 64,415.71 | 93,207.34 | 60,008.19 | 56,671.99 |
|  | **10** | 35,199.59 | 38,797.45 | 42,640.67 | 48,704.90 | 67,393.83 | 32,697.09 | 29,847.67 |
|  | **15** | 27,880.76 | 33,713.20 | 32,426.02 | 36,282.81 | 27,769.11 | 26,744.12 | 25,353.74 |
| **Epigenomics_24** | **5** | 34,204.93 | 30,335.55 | 33,335.54 | 27,873.72 | 39,832.78 | 21,822.95 | 20,327.39 |
|  | **10** | 31,360.39 | 7,283.57 | 13,712.55 | 11,208.98 | 10,576.26 | 6,939.70 | 6,480.77 |
|  | **15** | 17,881.17 | 6,979.21 | 7,628.56 | 6,440.95 | 9,224.31 | 6,064.89 | 5,730.51 |
| **Epigenomics_46** | **5** | 23,950.39 | 32,560.43 | 23,428.17 | 36,406.48 | 16,872.67 | 15,612.08 | 13,928.03 |
|  | **10** | 21,212.97 | 19,440.95 | 19,811.05 | 19,113.41 | 15,234.98 | 14,135.10 | 12,774.36 |
|  | **15** | 18,762.87 | 11,622.05 | 9,227.33 | 15,116.20 | 13,558.60 | 8,775.62 | 7,741.20 |
| **Epigenomics_100** | **5** | 184,812.52 | 226,761.95 | 412,759.36 | 417,361.59 | 420,020.53 | 177,412.85 | 159,054.80 |
|  | **10** | 80,469.46 | 176,390.62 | 236,234.95 | 102,616.71 | 106,404.04 | 75,419.02 | 70,125.10 |
|  | **15** | 73,371.39 | 131,752.05 | 160,481.56 | 94,012.01 | 90,918.22 | 70,040.57 | 64,280.15 |
| **Epigenomics_997** | **5** | 1,790,442.55 | 1,366,324.79 | 1,232,850.97 | 1,221,052.55 | 2,076,879.25 | 1,164,823.72 | 1,027,547.75 |
|  | **10** | 905,211.33 | 626,726.93 | 778,340.56 | 717,913.61 | 819,792.40 | 588,791.37 | 558,414.30 |
|  | **15** | 587,170.26 | 452,868.10 | 464,372.16 | 692,182.51 | 737,835.30 | 423,374.91 | 384,499.02 |

Table 1.Summary of Makespan values of the Pegasus Workflows by all algorithms (Experiment1).

| **Workflow** | **VM** | **FCFS** | **MCT** | **MIN-MIN** | **MAX-MIN** | **Round Robin** | **WOA** | **WS-SSA** |
| --- | --- | --- | --- | --- | --- | --- | --- | --- |
| **CyberShake_30** | **5** | 11,785.82 | 11,729.67 | 9,687.51 | 16,102.47 | 10,120.02 | 9,576.21 | 9,258.50 |
|  | **10** | 46,325.18 | 19,386.01 | 20,870.43 | 22,901.64 | 73,276.32 | 18,261.00 | 17,146.95 |
|  | **15** | 64,693.00 | 39,090.33 | 41,270.09 | 25,437.84 | 79,291.64 | 24,567.05 | 23,343.71 |
| **CyberShake_50** | **5** | 18,425.24 | 17,703.34 | 42,044.40 | 31,754.66 | 21,035.79 | 17,198.63 | 17,149.96 |
|  | **10** | 74,228.84 | 22,095.58 | 47,644.56 | 35,547.44 | 43,582.30 | 21,599.70 | 20,951.68 |
|  | **15** | 84,139.20 | 45,526.34 | 53,245.70 | 38,714.43 | 77,031.05 | 37,608.41 | 35,427.99 |
| **CyberShake_100** | **5** | 49,117.54 | 36,679.71 | 65,003.31 | 48,837.84 | 51,763.69 | 29,366.01 | 28,465.88 |
|  | **10** | 65,729.50 | 40,476.02 | 70,888.21 | 54,484.48 | 82,146.27 | 38,505.38 | 37,243.01 |
|  | **15** | 97,216.04 | 54,850.44 | 79,178.88 | 60,310.86 | 89,087.44 | 52,525.26 | 50,758.48 |
| **CyberShake_1000** | **5** | 231,000.26 | 204,805.27 | 183,284.81 | 220,331.81 | 157,811.84 | 152,980.60 | 143,697.00 |
|  | **10** | 260,626.26 | 224,751.13 | 229,483.67 | 242,720.29 | 214,817.86 | 204,392.05 | 192,967.19 |
|  | **15** | 295,683.26 | 264,371.44 | 238,109.48 | 267,547.56 | 230,236.98 | 216,471.06 | 208,140.36 |
| **Sipht_30** | **5** | 388,166.90 | 210,272.06 | 385,814.91 | 145,241.82 | 322,453.19 | 122,883.56 | 117,501.04 |
|  | **10** | 419,793.25 | 334,005.65 | 441,901.65 | 238,883.09 | 566,950.62 | 231,052.80 | 222,165.70 |
|  | **15** | 654,382.89 | 527,709.40 | 475,555.89 | 373,695.52 | 602,536.28 | 359,031.37 | 332,903.51 |
| **Sipht_60** | **5** | 239,073.57 | 702,510.71 | 504,052.49 | 157,617.15 | 189,294.75 | 152,716.87 | 140,590.86 |
|  | **10** | 464,833.80 | 1,004,605.61 | 782,901.37 | 282,605.69 | 610,567.04 | 271,077.29 | 257,087.30 |
|  | **15** | 784,164.91 | 1,142,116.92 | 848,327.09 | 375,034.04 | 619,094.54 | 355,278.38 | 330,179.07 |
| **Sipht_100** | **5** | 218,476.45 | 226,334.92 | 454,609.30 | 385,927.24 | 264,065.16 | 210,480.87 | 201,351.13 |
|  | **10** | 402,991.58 | 757,668.85 | 1,078,117.68 | 427,242.59 | 1,114,626.12 | 399,973.30 | 376,631.68 |
|  | **15** | 949,886.26 | 778,379.17 | 1,204,188.27 | 475,480.28 | 1,118,235.48 | 457,307.71 | 431,653.85 |
| **Sipht_1000** | **5** | 1,300,799.64 | 1,673,147.61 | 1,367,995.25 | 2,123,674.46 | 1,490,154.05 | 1,238,060.90 | 1,185,949.19 |
|  | **10** | 2,188,304.80 | 1,690,491.75 | 2,020,452.33 | 2,434,592.33 | 1,674,115.08 | 1,625,716.12 | 1,520,870.10 |
|  | **15** | 2,204,636.82 | 1,925,098.52 | 2,199,505.21 | 2,684,689.11 | 2,802,851.08 | 1,825,956.30 | 1,736,259.12 |
| **Montage_25** | **5** | 6,051.75 | 4,823.00 | 6,199.70 | 5,791.09 | 1,856.88 | 1,779.93 | 1,661.74 |
|  | **10** | 11,458.98 | 5,474.10 | 7,123.55 | 6,611.05 | 15,714.03 | 4,563.25 | 4,261.67 |
|  | **15** | 11,955.88 | 6,874.65 | 7,939.47 | 7,567.60 | 17,705.07 | 6,508.43 | 6,231.36 |
| **Montage_50** | **5** | 3,945.74 | 3,718.08 | 9,050.32 | 10,800.61 | 10,844.15 | 3,512.85 | 3,282.67 |
|  | **10** | 13,368.72 | 8,219.90 | 10,250.96 | 12,020.68 | 11,718.26 | 7,979.76 | 7,392.94 |
|  | **15** | 14,704.14 | 9,314.32 | 12,297.87 | 13,505.97 | 17,011.28 | 8,806.68 | 8,351.88 |
| **Montage_100** | **5** | 13,641.46 | 8,378.50 | 8,330.75 | 7,155.10 | 7,231.68 | 6,977.46 | 6,632.90 |
|  | **10** | 14,996.43 | 13,210.97 | 20,059.75 | 19,441.01 | 21,895.51 | 12,491.54 | 11,588.59 |
|  | **15** | 52,003.03 | 23,612.62 | 22,154.32 | 23,257.74 | 24,802.91 | 21,104.51 | 19,655.13 |
| **Montage_1000** | **5** | 116,518.68 | 110,208.45 | 104,792.12 | 347,687.97 | 148,589.86 | 99,767.80 | 91,807.98 |
|  | **10** | 130,291.28 | 125,170.47 | 164,536.88 | 377,393.27 | 273,077.41 | 120,088.15 | 113,588.73 |
|  | **15** | 143,643.68 | 131,447.95 | 182,631.49 | 411,238.08 | 306,643.75 | 126,471.06 | 119,349.78 |
| **Inspiral_30** | **5** | 119,180.08 | 119,950.94 | 142,048.76 | 140,817.98 | 126,859.62 | 115,818.52 | 110,865.27 |
|  | **10** | 367,873.52 | 149,473.95 | 187,290.27 | 149,083.62 | 278,197.31 | 141,582.44 | 134,546.63 |
|  | **15** | 422,767.41 | 167,852.62 | 210,534.96 | 169,851.67 | 296,735.45 | 161,036.08 | 153,295.18 |
| **Inspiral_50** | **5** | 101,752.32 | 195,294.54 | 205,240.69 | 216,488.19 | 261,625.39 | 98,380.98 | 92,442.81 |
|  | **10** | 198,183.46 | 639,361.50 | 392,229.12 | 348,405.40 | 357,976.38 | 189,948.54 | 176,581.31 |
|  | **15** | 814,917.56 | 708,052.22 | 446,076.47 | 387,821.07 | 510,460.88 | 375,620.20 | 356,409.77 |
| **Inspiral_100** | **5** | 208,949.58 | 208,573.10 | 224,048.24 | 210,920.95 | 301,318.46 | 198,716.88 | 187,481.38 |
|  | **10** | 226,015.65 | 243,535.01 | 274,302.97 | 260,182.76 | 331,580.38 | 219,732.78 | 208,203.70 |
|  | **15** | 519,944.59 | 480,695.51 | 296,519.57 | 464,743.59 | 483,047.25 | 287,491.41 | 277,419.93 |
| **Inspiral_1000** | **5** | 2,560,763.50 | 1,762,194.39 | 2,988,704.07 | 1,610,392.66 | 2,330,183.47 | 1,574,632.09 | 1,494,243.99 |
|  | **10** | 2,803,764.61 | 1,939,872.73 | 3,428,892.60 | 2,435,244.88 | 3,369,691.43 | 1,857,982.86 | 1,768,175.35 |
|  | **15** | 3,157,343.30 | 2,528,490.03 | 3,708,171.78 | 2,721,211.11 | 3,784,746.94 | 2,384,545.18 | 2,262,110.38 |
| **Epigenomics_24** | **5** | 855,123.27 | 700,388.75 | 758,388.53 | 696,842.94 | 995,819.55 | 545,573.64 | 502,586.67 |
|  | **10** | 1,955,451.02 | 830,969.78 | 857,644.29 | 796,400.99 | 1,133,106.82 | 749,741.31 | 710,501.71 |
|  | **15** | 2,135,073.41 | 902,963.19 | 977,289.44 | 865,719.01 | 1,256,503.90 | 827,427.05 | 783,663.26 |
| **Epigenomics_46** | **5** | 598,759.70 | 814,010.67 | 585,704.23 | 910,162.11 | 421,816.74 | 404,550.66 | 373,613.67 |
|  | **10** | 1,588,867.36 | 972,047.35 | 990,552.54 | 955,670.28 | 852,464.84 | 819,111.51 | 782,879.36 |
|  | **15** | 2,091,260.89 | 1,094,373.79 | 1,094,194.30 | 1,133,714.74 | 2,138,784.11 | 1,062,720.95 | 1,011,232.41 |
| **Epigenomics_100** | **5** | 4,620,312.96 | 5,669,048.85 | 10,318,984.08 | 10,434,039.75 | 10,500,513.34 | 4,479,331.89 | 4,317,505.84 |
|  | **10** | 5,186,087.93 | 8,819,530.85 | 11,811,747.56 | 11,942,073.06 | 11,539,576.77 | 4,992,590.03 | 4,682,272.65 |
|  | **15** | 7,917,342.33 | 9,881,403.53 | 12,036,116.99 | 12,803,296.67 | 13,191,437.82 | 7,693,175.58 | 7,267,860.01 |
| **Epigenomics_997** | **5** | 44,761,063.63 | 34,158,119.74 | 30,821,274.36 | 30,526,313.73 | 51,921,981.29 | 29,024,462.95 | 27,468,921.71 |
|  | **10** | 45,260,566.52 | 36,906,552.94 | 38,917,027.96 | 35,895,680.25 | 56,657,975.84 | 33,825,213.06 | 32,328,377.00 |
|  | **15** | 49,957,679.01 | 39,926,862.10 | 43,244,158.25 | 51,913,688.11 | 61,739,640.41 | 38,168,277.41 | 36,681,473.77 |

Table 2. Summary of Energy Consumption values of the Pegasus Workflows by all algorithms (experiment1)
